# Magnetic resonance imaging in multiple sclerosis animal models: A systematic review, meta-analysis, and white paper

**DOI:** 10.1016/j.nicl.2020.102371

**Published:** 2020-08-02

**Authors:** Benjamin V. Ineichen, Pascal Sati, Tobias Granberg, Martina Absinta, Nathanael J. Lee, Jennifer A. Lefeuvre, Daniel S. Reich

**Affiliations:** a Translational Neuroradiology Section, National Institute of Neurological Disorders and Stroke (NINDS), National Institutes of Health (NIH), Bethesda, MD, United States; b Department of Clinical Neuroscience, Karolinska Institutet, Stockholm, Sweden Division of Neuroradiology, Department of Radiology, Karolinska University Hospital, Stockholm, Sweden

**Keywords:** Systematic review, Meta-analysis, Multiple sclerosis (MS), Magnetic resonance imaging (MRI), Animal models, Guidelines

## Abstract

Magnetic resonance imaging (MRI) is the most important paraclinical tool for assessing drug response in multiple sclerosis (MS) clinical trials. As such, MRI has also been widely used in preclinical research to investigate drug efficacy and pathogenic aspects in MS animal models. Keeping track of all published preclinical imaging studies, and possible new therapeutic approaches, has become difficult considering the abundance of studies. Moreover, comparisons between studies are hampered by methodological differences, especially since small differences in an MRI protocol can lead to large differences in tissue contrast. We therefore provide a comprehensive qualitative overview of preclinical MRI studies in the field of neuroinflammatory and demyelinating diseases, aiming to summarize experimental setup, MRI methodology, and risk of bias. We also provide estimates of the effects of tested therapeutic interventions by a meta-analysis. Finally, to improve the standardization of preclinical experiments, we propose guidelines on technical aspects of MRI and reporting that can serve as a framework for future preclinical studies using MRI in MS animal models. By implementing these guidelines, clinical translation of findings will be facilitated, and could possibly reduce experimental animal numbers.

## Highlights

• This is an overview of preclinical MRI studies in neuroinflammatory diseases.
• We summarized experimental setup, MRI methodology, and risk of bias of these studies.
• We propose guidelines to improve standardization of preclinical MRI studies.
• Implementing these reporting guidelines could facilitate clinical translation.
• This study can serve as a framework for future preclinical studies using MRI.

## 1 Introduction

A growing number of large cooperatives, including the Alzheimer's Disease Neuroimaging Initiative (ADNI) and the Human Connectome Project (HPC) (Petersen et al., 2010, Van Essen et al., 2013) aim to standardize reporting on neuroimaging in humans. Whereas standardized reporting on neuroimaging in clinical research — including the use of magnetic resonance imaging (MRI) as a fundamental tool in diagnosis and monitoring of multiple sclerosis (MS) — has received much attention, no such attempts have been made in preclinical neuroimaging research. This gap is surprising since MRI is also widely used in preclinical research to screen for drug efficacy and to investigate pathogenic aspects in animal models, especially in MS animal models, in which both inflammatory and demyelinating pathology are readily detectable using MRI. One concern is that differences in experimental MRI scanning and reporting on technical imaging details can impede comparisons between studies. A comprehensive reporting of methodological details is also key for potential replication of findings (Kilkenny et al., 2010) — an issue that is receiving a great deal of attention in preclinical research (Justice and Dhillon, 2016, Kilkenny et al., 2010, Steward and Balice-Gordon, 2014). Thus, improved reporting of methodological imaging details can maximize the availability and utility of the information gained from every animal experiment, which can ultimately prevent unnecessary animal experiments in the future. Finally, keeping track of the abundance of preclinical MS neuroimaging studies so far published has proven difficult.

Therefore, we set out to provide a comprehensive overview of preclinical MRI studies in the field of neuroinflammatory and demyelinating diseases, summarizing experimental setup, MRI methodology, and risk of bias. Through a meta-analysis, we also investigated the efficacy of assessed therapeutic approaches using MRI outcome measures and histological measures of disease activity in MS animal models. In order to increase standardization of experiments, we propose minimal reporting guidelines on technical aspects and experimental setup for future preclinical MRI studies, with the goal of improving successful translation of preclinical findings for potential therapeutic interventions for MS.

## 2 Materials and methods

This systematic review summarizes preclinical studies assessing therapies and/or pathogenic aspects of MS in corresponding animal models using MRI. The inclusion criteria and method of analysis were specified in advance and documented in a protocol, which was published on PROSPERO (registration number: CRD42019134302). We used the Preferred Reporting Items for Systematic Reviews and Meta-Analysis (PRISMA) Guidelines (Moher et al., 2015).

### 2.1 Search strategy and paper selection

A comprehensive search string to identify publications assessing MRI in MS animal models was generated. The following databases were searched for matches: EMBASE, go3R, Medline, PubMed, Scopus, and Web of Science (last search 01 May 2020). See Supplementary search string for the exact string. All animal species, publication dates, and languages were included in the database search.

Publications were included in this systematic review if they met the following inclusion criteria: (1) the publication was an original peer reviewed full publication that published unique data; and, (2) since MS animal models are generally defined by neuroinflammatory (e.g. experimental autoimmune encephalomyelitis or Theiler’s murine encephalomyelitis) and/or demyelinating pathology (e.g. cuprizone or lysolecithin), the publication used an animal model with a neuroinflammatory/demyelinating pathological substrate in conjunction with any MRI outcome.

Publications were included in the meta-analysis if they met the following inclusion criteria in addition to the ones listed above: (3) the publication contained at least one adequate control group (i.e. vehicle or no treatment); (4) an outcome measure related to MRI was used; and (5) the publication provided an effect measure, animal numbers, and a measure of variability for the respective experimental groups.

Publications were screened for relevance by one reviewer. Reviews were excluded but used as a source for potential studies and for discussion.

### 2.2 Data extraction

The following study characteristics were extracted from the full-texts by two independent reviewers: (1) parameters on model organisms and disease model: type of animal model, tested intervention, application regimen, species, strain, sex, housing conditions, weight, and number of animals per group; (2) parameters on MRI scanning: anesthesia, technical details on MRI scanner (supplier, coils, gradients, magnetic field strength), technical details on MR imaging parameters (pulse sequence, echo/repetition time, field of view, matrix size, and others), contrast agent type, and dosage. As study outcome measures, we extracted the mean and variance (standard deviation [SD]) or standard error of the mean [SEM]) of all available MRI outcome parameters. BVI checked if all data were extracted correctly. Disagreement between the two reviewers was solved by jointly assessing the data in the publications and coming to a consensus. The inter-rater agreement was 71% for MRI outcomes.

When possible, data were extracted from text or tables; if not, data were extracted from graphs using universal desktop ruler software (AVP Software Development, USA). When the group size was reported as a range (e.g., 6–7), the mean number of animals was used in our analysis (e.g. 6–7 = 6.5).

### 2.3 Quality assessment

We scored the risk of bias according to a five-item checklist derived from the consensus statement ‘Good laboratory practice’ in the modelling of stroke (Macleod et al., 2009): implementation in the experimental setup of any measure of randomization, any measure of blinding, prior sample size calculation, statement on animal welfare, and statement of a potential conflict of interest. For each of these items, a ‘yes’, a ‘NR’ (not reported), or a ‘no’ was scored. As a sixth item, we also scored whether the study was in accordance with the ARRIVE guidelines (Kilkenny et al., 2010).

### 2.4 Meta-analysis

Data were analyzed using the software Comprehensive Meta-Analysis (CMA, version 3.0). Different studies used different scales to measure the same outcome; thus, we calculated the Hedges’ g standardized mean difference (SMD) — the mean of the experimental group minus the mean of the control group divided by the pooled SDs of the two groups — instead of the raw mean difference.

In order to adequately represent weight of individual experiments in the *meta*-analysis, control groups were adjusted in case they served for more than one experimental group. In that case, the number of observations in that control group was divided by the number of experimental groups served.

Individual SMDs were subsequently pooled to obtain an overall SMD and 95% confidence interval. Since we did not expect one true underlying effect of all the *meta*-analyzable studies, we used the random effects model [14], which takes into account the precision of individual studies and the variation between studies and weighs each study accordingly.

Sources for heterogeneity were explored using I^2^ to describe the percentage of the variability in the effect estimates that is due to heterogeneity rather than sampling error (Higgins and Thompson, 2002). We expected the variance to be comparable within the subgroups (i.e., the pooled treatments); therefore, we assumed a common across-study variance across subgroups. No sub-subgroup analyses were calculated due to low number of experiments per therapeutic approach.

We used funnel plots, Trim and Fill analysis, and Egger regression to assess potential publication bias. SMDs may cause funnel plot distortion, thus, we plotted the SMD against $1/\sqrt{n}$, a sample size-based precision estimate (Zwetsloot et al., 2017).

## 3 Results

### 3.1 Study selection process

**Fig. 1:**
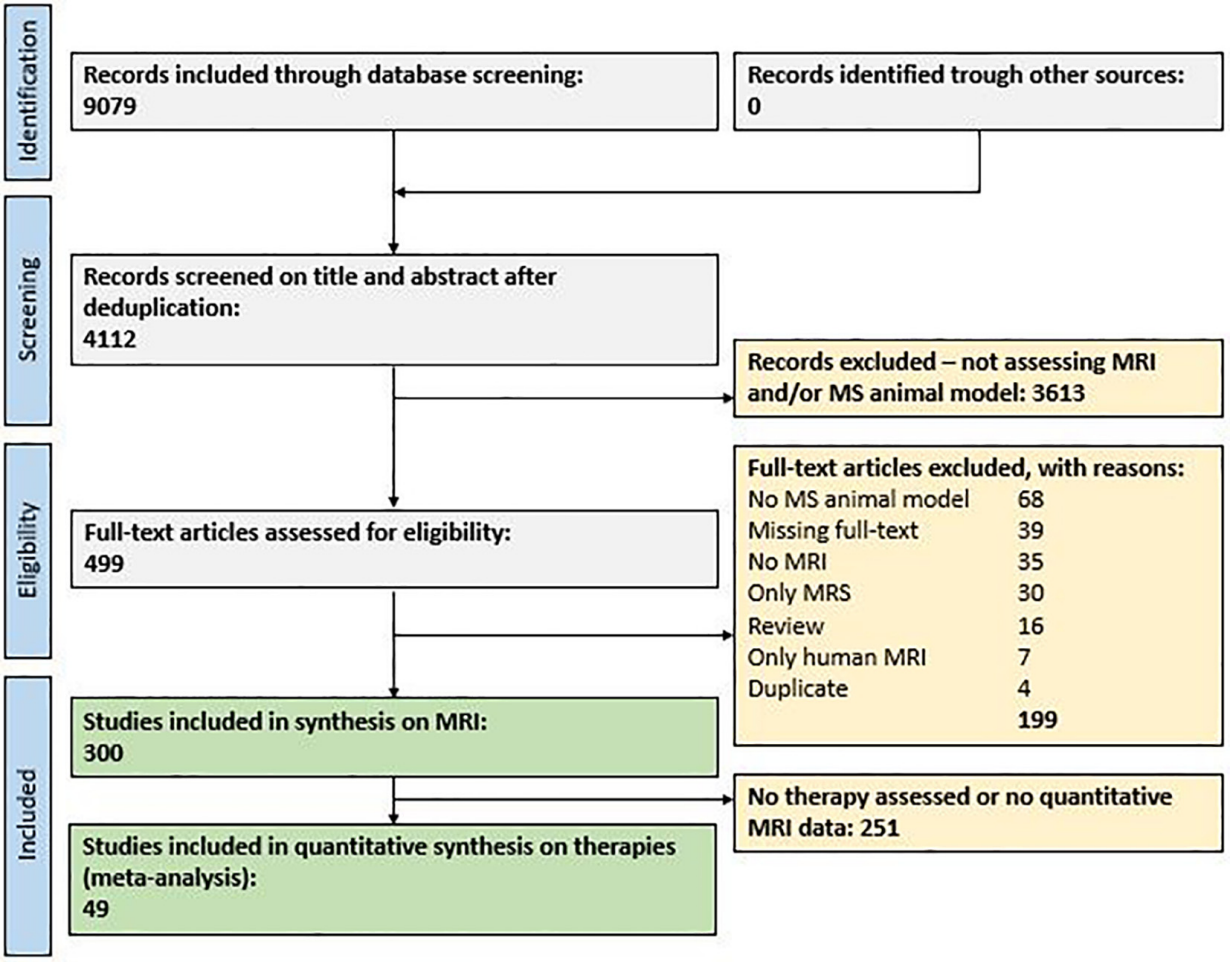
Prisma flow chart of the study selection process (Moher et al., 1999). Deduplication refers to removing identical studies found in multiple medical databases (e.g. same references in EMBASE and MEDLINE). Four duplicate studies were removed in the eligibility stage. Abbreviations: MRI, magnetic resonance imaging; MRS, magnetic resonance spectroscopy; MS, multiple sclerosis.

Fig. 1 depicts the flow chart of the study selection process (Moher et al., 1999). A search string for MS animal models and MRI was used in conjunction with an animal filter (de Vries et al., 2014). A total of 9079 publications were retrieved via EMBASE, Medline, PubMed, Scopus, and Web of Science, of which 4112 publications remained after deduplication. After initial screening of titles and abstracts, 499 publications were included in the full-text search. Of these, 300 unique publications met our inclusion criteria for the synthesis on experimental methods (Supplementary reference list). Of these, 67 unique publications investigated a potential MS therapy, whereas 49 unique publications contained quantitative structural MRI data and could therefore be used for the quantitative synthesis on therapy effect in MRI (*meta*-analysis). The remainder was excluded according to the criteria listed in Fig. 1.

The first report using MRI in an MS animal model was published in 1985 (Stewart et al., 1985). It showed that MRI lesions were apparent in primate brains prior to experimental autoimmune encephalomyelitis (EAE) symptom onset. The first report using an MS animal model to assess a therapy for its remyelinating potential *in vivo* and meeting our inclusion criteria, however, was only published in 1994 (Namer et al., 1994). Thus, all studies included in the *meta*-analysis were published between 1994 and 2020.

### 3.2 Description of the included studies

#### 3.2.1 Model organisms and disease models

**Fig. 2:**
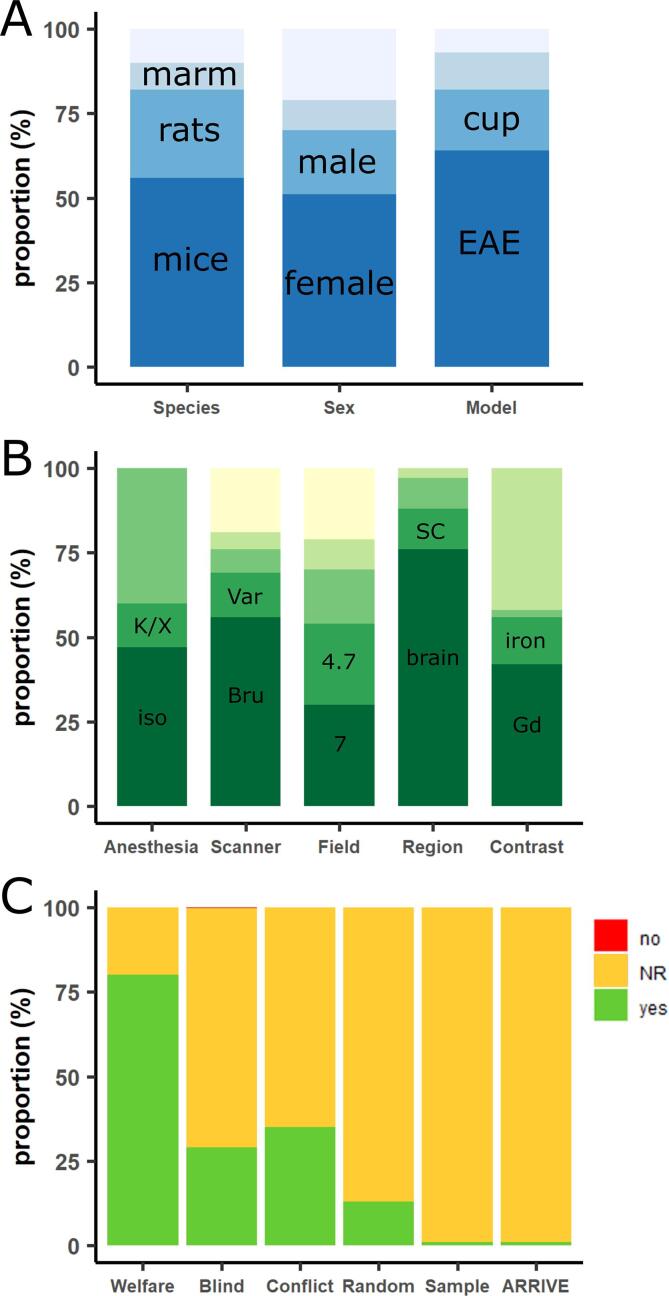
Bar plots demonstrating proportional study characteristics (A and B) and risk of bias assessment (C) of all 300 eligible studies. (A) Proportional study characteristics on species, animal sex, and multiple sclerosis animal model. (B) Proportional study characteristics on type of anesthesia for imaging, magnetic resonance imaging (MRI) scanner supplier, field strength of MRI scanner, scanned central nervous system region(s), and use of contrast agent. The top portion of the bar always represents the remaining pooled categories per characteristic or the proportion of studies who did not report on that particular study characteristic. (C) Risk of bias assessment of eligible studies using a six-item checklist (animal welfare reporting, blinding of experiments, statement of a potential conflict of interest, randomization in experimental setup, prior sample size calculation, study in accordance with ARRIVE guidelines (Kilkenny et al., 2010, Macleod et al., 2009)). For each of these items, ‘yes’, ‘NR’ (not reported), or ‘no’ was scored. Except for the item animal welfare statement, the majority of studies have unclear risk of bias (i.e., not reported; orange bar). Abbreviations: Bru, Bruker; Cup, cuprizone; EAE, experimental autoimmune encephalomyelitis; Gd, gadolinium; Iso, isoflurane; K/X, ketamine-xylazine; marm, marmosets; NR, not reported; Var, Varian; SC, spinal cord. (For interpretation of the references to colour in this figure legend, the reader is referred to the web version of this article.)

The characteristics of the 300 included publications are shown in Supplementary Table 1. Study characteristics are summarized in Fig. 2. Most of the publications used mice as an experimental model organism (167 publications, 56%, Fig. 2A), followed by rats (77, 26%). Marmosets (23, 8%), guinea pigs (15, 5%), macaques (13, 4%), dogs (2, <1%), mini pigs (1, <1%), rhesus monkeys (1, <1%) and swines (1, <1%) were less commonly used. Mice with C57Bl/6 background (114, 68%) were most commonly used, followed by SJL (35, 21%). Lewis was the most commonly used rat strain (44, 57%). The majority of publications used animals with female sex (152, 51%) as compared to male sex (57, 19%). Twenty-eight publications (9%) used both sexes, and 63 publications (21%) did not report the sex of their model organism. In many publications, no information on animal weight (197, 66%), age (97, 32%) or animal housing (172, 57%) was available. Fifty-six publications (19%) did not report the total number of animals they used.

A wide variety of animal models has been used to mimic MS pathology in the model organisms: 191 publications used EAE (64%, Fig. 2A), followed by cuprizone (54, 18%), Theiler’s murine encephalomyelitis (TMEV, 11, 4%), lysolecithin (9, 3%), targeted EAE (7, 2%), chronic hyponatremia (3, 1%), intracerebral cytokine injection (3, 1%), lipopolysaccharide (3, 1%), delayed type hypersensitivity (2, <1%), optic neuritis (upon EAE induction, 2, <1%), spontaneous Japanese macaque encephalomyelitis (2, <1%), ethidium bromide (2, <1%), and vector-based cytokine overexpression (1, <1%). Seven publications (2%) used two MS animal models. One publication (<1%) did not report which animal model was used.

#### 3.2.2 Assessed therapies

A total of 44 different therapies (47 different therapeutic approaches) were investigated in 49 of the publications. The assessed therapies are listed in Supplementary Table 2.

#### 3.2.3 Imaging parameters

The most common anesthesia for the imaging was isoflurane (141, 47%, Fig. 2B) in concentrations between 1 and 5 vol%, followed by ketamine/xylazine (40, 13%).

Regarding MRI scanner, 167 publications used a Bruker MRI system (56%, Fig. 2B), followed by Varian (38, 13%), General Electric (22, 7%), Siemens (15, 5%), Agilent (11, 4%), Philips (9, 3%) and Oxford Instruments (7, 2%). SMIS (3), Picker (3), Hitachi (1), SISCO (1), Rapid Biomedical (1), Technicare (1) and a scanner from the National High Magnetic Field Laboratory (NHMFL, 1) were less commonly used. Twenty publications (7%) did not report which MRI system they used. Most publications used a magnetic field strength of 7 T (T; 90, 30%), followed by 4.7 T (72, 24%), 9.4 T (47, 16%), 1.5 T (26, 9%), and 11.7 T (14, 5%). The lowest field strength was 0.15 T in two publications from 1985 and 1991 (Stewart et al., 1991, Stewart et al., 1985). The highest field strength was 21.1 T in a publication from 2019 (Waiczies et al., 2019). Relatively few publications reported on technical specifications about the used gradient system (77, 26%), magnet (103, 34%), or receiver coil (189, 63%).

Whereas *in vivo* imaging only was performed in 232 publications (77%, Fig. 2B), *ex vivo* imaging only was performed in 36 publications (12%). 32 publications (11%) acquired both *in vivo* and *ex vivo* MR images, respectively. Brain only was imaged by most publications (228, 76%) followed by spinal cord only (37, 12%). Both brain and spinal cord were imaged by 26 publications (9%). 86 publications acquired longitudinal neuroimaging (37% of *in vivo* imaging publications). The longest imaging follow-up time was 12 months.

A total of 709 MRI sequences were applied in all 300 publications. Commonly used sequences included fast or Turbo Spin Echo/Rapid Acquisition with Refocused Echoes (TSE/RARE) (146, 21%), conventional spin echo (101, 14%), Fast Low Angle Shot (FLASH) (62, 9%), and conventional gradient echo sequences (40, 6%). No sequence information was reported in 179 sequences (25%). For most sequences, both an echo time (TE) and a repetition time (TR) was reported (609, 86%). In contrast, there was a general lack of information on other basic imaging parameters: information on receiver bandwidth was reported for 21 sequences (3%), flip angle on 150 sequences (21%), field of view on 401 sequences (57%), and matrix size on 451 sequences (64%).

Out of 709 MRI sequences, mostly T2-weighted images were acquired (215, 30%), followed by T1-weighted (163, 23%), T2*-weighted (55, 8%), and proton density-weighted (30, 4%). Sixty-two publications acquired diffusion-weighted images (DWI, 21%), and 35 publications acquired magnetization transfer images (MTI, 12%).

Of 62 publications acquiring DWI, 27 reported the maximum b value (44%). B values ranged from 50 to 3000 s/mm^2^. Twenty-five publications reported the pulse duration (δ, 40%) and the time between the pulses (Δ, 40%). Only a few publications reported the number of directions (11 publications, 18%) or the diffusion gradient strength (9 publications, 15%). Of note, more than 1/3 of publications using DWI were released in the three years prior to our search cutoff date.

#### 3.2.4 Contrast agent

MRI scans were enhanced by various contrast agents in 126 publications (42%, Fig. 2B). Some publications used more than one contrast agent. The most commonly used contrast agent was gadolinium-DTPA (Gd-DTPA, Magnevist), which was used in 62 publications (21%), followed by Gd-DOTA (Dotarem; 13, 4%), gadodiamide (Omniscan; 4, 1%), and gadoteridol (Prohance; 4, 1%). Altogether, 93 publications (31%) used Gd-based contrast agents, followed by iron-based contrast agents (43, 14%), especially iron oxides such as SPIO or USPIO. Manganese- (5 publications) and magnesium-based (1 publication) contrast agents were less commonly used.

4 out of 126 respective publications (67%) reported on the concentration of Gd-based contrast agents. Most of these publications used concentrations between 0.2 and 0.5 mmol/kg (62, 49%).

#### 3.2.5 Study quality and risk of bias

Poor reporting in preclinical studies is a known issue, and therefore many items of commonly used risk of bias tools are scored as unclear risk of bias (Hooijmans et al., 2014). We therefore scored the risk of bias according to a five-item checklist derived from the consensus statement ‘Good laboratory practice’ in the modelling of stroke (Macleod et al., 2009). These items were also scored in a comparable study in EAE (Vesterinen et al., 2010) and in a study of toxic demyelination models (TD) (Hooijmans et al., 2019). Compliance with animal welfare regulations or an approved animal license were reported in 80% of cases (EAE: 32%, TD: 58%). Blinding of the experiment at any level was reported in 29% of publications (EAE: 16%, TD: 38%). Due to the experimental setup, one publication (<1%) was not able to blind their researchers, and this was explicitly reported. A statement about conflict of interest was reported in 35% of publications (EAE: 6%, TD: 38%). Thirteen percent of publications reported randomization at any level (EAE: 9%, TD: 5%). Four publications (1%) reported a prior sample size calculation (EAE: <1%, TD: 2%). These findings are summarized in Fig. 2C.

Finally, as a sixth item, we checked whether the publication was in accordance with the ARRIVE guidelines — an initiative to improve the reporting standard of animal research (Kilkenny et al., 2010). Three publications reported being in accordance with the ARRIVE guidelines (1%, Fig. 2C).

### 3.3 Meta-analysis

#### 3.3.1 Magnetic resonance imaging as outcome

The 49 publications in the *meta*-analysis included studies containing a total of 95 different experiments. Different MRI outcome measures were used to measure therapy efficacy. Out of 95 experiments, T2 or contrast-enhancing lesion load were most commonly used as MRI readout (26 experiments, 27% and 24 experiments, 25%, respectively), followed by DWI (20 experiments, 21%), MTI (7 experiments, 7%), T1 lesion load (4 experiments, 5%), and brain atrophy measure (5 experiments, 4%).

Pooling the individual effect sizes of all therapies in our *meta*-analyses showed that the therapies described in the literature had a beneficial effect on MRI outcomes (e.g. volume of T2 brain lesion load, standardized mean difference (SMD): 1.24, 95% CI: [1.06, 1.34], p = 0.021, Table 1). The overall heterogeneity between the studies was moderate (I^2^ = 37%).

**Table 1:** Summary of outcome parameters used in studies assessing therapeutic approaches in MS animal models. Not all studies reported the sex of the used animals. Abbreviations: Cup, cuprizone; EAE, experimental autoimmune encephalomyelitis; MRI, magnetic resonance imaging; SMD, standardized mean difference.).

Outcome	Therapeutic approaches tested	Model			Sex		Species			Overall SMD [95% CI] and p value	I^2^ (%)
		EAE	Cup	Other	F	M	Mice	Rats	Other		
MRI	47 (95 experiments)	66	17	12	63	21	42	28	25	1.24 [1.06, 1.34] p = 0.021	37
(Re-) myelination	25 (27 experiments)	14	8	5	17	7	11	8	8	1.72 [1.09, 2.30] p = 0.014	80
Neuroinflammation	18 (20 experiments)	13	4	3	9	5	7	6	7	1.20 [0.93, 1.55] p = 0.019	61
Neurodegeneration	5 (5 experiments)	2	3	0	3	0	5	0	0	0.81 [0.10, 1.51] p = 0.044	61

**Fig. 3:**
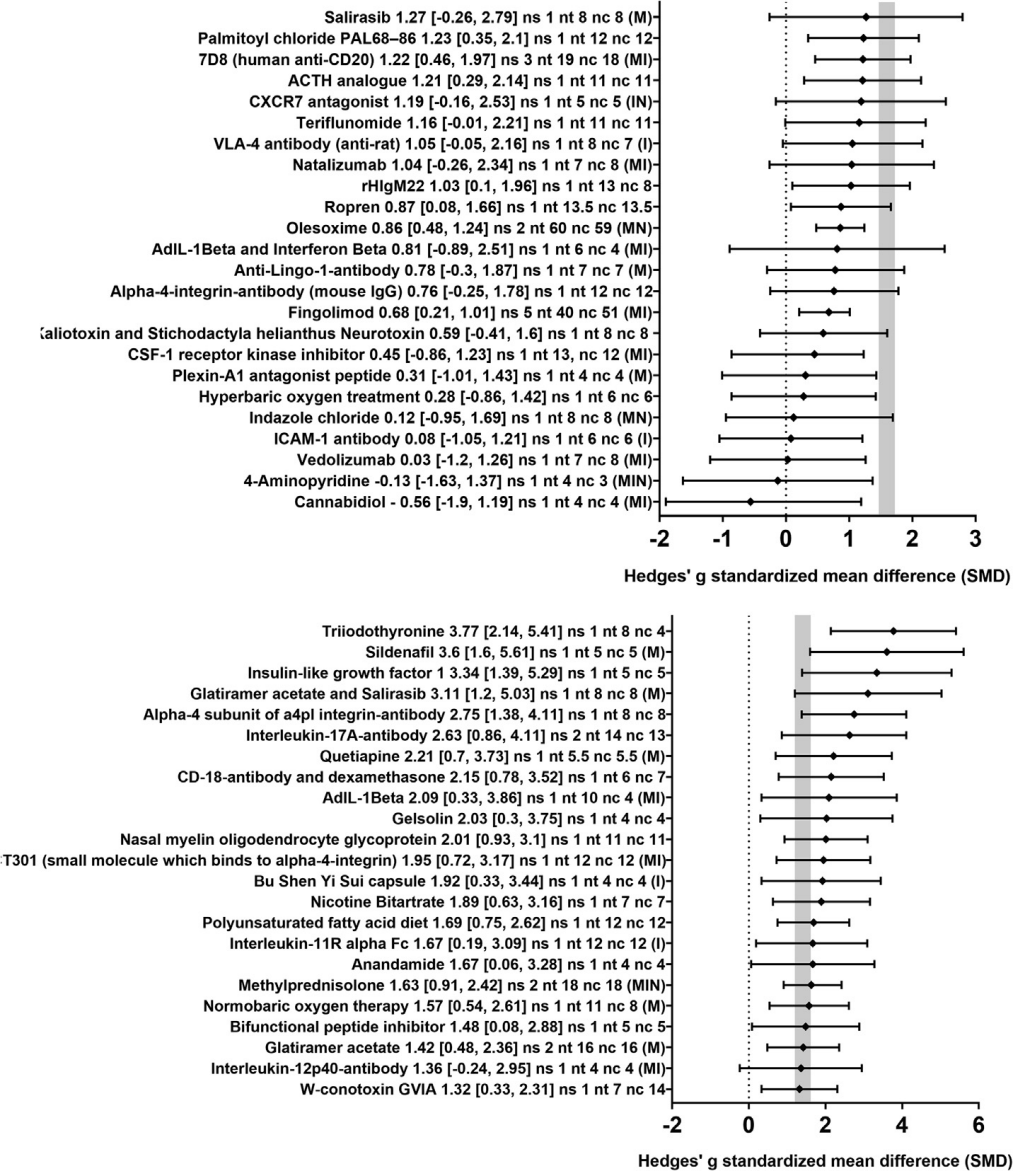
Forest plot of the included studies for MRI outcomes. The diamond indicates the global estimate and the whiskers its 95% confidence interval (CI). The numbers listed after each therapy are: the exact effect size with its 95% CI, the number of included studies for a certain intervention (ns), the total number of treated animals (nt) and control animals (nc). The capital letters in round brackets indicate whether the corresponding therapy has also been tested for (re-)myelination (M), inflammation (I) and/or neurodegeneration (N). The gray bar indicates the 95% CI of the overall effect size. The dotted line indicates an SMD of 0, i.e. studies with whiskers which overlap this dotted line do not show statistically significant SMDs between therapy and control group. Also consider Supplementary Figs. 1–3 for effect on (re-)myelination, inflammation and/or neurodegeneration. References are provided in the Supplementary information.

In order to obtain a more detailed overview of the efficacy of the various therapies included in this review, we also analyzed the effect of the 47 different therapeutic approaches for their impact on MRI outcomes separately. Twenty-eight therapeutic approaches led to a significant improvement of MRI outcomes (Fig. 3). For the remaining 19 therapeutic approaches, no statistically significant results were found. Of note, in most cases, only one study was available per therapeutic approach. The median sample size [interquartile range, IQR] was 7 [5–10] for the treatment groups and 6 [4–8] for the control groups.

#### 3.3.2 Histological markers of (re-)myelination as outcome

In total, 25 publications also assessed the remyelinating potential of therapeutic approaches. The most commonly used staining method to assess (re-)myelination was Luxol fast blue (LFB, 11 publications, 44%), followed by immunohistochemistry/-fluorescence stainings (for MBP, PLP, or Fluoromyelin; 8 publications, 32%) and electron microscopy (5 publications, 20%), and. Cyanin staining was used in one publication (4%).

Pooling the individual effect sizes of all therapies in our *meta*-analyses showed that the therapies described in the literature had a beneficial effect on histological outcomes of (re-)myelination (e.g. number of thinly myelinated axons in electron microscopy, SMD: 1.72, 95% CI: [1.09, 2.30], p = 0.014 Table 1). The overall heterogeneity (Higgins and Thompson, 2002) between the studies was high (I^2^ = 80%), however, reflecting the anticipated differences between interventions, models used, and study design.

In order to obtain a more detailed overview of the efficacy of the various therapies included in this *meta*-analysis, we also analyzed the effect of the 24 different therapeutic approaches for their impact on (re-)myelination histology markers separately (with the corresponding method to assess (re-)myelination in square brackets). Seven therapeutic approaches led to a significant improvement of histological outcomes of (re-)myelination (glatiramer acetate/salirasib [LFB], adenovirus expressing IL-1β [AdIL-1β] [MBP], triiodothyronine [electron microscopy], sildenafil [electron microscopy], salirasib [LFB], normobaric oxygen therapy [LFB], and olesoxime [electron microscopy]; Supplementary Fig. 1). For the remaining 17 therapeutic approaches, no statistically significant results were found. The median sample size [IQR] was 7 [5.38–9.5] for the treatment groups and 5.25 [3–7.25] for the control groups.

#### 3.3.3 Histological markers of neuroinflammation as outcome

In total, 17 publications also assessed the anti-inflammatory/immunomodulatory potential of therapeutic approaches. The most commonly used method to assess neuroinflammation was immunohistochemistry/-fluorescence (for CD3, ED1, Iba1, CD20, and/or Ox22; 10 publications, 59%). Hematoxylin and eosin staining was less commonly used (7 publications, 31%).

Pooling the individual effect sizes of all therapies in our *meta*-analyses showed that the therapies described in literature had a beneficial effect on histological outcomes of neuroinflammation (e.g. number of inflammatory CD3 + cells within parenchymal lesions, SMD: 1.20, 95% CI: [0.93, 1.55], p = 0.019 Table 1). The overall heterogeneity between the studies was substantial (I^2^ = 61%).

In order to obtain a more detailed overview of the efficacy of the various therapies included in this *meta*-analysis, we also analyzed the effect of the 18 different therapeutic approaches for their impact on inflammatory histology markers separately (with the corresponding method to assess inflammation in square brackets). Ten therapeutic approaches led to a significant improvement of histological outcomes of neuroinflammation (AdIL-1β [ED1], 7D8 [H&E], CXCR7 antagonist [CD3], ICAM-1 antibody [CD3], CSF-1 receptor kinase inhibitor [Iba1], CT301 [H&E], VLA-4 antibody [ED1], Interleukin-11R alpha Fc [CD4], IL anti-12p40 [H&E], and fingolimod [Ox22]; Supplementary Fig. 2). For the remaining 8 therapeutic approaches, no statistically significant results were found. The median sample size [IQR] was 6 [5–7.5] for the treatment groups and 5 [4–6] for the control groups.

#### 3.3.4 Histological markers of neurodegeneration as outcome

In total, 5 publications also assessed the neuroregenerative/-protective potential of therapeutic approaches. Immunohistochemistry/-fluorescence for neurofilament or SMI32 were used by 2 publications each.

Pooling the individual effect sizes of all therapies in our *meta*-analyses showed that the therapies described in the literature had a positive effect on histological outcomes of neurodegeneration (e.g. number of SMI-positive axons within neuro-inflammatory lesions, SMD: 0.81, 95% CI: [0.10, 1.51], p = 0.044, Table 1). The overall heterogeneity between the studies was substantial (I^2^ = 61%).

In order to obtain a more detailed overview of the efficacy of the various therapies included in this *meta*-analysis, we also analyzed the effect of the 5 different therapeutic approaches for their impact on neurodegeneration histology markers separately (with the corresponding method to assess inflammation in square brackets). Only one therapeutic approach (olesoxime [Neurofilament]) led to a significant improvement of histological outcomes of neurodegeneration (Supplementary Fig. 3). For the remaining 4 therapeutic approaches, no statistically significant results were found. The median sample size [IQR] was 4.5 [3.75 – 5.25] for the treatment groups and 4.5 [4–5.25] for the control groups.

#### 3.3.5 Correlation analysis

**Fig. 4:**
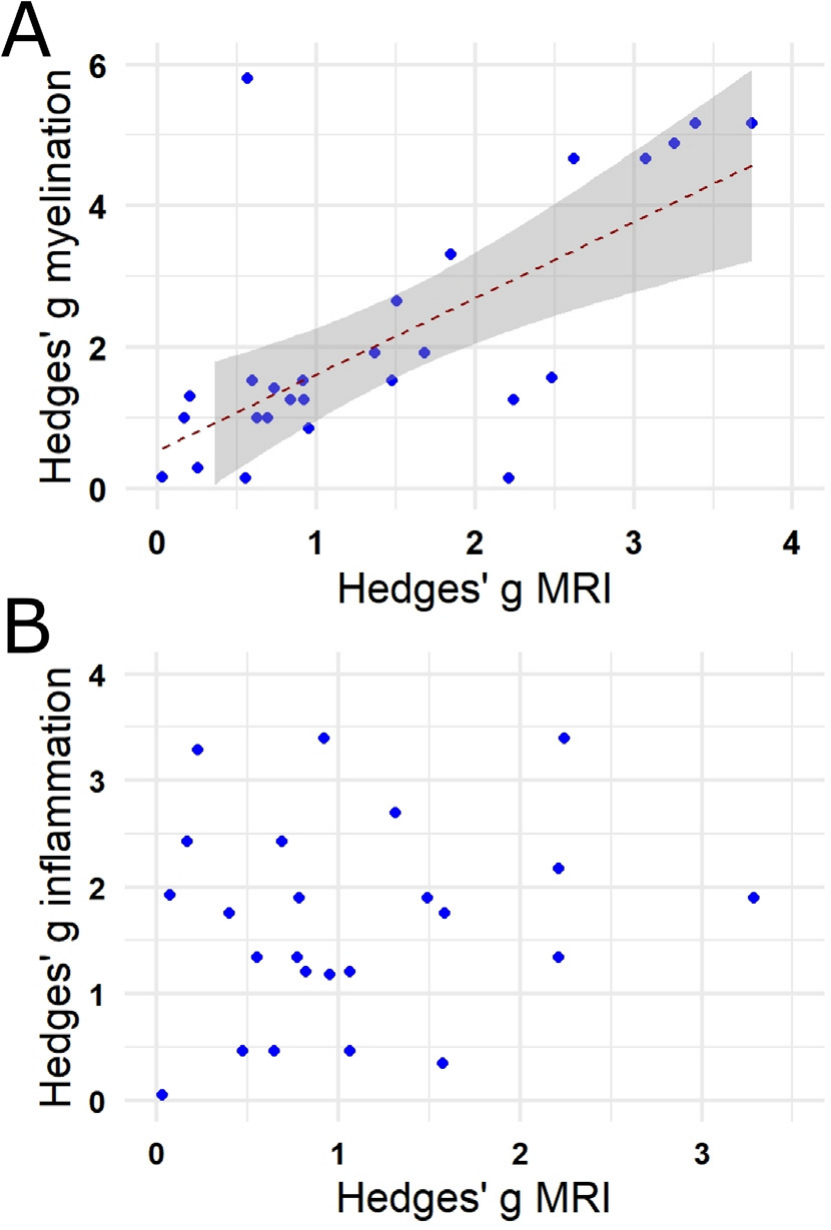
Correlation analysis between standardized mean difference (SMD) of the MRI outcomes and histological markers of (re-)myelination (A) or neuroinflammation (B). The analysis indicates a statistically significant correlation between SMDs of non-contrast-enhanced MRI outcomes and SMDs of (re-)myelination (r = 0.63, p < 0.001). No statistically significant correlation was found between SMDs of MRI outcomes and neuroinflammation.

We next asked how well MRI outcome measures correlate with histological markers of (re-)myelination or neuroinflammation. For this, we plotted the SMDs of the MRI outcomes against the SMDs of these histological outcomes. A positive correlation was found for any non-contrast-enhanced MRI outcomes (i.e. structural T1-weighted/T2-weighted and MTI measures as well as DWI measures) and measures of (re–)myelination (Fig. 4A). SMDs of MRI outcomes showed no correlation to neuroinflammation (Fig. 4B), Only 5 studies histologically assessed neurodegeneration. Hence, we did not assess correlation.

#### 3.3.6 Publication bias

**Fig. 5:**
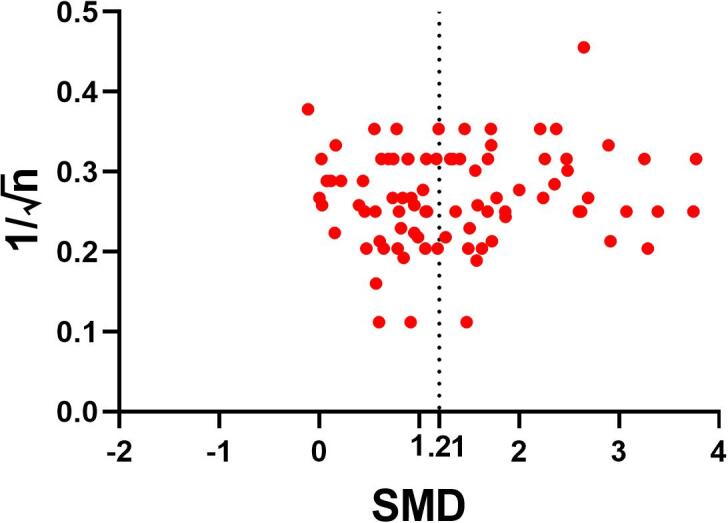
Evaluation of publication bias. Funnel plots for the outcome MRI indicating publication bias. The dashed line represents the standardized mean difference (SMD) of the summary effect.

In order to assess publication bias, we visually inspected the funnel plot and calculated Egger’s regression. The funnel plot is a graphical representation of trial size plotted against the reported effect size. An uneven scattering on both sides of the summary effect size indicates publication bias. Visual inspection of the funnel plot indicated the presence of publication bias for MRI data (Fig. 5). This finding was supported by Egger’s regression showing statistically significant evidence for small study effects (p = 0.001).

## 4 Discussion

MRI is widely used in preclinical research to investigate putative therapeutic approaches or pathogenic aspects in MS animal models. Tracking the large number of published studies in this field has proven difficult, however. In order to obtain an overview of these studies, we systematically reviewed methodological details of preclinical studies using MRI in MS animal models. Furthermore, a *meta*-analysis on therapeutic approaches provides evidence for a solid correlation between MRI outcomes measures and histological measures of (re-)myelination.

### 4.1 Risk of bias and reporting of methodological details

Accumulating evidence suggests low reproducibility rates in life sciences (Justice and Dhillon, 2016), including neuroscience (Steward and Balice-Gordon, 2014). A recent report indicates that, in the United States alone, the cumulative prevalence of irreproducible preclinical research exceeds 50% with an approximate cost of $28 billion/year (Freedman et al., 2015). An insufficient reporting of experimental details in (pre-)clinical research can contribute to this lack of reproducibility (Carp, 2012, Collins and Tabak, 2014). This problem is particularly apparent in MRI research, where small differences in imaging protocol can lead to large differences in tissue contrast (Amiri et al., 2018). Therefore, a detailed and accurate reporting of the used methodology and results is key for future reproducibility of findings from MRI studies and, more importantly, successful translation of preclinical findings to clinical trials. Reduction of methodologically inappropriate animal experiments could also ultimately reduce animal numbers.

Estimating the risk of bias in our included publications, by scoring whether measures to avoid bias were reported in six separate domains, suggests an overall high risk of bias of the included studies, albeit in the range of other published studies in the field (Hooijmans et al., 2019, Vesterinen et al., 2010). It has been shown that studies that report on measures to avoid bias, such as blinding of experimenters, yield substantially lower efficacy estimates (Macleod et al., 2008, Vesterinen et al., 2010). Thus, it is highly recommended to include such measures to the experimental design of any planned study.

Results from our systematic review show that an abundance of different species and MS animal models has been used in conjunction with MRI. Many studies have not reported on key methodological details of the experimental setup, e.g. 21% of all studies did not include information on the sex of used animals and 19% of all studies did not report the total number of studied animals. Guidelines for reporting experiments involving animals have been published to tackle this problem (Kilkenny et al., 2010, Landis et al., 2012); they are still insufficiently implemented to scientific practice, however (Baker et al., 2014) (only 3 out of 300 publications reported of being in accordance with the ARRIVE guidelines in our systematic review).

### 4.2 Minimal reporting guidelines

While the overall reporting on technical details regarding MRI system and image acquisition was reasonable, some important methodological details were seldom reported: many studies did not report on gradient system, receiver bandwidth, flip angle magnitude, field of view, matrix size or gradient strength, and number of directions in DWI. There was also a high variability on which technical aspects were reported and which were omitted. The poor reporting and the variability in reporting are due to a lack of reporting guidelines. Whereas such reporting guidelines for general aspects of preclinical animal research (Kilkenny et al., 2010) or clinical trials (Moher et al., 2001) have been proposed, no such guidelines are available for preclinical neuroimaging studies. Thus, based on the findings in this review and our experience with neuroimaging in animals, we propose minimal reporting guidelines (Table 2). The reporting suggestions are grouped according to experimental steps, i.e. details on the MRI system, details on animal anesthesia, details on sequence(s), details on contrast media (if applicable), and details on *ex vivo* imaging (if applicable). Even though we did not include other disease models to our analysis, these guidelines could be applied to any preclinical neuroscience research using MRI. We also request referees and journal editors to scrutinize papers for these details. A complete reporting of relevant information is key for a potential replication of findings (Kilkenny et al., 2010).

**Table 2:** Minimal reporting guidelines on technical MRI aspects.

1) MRI system	
• MRI system supplier (e.g. Bruker) • MR system model (e.g. AVANCE) • Field strength (e.g. 7 T)	• Gradient performance (e.g. 200 mT/m) • Coil (e.g. 8-channel phase array coil)

2) Animal anaesthesia	
Compound(s) (e.g. ketamine/xylazine)Concentration of compound(s) (e.g. 35 mg/kg)	Application form (e.g. intramuscular injection)
3) Pulse sequence(s)	
• Sequence (e.g. spin echo) • Purpose of sequence (e.g. measuring T1 lesion burden) • Weighting (e.g. T1-weighted) • Echo and repetition time (e.g. 3.5/2000 ms) • Inversion time (e.g. 900 ms, if applicable) • Flip angle magnitude (e.g. 15°) • Acquisition mode (e.g. 3D) • Acquisition plane (e.g. sagittal, in case of 2D imaging) • Multi-slice imaging (if applicable) • Number of echoes (e.g. 16, if applicable)	• Voxel size (e.g. 150 × 150 × 150 µm^3^ or 150 × 150 µm^2^ with slice thickness of 1 mm) • Matrix size (e.g. 256 × 256) • Field of view (e.g. 30 × 30 mm^2^) • Number of slices • Number of signal averages • Receiver bandwidth (e.g. 25.5 kHz) • Acquisition time for each sequence and total acquisition time • Fat saturation (e.g. chemical shift, if applicable)

For magnetization transfer imaging (MTI)	
• Saturation power (e.g. 0.9 µT) • Off-resonance pulse (e.g. 1 kHz) • Pulse shape (e.g. Gaussian shaped)	• Pulse length (e.g. 0.2 ms) • Number of pulses (e.g. 20) • MTR flip angle (e.g. 1045°)

For diffusion-weighted imaging (DWI)	
• Pulse gradient strength increment (G, e.g. 5 gauss/cm) • Diffusion gradient duration (δ, e.g. 10 ms) • Duration between paired gradients (Δ, e.g. 200 ms)	• b value (e.g. 1124 s/mm^2^) • Maximal q value (e.g. 500 cm^−1^) • Number of directions (e.g. 6)

4) Contrast agent	
• Contrast medium (e.g. gadoterate meglumine, including supplier) • Contrast-medium dose (e.g. 0.3 mmol/kg body weight)	• Application form (e.g. via tail vein catheter) • Exact time between imaging and application

5) *Ex vivo* imaging	
• Medium for animal perfusion (e.g. 4% formaldehyde) • Medium for immersion fixation (e.g. 4% formaldehyde) • Contrast agent for tissue immersion (e.g. gadoteridol)	• Time for immersion fixation (e.g. 24 h) • Medium during imaging (e.g. Fomblin)

### 4.3 Results from the *meta*-analyses

The *meta*-analysis for the MRI outcomes showed that therapies tested in MS animal models had an overall beneficial effect on the model disease course. The same is true for the outcomes (re-)myelination, neuroinflammation, and neurodegeneration. However, the effect-size summaries and the therapy effect sizes should be interpreted with caution, due to mostly small study sample sizes,differences in study design characteristics, and overall low numbers of studies. They should therefore not be used as rank order of potency.

Interestingly, there was a statistically significant positive correlation between SMDs from the non-contrast-enhanced MRI outcomes and histological measures of (re-)myelination. This suggests that non-contrast enhanced MRI outcomes reflect the underlying (re-)myelination status reasonably well. Surprisingly, despite the relative success of therapeutic development to modulate inflammation, our data did not support a significant correlation between MRI outcomes and histological outcomes of neuroinflammation. There were also too few studies available to do subgroup analysis for specific imaging outcomes such as contrast-enhancing lesions. More studies are thus needed to address whether there is a correlation between specific imaging findings and underlying histopathology in MS and/or corresponding animal models. This holds particularly true for measures of neurodegeneration: only four publications concomitantly assessed histological measures of neurodegeneration and MRI. Moreover, no therapies are currently approved to mitigate neurodegeneration in MS, which is present even early in the disease course (Filippi et al., 2018, Trapp et al., 1998). A deeper understanding about certain MR image features and their underlying histopathology could facilitate the choice for adequate outcomes in clinical trials (Maggi et al., 2014).

For the assessment of therapy efficacy, most publications used a T2 and/or contrast-enhancing lesion burden measure. These outcomes are also commonly used in the design of clinical trials and thus reflect sound outcome measures also for preclinical research (van Munster and Uitdehaag, 2017). It is noteworthy that DWI, including tractography, is increasingly used in preclinical neuroimaging research and is a popular choice for determining white matter microstructure *in vivo* (Jelescu and Budde, 2017). Hence, DWI has particular relevance for demyelinating and/or neurodegenerative pathology, both of which are hallmarks of MS (and to some degree MS animal models) (Lassmann and Bradl, 2016, Trapp et al., 1998). Recent attempts at standardizing DWI methodology in preclinical research further support its benefit in MS animal model neuroimaging (Anderson et al., 2020). Yet, the specificity of DWI findings needs to be further validated in correlative histopathology studies (Budde et al., 2009). Also, a careful choice of imaging parameters, such as gradient strength, gradient duration and diffusion time, is key for reliable DWI results (Jelescu and Budde, 2017).

Of note, only a few studies used MRI brain/spinal cord atrophy as outcome measure, even though this outcome is increasingly being used in clinical trials. A potential reason for this discrepancy is technical limitations during post-processing of images, which may impede the determination of a reliable atrophy rate in smaller-scale brains, such as from rodents, especially within the mostly brief time frame of animal studies (Kurniawan, 2018). However, a considerable number of studies performed longitudinal neuroimaging up to 12 months. Such longitudinal assessment of disease processes can greatly support pathophysiological understanding, particularly in neuroinflammation and therefore highly dynamic pathology (Maggi et al., 2017).

Finally, visual inspection of the funnel plot and testing of the Egger regression indicated publication bias, whereby effect sizes are overestimated. It has been suggested that publication bias may account for at least one-third of the efficacy reported in systematic reviews of animal stroke studies (Sena et al., 2010, Van der Worp et al., 2010). Similar overestimations of effect sizes are likely true for other model diseases, including MS.

## 5 Limitations

Our review has some limitations. (1) Many key methodological details of animal studies included in our review were poorly reported. Unfortunately, this also holds true for many other systematic reviews of animal studies (Hooijmans et al., 2015) — a situation that seriously hampers reliable risk of bias assessment. Although this limits our ability to reliably estimate the validity of the results of the included studies, we nevertheless included the poorly reported papers in this review because papers that do not report essential details are not necessarily methodologically impaired (Green and Higgins, 2005). (2) For the *meta*-analysis, the number of studies was low, while the variability between the studies was considerable. This influences the reliability of the conclusions drawn from this systematic review. To account for that, we anticipated heterogeneity by using a random rather than a fixed-effects model for the *meta*-analysis. (3) We did not perform post-hoc power-calculations due to their limited validity (Levine and Ensom, 2001). It is worth reiterating that sample sizes were small in most of the included studies, in line with previous findings from a large systematic review in neuroscience (Button et al., 2013). Small sample sizes imply low power, which lowers the likelihood that a statistically significant result reflects a true effect (Marino, 2017).

## 6 Conclusions

Our systematic review summarizes preclinical studies using MRI in MS animal models. We show that, whereas preclinically used MRI outcomes correlate well with underlying measures of (re-)myelination, reporting on certain technical aspects of MRI acquisition is poor. We therefore propose minimal, non-onerous reporting guidelines for studies using MRI in a preclinical setup. These guidelines address the important problem of insufficient methodological reporting and accompanying lack of experimental reproducibility. Taken together, findings from our study will inform preclinical researchers on adequate reporting of technical aspects of MRI acquisition. We hope this will encourage successful replication of future results and, eventually, successful bench-to-bedside translation of promising therapeutic approaches.

## Declaration of Competing Interest

The authors declare that they have no known competing financial interests or personal relationships that could have appeared to influence the work reported in this paper.
