# Supplementary material for: Magnetic resonance imaging in multiple sclerosis animal models: A systematic review, meta-analysis, and white paper
Source: Neuroimage Clin. 2020 Aug 2;28:102371. doi: 10.1016/j.nicl.2020.102371 (PMC7451445; doi:10.1016/j.nicl.2020.102371)
Supplement: Supplementary data 2 [file mmc2.docx]

**Supplementary reference list**

1 Abakumova, T. O. K. k., A. A.; Zharova, M. V.; Pozdeeva, D. A.; Gubskii, I. L.; Shepeleva, I. I.; Antonova, O. M.; Nukolova, N. V.; Kekelidze, Z. I.; Chekhonin, V. P. Cuprizone Model as a Tool for Preclinical Studies of the Efficacy of Multiple Sclerosis Diagnosis and Therapy. Bulletin of Experimental Biology and Medicine 159, 111-115 (2015).

2 Abramowski, P. K., S.; Ernst, T.; Lange, C.; Ittrich, H.; Schweizer, M.; Zander, A. R.; Martin, R.; Fehse, B. Mesenchymal Stromal/Stem Cells Do Not Ameliorate Experimental Autoimmune Encephalomyelitis and Are Not Detectable in the Central Nervous System of Transplanted Mice. Stem Cells Dev 25, 1134-1148, doi:http://dx.doi.org/10.1089/scd.2016.0020 (2016).

3 Absinta, M. et al. The "central vein sign" in inflammatory demyelination: The role of fibrillar collagen type I. Ann Neurol 85, 934-942 (2019).

4 Acs, P. K., M.; Norkute, A.; Johann, S.; Clarner, T.; Braun, A.; Berente, Z.; Komoly, S.; Beyer, C. 17beta-estradiol and progesterone prevent cuprizone provoked demyelination of corpus callosum in male mice. GLIA 57, 807-814, doi:http://dx.doi.org/10.1002/glia.20806 (2009).

5 Adler, S. M., J.; Williams, D. S.; Verbalis, J. G. Positive association between blood brain barrier disruption and osmotically-induced demyelination. Multiple Sclerosis 6, 24-31 (2000).

6 Adler, S. V., J. G.; Williams, D. Effect of rapid correction of hyponatremia on the blood-brain barrier of rats. Brain Research 679, 135-143 (1995).

7 Adler, S. V., J. G.; Meyers, S.; Simplaceanu, E.; Williams, D. S. Changes in cerebral blood flow and distribution associated with acute increases in plasma sodium and osmolality of chronic hyponatremic rats. Experimental Neurology 163, 63-71 (2000).

8 Aggarwal, M. J., M. V.; Calabresi, P. A.; Mori, S.; Zhang, J. Probing mouse brain microstructure using oscillating gradient diffusion MRI. Magnetic Resonance in Medicine 67, 98-109, doi:http://dx.doi.org/10.1002/mrm.22981 (2012).

9 Aharoni, R. S., E.; Blumenfeld-Katzir, T.; Eilam, R.; Sela, M.; Assaf, Y.; Arnon, R. Magnetic resonance imaging characterization of different experimental autoimmune encephalomyelitis models and the therapeutic effect of glatiramer acetate. Experimental Neurology 240, 130-144, doi:http://dx.doi.org/10.1016/j.expneurol.2012.11.004 (2013).

10 Ahrens, E. T. L., D. H.; Readhead, C.; Brosnan, C. F.; Fraser, S. E.; Jacobs, R. E. MR microscopy of transgenic mice that spontaneously acquire experimental allergic encephalomyelitis. Magnetic Resonance in Medicine 40, 119-132 (1998).

11 Aizman, E. M., A.; Chapman, J.; Assaf, Y.; Kloog, Y. The combined treatment of Copaxone and Salirasib attenuates experimental autoimmune encephalomyelitis (EAE) in mice. Journal of Neuroimmunology 229, 192-203, doi:http://dx.doi.org/10.1016/j.jneuroim.2010.08.022 (2010).

12 All, A. H. W., P.; Agrawal, G.; Gorelik, M.; Lee, C.; Thakor, N. V.; Bulte, J. W.; Kerr, D. A. Effect of MOG sensitization on somatosensory evoked potential in Lewis rats. Journal of the Neurological Sciences 284, 81-89, doi:http://dx.doi.org/10.1016/j.jns.2009.04.025 (2009).

13 Anderson, S. A. S.-Q., J.; Jordan, E. K.; Arbab, A. S.; Martin, R.; McFarland, H.; Frank, J. A. Magnetic resonance imaging of labeled T-cells in a mouse model of multiple sclerosis. Annals of Neurology 55, 654-659 (2004).

14 Anthony, D. C. S., N. R.; McAteer, M. A.; Davis, B.; Choudhury, R. P. Detection of brain pathology by magnetic resonance imaging of iron oxide micro-particles. Methods Mol Biol 686, 213-227, doi:http://dx.doi.org/10.1007/978-1-60761-938-3_9 (2011).

15 Anthony, D. C. S., N. R.; Losey, P.; Meier, D. P.; Leppert, D. Investigation of immune and CNS-mediated effects of fingolimod in the focal delayed-type hypersensitivity multiple sclerosis model. Neuropharmacology 79, 534-541, doi:http://dx.doi.org/10.1016/j.neuropharm.2013.12.022 (2014).

16 Atkinson, K. C. et al. Diffusion tensor imaging identifies aspects of therapeutic estrogen receptor β ligand-induced remyelination in a mouse model of multiple sclerosis. Neurobiol Dis 130, 104501 (2019).

17 Axthelm, M. K. B., D. N.; Marracci, G. H.; Su, W.; Mullaney, E. T.; Manoharan, M.; Kohama, S. G.; Pollaro, J.; Witkowski, E.; Wang, P.; Rooney, W. D.; Sherman, L. S.; Wong, S. W. Japanese macaque encephalomyelitis: a spontaneous multiple sclerosis-like disease in a nonhuman primate. Annals of Neurology 70, 362-373, doi:http://dx.doi.org/10.1002/ana.22449 (2011).

18 Badawi, A. H. K., P.; Wang, W. T.; Choi, I. Y.; Lee, P.; Vines, C. M.; Siahaan, T. J. Suppression of EAE and prevention of blood-brain barrier breakdown after vaccination with novel bifunctional peptide inhibitor. Neuropharmacology 62, 1874-1881, doi:http://dx.doi.org/10.1016/j.neuropharm.2011.12.013 (2012).

19 Baeten, K. A., P.; Hendriks, J.; Theunissen, E.; Gelan, J.; Hellings, N.; Stinissen, P. Tracking of myelin-reactive T cells in experimental autoimmune encephalomyelitis (EAE) animals using small particles of iron oxide and MRI. NMR in Biomedicine 23, 601-609, doi:http://dx.doi.org/10.1002/nbm.1501 (2010).

20 Baeten, K. H., J. J.; Hellings, N.; Theunissen, E.; Vanderlocht, J.; Ryck, L. D.; Gelan, J.; Stinissen, P.; Adriaensens, P. Visualisation of the kinetics of macrophage infiltration during experimental autoimmune encephalomyelitis by magnetic resonance imaging. Journal of Neuroimmunology 195, 1-6, doi:http://dx.doi.org/10.1016/j.jneuroim.2007.11.008 (2008).

21 Beckmann, N. et al. Brain region-specific enhancement of remyelination and prevention of demyelination by the CSF1R kinase inhibitor BLZ945. Acta Neuropathol Commun 6, 9 (2018).

22 Bell, J. C. L., Q.; Gan, Y.; Liu, Q.; Liu, Y.; Shi, F. D.; Turner, G. H. Visualization of inflammation and demyelination in 2D2 transgenic mice with rodent MRI. Journal of Neuroimmunology 264, 35-40, doi:http://dx.doi.org/10.1016/j.jneuroim.2013.09.008 (2013).

23 Belloli, S. et al. (18)F-VC701-PET and MRI in the in vivo neuroinflammation assessment of a mouse model of multiple sclerosis. J Neuroinflammation 15, 33 (2018).

24 Bendszus, M. L., G.; Jestaedt, L.; Misselwitz, B.; Solymosi, L.; Toyka, K.; Stoll, G. Gadofluorine M enhancement allows more sensitive detection of inflammatory CNS lesions than T2-w imaging: a quantitative MRI study. Brain 131, 2341-2352, doi:http://dx.doi.org/10.1093/brain/awn156 (2008).

25 Ben-Hur, T. v. H., R. B.; Einstein, O.; Aharonowiz, M.; Xue, R.; Frost, E. E.; Mori, S.; Reubinoff, B. E.; Bulte, J. W. Serial in vivo MR tracking of magnetically labeled neural spheres transplanted in chronic EAE mice. Magnetic Resonance in Medicine 57, 164-171 (2007).

26 Beraud, E. V., A.; Regaya, I.; Confort-Gouny, S.; Siaud, P.; Ibarrola, D.; Le Fur, Y.; Barbaria, J.; Pellissier, J. F.; Sabatier, J. M.; Medina, I.; Cozzone, P. J. Block of neural Kv1.1 potassium channels for neuroinflammatory disease therapy. Annals of Neurology 60, 586-596 (2006).

27 Berger, C. H., P.; Kindler-Baumann, D.; Rudin, M.; Rausch, M. Analysis of lesion development during acute inflammation and remission in a rat model of experimental autoimmune encephalomyelitis by visualization of macrophage infiltration, demyelination and blood-brain barrier damage. NMR in Biomedicine 19, 101-107 (2006).

28 Binamé, F. et al. Disruption of Sema3A/Plexin-A1 inhibitory signalling in oligodendrocytes as a therapeutic strategy to promote remyelination. EMBO Mol Med 11, e10378 (2019).

29 Biton, I. E. M., A.; Kidron, D.; Assaf, Y.; Cohen, Y. Improved detectability of experimental allergic encephalomyelitis in excised swine spinal cords by high b-value q-space DWI. Experimental Neurology 195, 437-446 (2005).

30 Bittner, S. M., S. G.; Göbel, K.; Melzer, N.; Herrmann, A. M.; Simon, O. J.; Weishaupt, A.; Budde, T.; Bayliss, D. A.; Bendszus, M.; Wiendl, H. TASK1 modulates inflammation and neurodegeneration in autoimmune inflammation of the central nervous system. Brain 132, 2501-2516 (2009).

31 Blair, T. C. M., M.; Rawlings-Rhea, S. D.; Tagge, I.; Kohama, S. G.; Hollister-Smith, J.; Ferguson, B.; Woltjer, R. L.; Frederick, M. C.; Pollaro, J.; Rooney, W. D.; Sherman, L. S.; Bourdette, D. N.; Wong, S. W. Immunopathology of Japanese macaque encephalomyelitis is similar to multiple sclerosis. Journal of Neuroimmunology 291, 1-10, doi:http://dx.doi.org/10.1016/j.jneuroim.2015.11.026 (2016).

32 Blezer, E. L. B., J.; Brok, H. P.; Nicolay, K.; t Hart, B. A. Quantitative MRI-pathology correlations of brain white matter lesions developing in a non-human primate model of multiple sclerosis. NMR in Biomedicine 20, 90-103 (2007).

33 Blezer, E. L. D., L. H.; Kooij, G.; Drexhage, J.; van der Pol, S. M.; Reijerkerk, A.; Dijkhuizen, R. M.; de Vries, H. E. In vivo MR imaging of intercellular adhesion molecule-1 expression in an animal model of multiple sclerosis. Contrast Media Mol Imaging 10, 111-121, doi:http://dx.doi.org/10.1002/cmmi.1602 (2015).

34 Bölcskei, K. et al. Behavioural alterations and morphological changes are attenuated by the lack of TRPA1 receptors in the cuprizone-induced demyelination model in mice. Journal of neuroimmunology 320, 1-10 (2018).

35 Boretius, S. E., A.; Dallenga, T.; Wrzos, C.; Tammer, R.; Bruck, W.; Nessler, S.; Frahm, J.; Stadelmann, C. Assessment of lesion pathology in a new animal model of MS by multiparametric MRI and DTI. Neuroimage 59, 2678-2688, doi:http://dx.doi.org/10.1016/j.neuroimage.2011.08.051 (2012).

36 Boretius, S. G., I.; Demmer, I.; Bahr, M.; Diem, R.; Michaelis, T.; Frahm, J. MRI of optic neuritis in a rat model. Neuroimage 41, 323-334, doi:http://dx.doi.org/10.1016/j.neuroimage.2008.02.021 (2008).

37 Boretius, S. S., B.; Watanabe, T.; Merkler, D.; Tammer, R.; Czeh, B.; Michaelis, T.; Frahm, J.; Fuchs, E. Monitoring of EAE onset and progression in the common marmoset monkey by sequential high-resolution 3D MRI. NMR in Biomedicine 19, 41-49 (2006).

38 Brochet, B. D., V. Pathological correlates of magnetization transfer imaging abnormalities in animal models and humans with multiple sclerosis. Neurology 53, S12-17 (1999).

39 Brochet, B. D., M. S.; Touil, T.; Anne, O.; Caille, J. M.; Dousset, V.; Petry, K. G. Early macrophage MRI of inflammatory lesions predicts lesion severity and disease development in relapsing EAE. Neuroimage 32, 266-274 (2006).

40 Brok, H. P. M. V. M., M.; Blezer, E.; Schantz, A.; Peritt, D.; Treacy, G.; Laman, J. D.; Bauer, J.; T Hart, B. A. Prevention of experimental autoimmune encephalomyelitis in common marmosets using an anti-IL-12p40 monoclonal antibody. Journal of Immunology 169, 6554-6563 (2002).

41 Broom, K. A. A., D. C.; Blamire, A. M.; Waters, S.; Styles, P.; Perry, V. H.; Sibson, N. R. MRI reveals that early changes in cerebral blood volume precede blood-brain barrier breakdown and overt pathology in MS-like lesions in rat brain. J Cereb Blood Flow Metab 25, 204-216 (2005).

42 Budde, M. D. K., J. H.; Liang, H. F.; Russell, J. H.; Cross, A. H.; Song, S. K. Axonal injury detected by in vivo diffusion tensor imaging correlates with neurological disability in a mouse model of multiple sclerosis. NMR in Biomedicine 21, 589-597 (2008).

43 Budde, M. D. X., M.; Cross, A. H.; Song, S. K. Axial diffusivity is the primary correlate of axonal injury in the experimental autoimmune encephalomyelitis spinal cord: a quantitative pixelwise analysis. Journal of Neuroscience 29, 2805-2813, doi:http://dx.doi.org/10.1523/JNEUROSCI.4605-08.2009 (2009).

44 Bulte, J. W. B.-H., T.; Miller, B. R.; Mizrachi-Kol, R.; Einstein, O.; Reinhartz, E.; Zywicke, H. A.; Douglas, T.; Frank, J. A. MR microscopy of magnetically labeled neurospheres transplanted into the Lewis EAE rat brain. Magnetic Resonance in Medicine 50, 201-205 (2003).

45 Cahill, L. S. et al. Aged hind-limb clasping experimental autoimmune encephalomyelitis models aspects of the neurodegenerative process seen in multiple sclerosis. Proc Natl Acad Sci U S A 116, 22710-22720 (2019).

46 Cao, J. et al. In Vivo Optical Imaging of Myelination Events in a Myelin Basic Protein Promoter-Driven Luciferase Transgenic Mouse Model. ASN Neuro 10, 1759091418777329 (2018).

47 Carambia, A. F., B.; Schwinge, D.; Bruns, O. T.; Salmen, S. C.; Ittrich, H.; Reimer, R.; Heine, M.; Huber, S.; Waurisch, C.; Eychmüller, A.; Wraith, D. C.; Korn, T.; Nielsen, P.; Weller, H.; Schramm, C.; Lüth, S.; Lohse, A. W.; Heeren, J.; Herkel, J. Nanoparticle-based autoantigen delivery to Treg-inducing liver sinusoidal endothelial cells enables control of autoimmunity in mice. Journal of Hepatology 62, 1349-1356 (2015).

48 Cate, H. S. W., Q. Z.; Kemper, D.; Merlo, D.; Wang, H. X.; Fang, K.; Egan, G. F.; Kilpatrick, T. J. Influence of methylprednisolone on magnetic resonance and histological measures during cuprizone-induced demyelination. Neuroscience Letters 483, 47-52, doi:http://dx.doi.org/10.1016/j.neulet.2010.07.060 (2010).

49 Chandran, P. U., J.; Markosyan, S.; Lisowski, A.; Buck, W.; Chin, C. L.; Fox, G.; Luo, F.; Day, M. Magnetic resonance imaging and histological evidence for the blockade of cuprizone-induced demyelination in C57BL/6 mice. Neuroscience 202, 446-453, doi:http://dx.doi.org/10.1016/j.neuroscience.2011.10.051 (2012).

50 Chen, C. C. Z., A.; Hsu, Y. H.; Chen, H. W.; Yang, L. C.; Chang, C. Neuroaxonal ion dyshomeostasis of the normal-appearing corpus callosum in experimental autoimmune encephalomyelitis. Experimental Neurology 210, 322-330, doi:http://dx.doi.org/10.1016/j.expneurol.2007.11.008 (2008).

51 Chen, J. W. B., M. O.; Aikawa, E.; Chiang, G.; Weissleder, R. Myeloperoxidase-targeted imaging of active inflammatory lesions in murine experimental autoimmune encephalomyelitis. Brain 131, 1123-1133, doi:http://dx.doi.org/10.1093/brain/awn004 (2008).

52 Chen, X. H., X.; Zou, Y.; Pi, R.; Liu, M.; Wang, T.; Zheng, X.; Liu, M.; Lin, M.; Liu, P.; Tao, L. Combined treatment with minocycline and prednisone attenuates experimental autoimmune encephalomyelitis in C57 BL/6 mice.[Erratum appears in J Neuroimmunol. 2009 Oct 30;215(1-2):130]. Journal of Neuroimmunology 210, 22-29, doi:http://dx.doi.org/10.1016/j.jneuroim.2009.02.016 (2009).

53 Chen, X. P., R.; Zou, Y.; Liu, M.; Ma, X.; Jiang, Y.; Mao, X.; Hu, X. Attenuation of experimental autoimmune encephalomyelitis in C57 BL/6 mice by osthole, a natural coumarin. Eur J Pharmacol 629, 40-46, doi:http://dx.doi.org/10.1016/j.ejphar.2009.12.008 (2010).

54 Chen, Z. C., J. T.; Johnson, M.; Gossman, Z. C.; Hendrickson, M.; Sakaie, K.; Martinez-Rubio, C.; Gale, J. T.; Trapp, B. D. Cuprizone does not induce CNS demyelination in nonhuman primates. Ann 2, 208-213, doi:http://dx.doi.org/10.1002/acn3.159 (2015).

55 Chin, C. L. P., M.; Bousquet, P. F.; Schwartz, A. J.; O'Connor, E. M.; Nelson, C. M.; Hradil, V. P.; Cox, B. F.; McRae, B. L.; Fox, G. B. Distinct spatiotemporal pattern of CNS lesions revealed by USPIO-enhanced MRI in MOG-induced EAE rats implicates the involvement of spino-olivocerebellar pathways. Journal of Neuroimmunology 211, 49-55, doi:http://dx.doi.org/10.1016/j.jneuroim.2009.03.012 (2009).

56 Chu, T. et al. Dynamic response of microglia/macrophage polarization following demyelination in mice. J Neuroinflammation 16, 188 (2019).

57 Chuhutin, A. et al. Diffusion Kurtosis Imaging maps neural damage in the EAE model of multiple sclerosis. Neuroimage 208, 116406 (2020).

58 Cisneros-Mejorado, A. J. et al. Demyelination-Remyelination of the Rat Caudal Cerebellar Peduncle Evaluated with Magnetic Resonance Imaging. Neuroscience (2019).

59 Collongues, N. C., J. B.; Blanc, F.; Steibel, J.; Lam, C. D.; Shabbir, A.; Trifilieff, E.; Honnorat, J.; Pham-Dinh, D.; Ghandour, M. S.; de Seze, J. The Brown Norway opticospinal model of demyelination: does it mimic multiple sclerosis or neuromyelitis optica? Int J Dev Neurosci 30, 487-497, doi:http://dx.doi.org/10.1016/j.ijdevneu.2012.05.004 (2012).

60 Cook, L. L. F., P. J.; Mitchell, J. R.; Karlik, S. J. In vivo 4.0-T magnetic resonance investigation of spinal cord inflammation, demyelination, and axonal damage in chronic-progressive experimental allergic encephalomyelitis. Journal of Magnetic Resonance Imaging 20, 563-571 (2004).

61 Cook, L. L. F., P. J.; Karlik, S. J. Pathology-guided MR analysis of acute and chronic experimental allergic encephalomyelitis spinal cord lesions at 1.5T. Journal of Magnetic Resonance Imaging 22, 180-188 (2005).

62 Crombe, A. et al. Deciphering the microstructure of hippocampal subfields with in vivo DTI and NODDI: Applications to experimental multiple sclerosis. Neuroimage 172, 357-368 (2018).

63 Cruz-Orengo, L. C., Y. J.; Kim, J. H.; Dorsey, D.; Song, S. K.; Klein, R. S. CXCR7 antagonism prevents axonal injury during experimental autoimmune encephalomyelitis as revealed by in vivo axial diffusivity. Journal of Neuroinflammation, 170 (2011).

64 de Santana Nunes, A. K. R., C.; de Oliveira, W. H.; Thomé, R.; Verinaud, L.; Tovar-Moll, F.; Peixoto, C. A. Phosphodiesterase-5 inhibition promotes remyelination by MCP-1/CCR-2 and MMP-9 regulation in a cuprizone-induced demyelination model. Experimental Neurology 275, 143-153 (2016).

65 DeBoy, C. A. Z., J.; Dike, S.; Shats, I.; Jones, M.; Reich, D. S.; Mori, S.; Nguyen, T.; Rothstein, B.; Miller, R. H.; Griffin, J. T.; Kerr, D. A.; Calabresi, P. A. High resolution diffusion tensor imaging of axonal damage in focal inflammatory and demyelinating lesions in rat spinal cord. Brain 130, 2199-2210 (2007).

66 Degaonkar, M. N. J., R.; Jagannathan, N. R. Sequential diffusion-weighted magnetic resonance imaging study of lysophosphatidyl choline-induced experimental demyelinating lesion: an animal model of multiple sclerosis. Journal of Magnetic Resonance Imaging 16, 153-159 (2002).

67 Degaonkar, M. N. R., P.; Jayasundar, R.; Jagannathan, N. R. Determination of relaxation characteristics during preacute stage of lysophosphatidyl choline-induced demyelinating lesion in rat brain: an animal model of multiple sclerosis. Magn Reson Imaging 23, 69-73 (2005).

68 Deloire, M. S. T., T.; Brochet, B.; Dousset, V.; Caille, J. M.; Petry, K. G. Macrophage brain infiltration in experimental autoimmune encephalomyelitis is not completely compromised by suppressed T-cell invasion: in vivo magnetic resonance imaging illustration in effective anti-VLA-4 antibody treatment. Multiple Sclerosis 10, 540-548 (2004).

69 Deloire-Grassin, M. S. B., B.; Quesson, B.; Delalande, C.; Dousset, V.; Canioni, P.; Petry, K. G. In vivo evaluation of remyelination in rat brain by magnetization transfer imaging. Journal of the Neurological Sciences 178, 10-16 (2000).

70 Derdelinckx, J. et al. Clinical and immunological control of experimental autoimmune encephalomyelitis by tolerogenic dendritic cells loaded with MOG-encoding mRNA. J Neuroinflammation 16, 167 (2019).

71 Desai, R. A. D., A. L.; Tachrount, M.; Kasti, M.; Laulund, F.; Golay, X.; Smith, K. J. Cause and prevention of demyelination in a model multiple sclerosis lesion. Annals of Neurology 79, 591-604 (2016).

72 Diem, R. D., I.; Boretius, S.; Merkler, D.; Schmelting, B.; Williams, S. K.; Sattler, M. B.; Bahr, M.; Michaelis, T.; Frahm, J.; Bruck, W.; Fuchs, E. Autoimmune optic neuritis in the common marmoset monkey: comparison of visual evoked potentials with MRI and histopathology. Invest Ophthalmol Vis Sci 49, 3707-3714, doi:http://dx.doi.org/10.1167/iovs.08-1896 (2008).

73 Dommisse, R. L., D.; Spanoghe, M.; Van Audekerke, J.; Van der Linden, A.; Gravenmade, H.; Van de Vyver, F. in Proceedings of the 13th Annual International Conference of the IEEE Engineering in Medicine and Biology Society.pt 1 edn 79-80 (Publ by IEEE).

74 Dousset, V. B., B.; Vital, A.; Gross, C.; Benazzouz, A.; Boullerne, A.; Bidabe, A. M.; Gin, A. M.; Caille, J. M. Lysolecithin-induced demyelination in primates: preliminary in vivo study with MR and magnetization transfer. AJNR Am J Neuroradiol 16, 225-231 (1995).

75 Dousset, V. D., C.; Ballarino, L.; Quesson, B.; Seilhan, D.; Coussemacq, M.; Thiaudiere, E.; Brochet, B.; Canioni, P.; Caille, J. M. In vivo macrophage activity imaging in the central nervous system detected by magnetic resonance. Magnetic Resonance in Medicine 41, 329-333 (1999).

76 Duckers, H. J. M., H. J.; Verhaagen, J.; Nicolay, K.; Gispen, W. H. Longitudinal in vivo magnetic resonance imaging studies in experimental allergic encephalomyelitis: effect of a neurotrophic treatment on cortical lesion development. Neuroscience 77, 1163-1173 (1997).

77 Elo, P. et al. Folate receptor-targeted positron emission tomography of experimental autoimmune encephalomyelitis in rats. J Neuroinflammation 16, 252 (2019).

78 Elo, P. et al. Vascular adhesion protein-1 is actively involved in the development of inflammatory lesions in rat models of multiple sclerosis. J Neuroinflammation 15, 128 (2018).

79 Engberink, R. D. v. d. P., S. M.; Walczak, P.; van der Toorn, A.; Viergever, M. A.; Dijkstra, C. D.; Bulte, J. W.; de Vries, H. E.; Blezer, E. L. Magnetic resonance imaging of monocytes labeled with ultrasmall superparamagnetic particles of iron oxide using magnetoelectroporation in an animal model of multiple sclerosis. Mol Imaging 9, 268-277 (2010).

80 Esposito, G. D. A., L.; Bartoli, A.; Chaabane, L.; Terreno, E. Evaluation of the co-registration capabilities of a MRI/PET compatible bed in an Experimental autoimmune encephalomyelitis (EAE) model. Nucl. Instrum. Methods Phys. Res. Sect. A-Accel. Spectrom. Dect. Assoc. Equip. 702, 108-110, doi:10.1016/j.nima.2012.08.102 (2013).

81 Falangola, M. F. G., D. N.; Tabesh, A.; Hui, E. S.; Nie, X.; Jensen, J. H.; Gerum, S. V.; Hu, C.; Lafrancois, J.; Collins, H. R.; Helpern, J. A. Histological correlation of diffusional kurtosis and white matter modeling metrics in cuprizone-induced corpus callosum demyelination. NMR in Biomedicine 27, 948-957 (2014).

82 Fang, M. H., D.; Zhang, F.; Hu, Z.; Yang, J.; Jiang, H.; Han, S. Antineuroinflammatory and neurotrophic effects of CNTF and C16 peptide in an acute experimental autoimmune encephalomyelitis rat models. Frontiers in Neuroanatomy 7 (2013).

83 Fjær, S. B., L.; Lundervold, A.; Myhr, K. M.; Pavlin, T.; Torkildsen, Ø; Wergeland, S. Deep gray matter demyelination detected by magnetization transfer ratio in the cuprizone model. PLoS ONE 8 (2013).

84 Fjaer, S. B., L.; Myhr, K. M.; Torkildsen, O.; Wergeland, S. Magnetization transfer ratio does not correlate to myelin content in the brain in the MOG-EAE mouse model. Neurochemistry International 83-84, 28-40, doi:http://dx.doi.org/10.1016/j.neuint.2015.02.006 (2015).

85 Floris, S. B., E. L.; Schreibelt, G.; Dopp, E.; van der Pol, S. M.; Schadee-Eestermans, I. L.; Nicolay, K.; Dijkstra, C. D.; de Vries, H. E. Blood-brain barrier permeability and monocyte infiltration in experimental allergic encephalomyelitis: a quantitative MRI study. Brain 127, 616-627 (2004).

86 Ford, C. C. C., T. L.; Karp, J.; Herndon, R. M. Magnetic resonance imaging of experimental demyelinating lesions. Magnetic Resonance in Medicine 14, 461-481 (1990).

87 Fournier, A. P. et al. Reduced spinal cord parenchymal cerebrospinal fluid circulation in experimental autoimmune encephalomyelitis. J Cereb Blood Flow Metab 39, 1258-1265 (2019).

88 Fournier, A. P. et al. Prediction of disease activity in models of multiple sclerosis by molecular magnetic resonance imaging of P-selectin. Proc Natl Acad Sci U S A 114, 6116-6121 (2017).

89 Gadjanski, I. B., S.; Williams, S. K.; Lingor, P.; Knoferle, J.; Sattler, M. B.; Fairless, R.; Hochmeister, S.; Suhs, K. W.; Michaelis, T.; Frahm, J.; Storch, M. K.; Bahr, M.; Diem, R. Role of n-type voltage-dependent calcium channels in autoimmune optic neuritis. Annals of Neurology 66, 81-93, doi:http://dx.doi.org/10.1002/ana.21668 (2009).

90 Gaitan, M. I. M., P.; Wohler, J.; Leibovitch, E.; Sati, P.; Calandri, I. L.; Merkle, H.; Massacesi, L.; Silva, A. C.; Jacobson, S.; Reich, D. S. Perivenular brain lesions in a primate multiple sclerosis model at 7-tesla magnetic resonance imaging. Multiple Sclerosis 20, 64-71, doi:http://dx.doi.org/10.1177/1352458513492244 (2014).

91 Gareau, P. J. R., B. K.; Karlik, S. J.; Mitchell, J. R. Magnetization transfer and multicomponent T2 relaxation measurements with histopathologic correlation in an experimental model of MS. Journal of Magnetic Resonance Imaging 11, 586-595 (2000).

92 Gareau, P. J. W., A. C.; Cofer, G. P.; Johnson, G. A. Imaging inflammation: direct visualization of perivascular cuffing in EAE by magnetic resonance microscopy. Journal of Magnetic Resonance Imaging 16, 28-36 (2002).

93 Gilli, F. C., X.; Pachner, A. R.; Gimi, B. High-Resolution Diffusion Tensor Spinal Cord MRI Measures as Biomarkers of Disability Progression in a Rodent Model of Progressive Multiple Sclerosis. PLoS ONE 11, e0160071, doi:http://dx.doi.org/10.1371/journal.pone.0160071 (2016).

94 Gobel, K. W., J. H.; Herrmann, A. M.; Wachsmuth, L.; Pankratz, S.; Bittner, S.; Budde, T.; Kleinschnitz, C.; Faber, C.; Wiendl, H.; Meuth, S. G. 4-Aminopyridine ameliorates mobility but not disease course in an animal model of multiple sclerosis. Experimental Neurology 248, 62-71, doi:http://dx.doi.org/10.1016/j.expneurol.2013.05.016 (2013).

95 González-García, C. et al. Mechanisms of action of cannabidiol in adoptively transferred experimental autoimmune encephalomyelitis. Exp Neurol 298, 57-67 (2017).

96 Grossman, R. I. L., R. P.; Macchi, P. J.; Joseph, P. M. MR of acute experimental allergic encephalomyelitis. AJNR Am J Neuroradiol 8, 1045-1048 (1987).

97 Guglielmetti, C. et al. Longitudinal evaluation of demyelinated lesions in a multiple sclerosis model using ultrashort echo time magnetization transfer (UTE-MT) imaging. Neuroimage 208, 116415 (2020).

98 Guglielmetti, C. et al. Hyperpolarized (13)C MR metabolic imaging can detect neuroinflammation in vivo in a multiple sclerosis murine model. Proc Natl Acad Sci U S A 114, E6982-e6991 (2017).

99 Guglielmetti, C. L. B., D.; Santermans, E.; Salas-Perdomo, A.; Daans, J.; De Vocht, N.; Shah, D.; Hoornaert, C.; Praet, J.; Peerlings, J.; Kara, F.; Bigot, C.; Mai, Z.; Goossens, H.; Hens, N.; Hendrix, S.; Verhoye, M.; Planas, A. M.; Berneman, Z.; van der Linden, A.; Ponsaerts, P. Interleukin-13 immune gene therapy prevents CNS inflammation and demyelination via alternative activation of microglia and macrophages. GLIA 64, 2181-2200, doi:http://dx.doi.org/10.1002/glia.23053 (2016).

100 Guglielmetti, C. V., J.; Roelant, E.; Mai, Z.; Daans, J.; Van Audekerke, J.; Naeyaert, M.; Vanhoutte, G.; Delgado, Y. Palacios R.; Praet, J.; Fieremans, E.; Ponsaerts, P.; Sijbers, J.; Van der Linden, A.; Verhoye, M. Diffusion kurtosis imaging probes cortical alterations and white matter pathology following cuprizone induced demyelination and spontaneous remyelination. Neuroimage 125, 363-377, doi:http://dx.doi.org/10.1016/j.neuroimage.2015.10.052 (2016).

101 Guy, J. M., S.; Fitzsimmons, J.; Beck, B.; Rao, N. A. Disruption of the blood-brain barrier in experimental optic neuritis: immunocytochemical co-localization of H2O2 and extravasated serum albumin. Invest Ophthalmol Vis Sci 35, 1114-1123 (1994).

102 Guy, J. R. S., P.; Leibovitch, E.; Jacobson, S.; Silva, A. C.; Reich, D. S. Custom fit 3D-printed brain holders for comparison of histology with MRI in marmosets. J Neurosci Methods 257, 55-63, doi:10.1016/j.jneumeth.2015.09.002 (2016).

103 Haanstra, K. G. H., S. O.; Estêvão, D. M. L.; Blezer, E. L. A.; Bauer, J.; Yang, L. L.; Wyant, T.; Csizmadia, V.; T Hart, B. A.; Fedykx, E. R. Antagonizing the α4β1 integrin, but not α4β7, inhibits leukocytic infiltration of the central nervous system in rhesus monkey experimental autoimmune encephalomyelitis. Journal of Immunology 190, 1961-1973 (2013).

104 Hamilton, A. M. et al. Central nervous system targeted autoimmunity causes regional atrophy: a 9.4T MRI study of the EAE mouse model of Multiple Sclerosis. Sci Rep 9, 8488 (2019).

105 Hao, J. S., A. R.; Turner, G. H.; Wu, J.; Whiteaker, P.; Lukas, R. J.; Shi, F. D. Attenuation of CNS inflammatory responses by nicotine involves alpha7 and non-alpha7 nicotinic receptors. Experimental Neurology 227, 110-119, doi:http://dx.doi.org/10.1016/j.expneurol.2010.09.020 (2011).

106 Harsan, L. A. S., J.; Zaremba, A.; Agin, A.; Sapin, R.; Poulet, P.; Guignard, B.; Parizel, N.; Grucker, D.; Boehm, N.; Miller, R. H.; Ghandour, M. S. Recovery from chronic demyelination by thyroid hormone therapy: myelinogenesis induction and assessment by diffusion tensor magnetic resonance imaging. Journal of Neuroscience 28, 14189-14201, doi:http://dx.doi.org/10.1523/JNEUROSCI.4453-08.2008 (2008).

107 Hart, B. A. B., J.; Muller, H. J.; Melchers, B.; Nicolay, K.; Brok, H.; Bontrop, R. E.; Lassmann, H.; Massacesi, L. Histopathological characterization of magnetic resonance imaging-detectable brain white matter lesions in a primate model of multiple sclerosis: a correlative study in the experimental autoimmune encephalomyelitis model in common marmosets (Callithrix jacchus). American Journal of Pathology 153, 649-663 (1998).

108 Hawkins, C. P. M., P. M.; MacKenzie, F.; Kesselring, J.; Tofts, P. S.; du Boulay, E. P.; Landon, D. N.; McDonald, W. I. Duration and selectivity of blood-brain barrier breakdown in chronic relapsing experimental allergic encephalomyelitis studied by gadolinium-DTPA and protein markers. Brain 113, 365-378 (1990).

109 Hawkins, C. P. M., P. M.; Landon, D. N.; McDonald, W. I. Metabolically dependent blood-brain barrier breakdown in chronic relapsing experimental allergic encephalomyelitis. Acta Neuropathol (Berl) 83, 630-635 (1992).

110 Heckl, S. N., T.; Herrmann, M.; Gartner, S.; Klose, U.; Schick, F.; Weissert, R.; Kuker, W. Experimental autoimmune encephalomyelitis (EAE): lesion visualization on a 3 Tesla clinical whole-body system after intraperitoneal contrast injection. ROFO Fortschr Geb Rontgenstr Nuklearmed 176, 1549-1554 (2004).

111 Heide, A. C. R., T. L.; Alvord, E. C., Jr.; Peterson, J.; Rose, L. M. Diffusion imaging of experimental allergic encephalomyelitis. Magnetic Resonance in Medicine 29, 478-484 (1993).

112 Helms, G. G.-R., E.; Schlumbohm, C.; Konig, J.; Dechent, P.; Fuchs, E.; Wilke, M. Structural and quantitative neuroimaging of the common marmoset monkey using a clinical MRI system. J Neurosci Methods 215, 121-131, doi:http://dx.doi.org/10.1016/j.jneumeth.2013.02.011 (2013).

113 Herrera, S. L. P., V. L.; Whittaker, H.; Smith, B. C.; Kim, A.; Schellenberg, A. E.; Thiessen, J. D.; Buist, R.; Del Bigio, M. R.; Martin, M. Damage to the optic chiasm in myelin oligodendrocyte glycoprotein-experimental autoimmune encephalomyelitis mice. Magn 7, 23-31, doi:http://dx.doi.org/10.4137/MRI.S19750 (2014).

114 Hoffmann, D. B. W., S. K.; Bojcevski, J.; Muller, A.; Stadelmann, C.; Naidoo, V.; Bahr, B. A.; Diem, R.; Fairless, R. Calcium influx and calpain activation mediate preclinical retinal neurodegeneration in autoimmune optic neuritis. J Neuropathol Exp Neurol 72, 745-757, doi:http://dx.doi.org/10.1097/NEN.0b013e31829c7370 (2013).

115 Hübner, N. S. et al. The connectomics of brain demyelination: Functional and structural patterns in the cuprizone mouse model. Neuroimage 146, 1-18 (2017).

116 Hunger, M. B., E.; Zhong, K.; Angenstein, F. Visualization of acute focal lesions in rats with experimental autoimmune encephalomyelitis by magnetic nanoparticles, comparing different MRI sequences including phase imaging. Journal of Magnetic Resonance Imaging 39, 1126-1135, doi:http://dx.doi.org/10.1002/jmri.24280 (2014).

117 Jagessar, S. A. H., N.; Bauer, J.; Blezer, E. L. A.; Laman, J. D.; Hellings, N.; T Hart, B. A. B-Cell depletion abrogates T cell-mediated demyelination in an antibody-nondependent common marmoset experimental autoimmune encephalomyelitis model. Journal of Neuropathology and Experimental Neurology 71, 716-728 (2012).

118 Jagessar, S. A. K., Y. S.; Heijmans, N.; van Driel, N.; van Straalen, L.; Bajramovic, J. J.; Brok, H. P.; Blezer, E. L.; Bauer, J.; Laman, J. D.; t Hart, B. A. Induction of progressive demyelinating autoimmune encephalomyelitis in common marmoset monkeys using MOG34-56 peptide in incomplete freund adjuvant. J Neuropathol Exp Neurol 69, 372-385, doi:http://dx.doi.org/10.1097/NEN.0b013e3181d5d053 (2010).

119 Jagessar, S. A. S., P. A.; Blezer, E.; Delarasse, C.; Pham-Dinh, D.; Laman, J. D.; Bauer, J.; Amor, S.; t Hart, B. Autoimmunity against myelin oligodendrocyte glycoprotein is dispensable for the initiation although essential for the progression of chronic encephalomyelitis in common marmosets. J Neuropathol Exp Neurol 67, 326-340, doi:http://dx.doi.org/10.1097/NEN.0b013e31816a6851 (2008).

120 Jaini, R. P., D. C.; Flask, C. A.; Macklin, W. B.; Tuohy, V. K. Myelin antigen load influences antigen presentation and severity of central nervous system autoimmunity. Journal of Neuroimmunology 259, 37-46, doi:http://dx.doi.org/10.1016/j.jneuroim.2013.03.012 (2013).

121 Janve, V. A. Z., Z.; Yao, S. Y.; Li, K.; Zhang, F. L.; Wilson, K. J.; Ou, X.; Does, M. D.; Subramaniam, S.; Gochberg, D. F. The radial diffusivity and magnetization transfer pool size ratio are sensitive markers for demyelination in a rat model of type III multiple sclerosis (MS) lesions. Neuroimage 74, 298-305, doi:http://dx.doi.org/10.1016/j.neuroimage.2013.02.034 (2013).

122 Jelescu, I. O. Z., M.; Winters, K. V.; Veraart, J.; Rajaratnam, A.; Kim, N. S.; Babb, J. S.; Shepherd, T. M.; Novikov, D. S.; Kim, S. G.; Fieremans, E. In vivo quantification of demyelination and recovery using compartment-specific diffusion MRI metrics validated by electron microscopy. Neuroimage 132, 104-114, doi:http://dx.doi.org/10.1016/j.neuroimage.2016.02.004 (2016).

123 Jiang, Y. Z., Y.; Chen, S.; Zhu, C.; Wu, A.; Liu, Y.; Ma, L.; Zhu, D.; Ma, X.; Liu, M.; Kang, Z.; Pi, R.; Peng, F.; Wang, Q.; Chen, X. The anti-inflammatory effect of donepezil on experimental autoimmune encephalomyelitis in C57 BL/6 mice. Neuropharmacology 73, 415-424, doi:http://dx.doi.org/10.1016/j.neuropharm.2013.06.023 (2013).

124 Johnson, H. L. W., R. C.; Jin, F.; Manhart, W. A.; LaFrance, S. J.; Pirko, I.; Johnson, A. J. Perforin competent CD8 T cells are sufficient to cause immune-mediated blood-brain barrier disruption. PLoS ONE 9, e111401, doi:http://dx.doi.org/10.1371/journal.pone.0111401 (2014).

125 Jordan, E. K. M., H. I.; Lewis, B. K.; Tresser, N.; Gates, M. A.; Johnson, M.; Lenardo, M.; Matis, L. A.; McFarland, H. F.; Frank, J. A. Serial MR imaging of experimental autoimmune encephalomyelitis induced by human white matter or by chimeric myelin-basic and proteolipid protein in the common marmoset. AJNR Am J Neuroradiol 20, 965-976 (1999).

126 Kap, Y. S. B., J.; Driel, Nv; Bleeker, W. K.; Parren, P. W.; Kooi, E. J.; Geurts, J. J.; Laman, J. D.; Craigen, J. L.; Blezer, E.; t Hart, B. A. B-cell depletion attenuates white and gray matter pathology in marmoset experimental autoimmune encephalomyelitis. J Neuropathol Exp Neurol 70, 992-1005, doi:http://dx.doi.org/10.1097/NEN.0b013e318234d421 (2011).

127 Kap, Y. S. V. D., N.; Blezer, E.; Parren, P. W. H. I.; Bleeker, W. K.; Laman, J. D.; Craigen, J. L.; T Hart, B. A. Late B cell depletion with a human anti-human CD20 IgG1κ monoclonal antibody halts the development of experimental autoimmune encephalomyelitis in marmosets. Journal of Immunology 185, 3990-4003 (2010).

128 Karlik, S. J. G., J. J.; Wong, C.; Vandervoort, M. K.; Noseworthy, J. H. NMR studies in experimental allergic encephalomyelitis: factors which contribute to T1 and T2 values. Magnetic Resonance in Medicine 14, 1-11 (1990).

129 Karlik, S. J. G., E. A.; Lee, D.; Noseworthy, J. H. Gadolinium enhancement in acute and chronic-progressive experimental allergic encephalomyelitis in the guinea pig. Magnetic Resonance in Medicine 30, 326-331 (1993).

130 Karlik, S. J. M., D.; St Louis, J.; Strejan, G. Correlation between MRI and clinico-pathological manifestations in Lewis rats protected from experimental allergic encephalomyelitis by acylated synthetic peptide of myelin basic protein. Magn Reson Imaging 17, 731-737 (1999).

131 Karlik, S. J. S., G.; Gilbert, J. J.; Noseworthy, J. H. NMR studies in experimental allergic encephalomyelitis (EAE): normalization of T1 and T2 with parenchymal cellular infiltration. Neurology 36, 1112-1114 (1986).

132 Kent, S. J. K., S. J.; Rice, G. P.; Horner, H. C. A monoclonal antibody to alpha 4-integrin reverses the MR-detectable signs of experimental allergic encephalomyelitis in the guinea pig. Journal of Magnetic Resonance Imaging 5, 535-540 (1995).

133 Khodanovich, M. G., V.; Pan, E.; Akulov, A.; Krutenkova, E.; Trusov, V.; Yarnykh, V.; Iop,. in 2nd International Conference and Young Scientist School ''Magnetic Resonance Imaging in Biomedical Research'' Vol. 677 Journal of Physics Conference Series (Iop Publishing Ltd, 2016).

134 Khodanovich, M. Y. et al. Histological validation of fast macromolecular proton fraction mapping as a quantitative myelin imaging method in the cuprizone demyelination model. Sci Rep 7, 46686 (2017).

135 Kim, H. W., P.; Kerr, C.; Galpoththawela, C.; Gilad, A. A.; Muja, N.; Bulte, J. W. Immunomodulation by transplanted human embryonic stem cell-derived oligodendroglial progenitors in experimental autoimmune encephalomyelitis. Stem Cells 30, 2820-2829, doi:http://dx.doi.org/10.1002/stem.1218 (2012).

136 Kim, H. W., P.; Muja, N.; Campanelli, J. T.; Bulte, J. W. ICV-transplanted human glial precursor cells are short-lived yet exert immunomodulatory effects in mice with EAE. GLIA 60, 1117-1129, doi:http://dx.doi.org/10.1002/glia.22339 (2012).

137 Kim, J. H. B., M. D.; Liang, H. F.; Klein, R. S.; Russell, J. H.; Cross, A. H.; Song, S. K. Detecting axon damage in spinal cord from a mouse model of multiple sclerosis. Neurobiology of Disease 21, 626-632 (2006).

138 Kirschbaum, K. S., J. K.; Zeller, M. W.; Deumelandt, K.; Bode, J.; Sharma, R.; Krüwel, T.; Fischer, M.; Hoffmann, A.; Da Silva, M. C.; Muckenthaler, M. U.; Wick, W.; Tews, B.; Chen, J. W.; Heiland, S.; Bendszus, M.; Platten, M.; Breckwoldt, M. O. In vivo nanoparticle imaging of innate immune cells can serve as a marker of disease severity in a model of multiple sclerosis. Proceedings of the National Academy of Sciences of the United States of America 113, 13227-13232 (2016).

139 Krauspe, B. M. D., W.; Beyer, C.; Baumgartner, W.; Denecke, B.; Janssen, K.; Langhans, C. D.; Clarner, T.; Kipp, M. Short-term cuprizone feeding verifies N-acetylaspartate quantification as a marker of neurodegeneration. Journal of Molecular Neuroscience 55, 733-748, doi:http://dx.doi.org/10.1007/s12031-014-0412-6 (2015).

140 Kriszta, G. et al. Investigation of Cuprizone-Induced Demyelination in mGFAP-Driven Conditional Transient Receptor Potential Ankyrin 1 (TRPA1) Receptor Knockout Mice. Cells 9 (2019).

141 Kuharik, M. A. E., M. K.; Farlow, M. R.; Becker, G. J.; Azzarelli, B.; Klatte, E. C.; Augustyn, G. T.; Dreesen, R. G. Gd-enhanced MR imaging of acute and chronic experimental demyelinating lesions. AJNR Am J Neuroradiol 9, 643-648 (1988).

142 Ladewig, G. J., L.; Misselwitz, B.; Solymosi, L.; Toyka, K.; Bendszus, M.; Stoll, G. Spatial diversity of blood-brain barrier alteration and macrophage invasion in experimental autoimmune encephalomyelitis: a comparative MRI study. Experimental Neurology 220, 207-211, doi:http://dx.doi.org/10.1016/j.expneurol.2009.08.027 (2009).

143 Laman, J. D. t. H., B. A.; Brok, H.; Meurs, Mv; Schellekens, M. M.; Kasran, A.; Boon, L.; Bauer, J.; Boer, Md; Ceuppens, J. Protection of marmoset monkeys against EAE by treatment with a murine antibody blocking CD40 (mu5D12). European Journal of Immunology 32, 2218-2228 (2002).

144 Le Blon, D. G., C.; Hoornaert, C.; Quarta, A.; Daans, J.; Dooley, D.; Lemmens, E.; Praet, J.; De Vocht, N.; Reekmans, K.; Santermans, E.; Hens, N.; Goossens, H.; Verhoye, M.; Van der Linden, A.; Berneman, Z.; Hendrix, S.; Ponsaerts, P. Intracerebral transplantation of interleukin 13-producing mesenchymal stem cells limits microgliosis, oligodendrocyte loss and demyelination in the cuprizone mouse model. Journal of Neuroinflammation 13, 288 (2016).

145 Lee, J. S., K.; Kang, B. T.; Yao, B.; Fukunaga, M.; van Gelderen, P.; Palumbo, S.; Bosetti, F.; Silva, A. C.; Duyn, J. H. The contribution of myelin to magnetic susceptibility-weighted contrasts in high-field MRI of the brain. Neuroimage 59, 3967-3975, doi:http://dx.doi.org/10.1016/j.neuroimage.2011.10.076 (2012).

146 Lee, N. J. et al. Spatiotemporal distribution of fibrinogen in marmoset and human inflammatory demyelination. Brain 141, 1637-1649 (2018).

147 Lefeuvre, J. A. et al. The spectrum of spinal cord lesions in a primate model of multiple sclerosis. Mult Scler 26, 284-293 (2020).

148 Levy Barazany, H. B., D.; Puckett, L.; Blanga-Kanfi, S.; Borenstein-Auerbach, N.; Yang, K.; Peron, J. P.; Weiner, H. L.; Frenkel, D. Brain MRI of nasal MOG therapeutic effect in relapsing-progressive EAE. Experimental Neurology 255, 63-70, doi:http://dx.doi.org/10.1016/j.expneurol.2014.02.010 (2014).

149 Levy, H. A., Y.; Frenkel, D. Characterization of brain lesions in a mouse model of progressive multiple sclerosis. Experimental Neurology 226, 148-158, doi:http://dx.doi.org/10.1016/j.expneurol.2010.08.017 (2010).

150 Li, A. et al. Myeloperoxidase Molecular MRI Reveals Synergistic Combination Therapy in Murine Experimental Autoimmune Neuroinflammation. Radiology 293, 158-165 (2019).

151 Li, Y. Z., Y.; Han, W.; Hu, F.; Qian, Y.; Chen, Q. TRO19622 promotes myelin repair in a rat model of demyelination. International Journal of Neuroscience 123, 810-822, doi:http://dx.doi.org/10.3109/00207454.2013.804523 (2013).

152 Li-ChunHsieh, K. S., S.; Zeller, M. W. G.; Pulli, B.; Ali, M.; Wang, C. H.; Chiou, T. T. Y.; Tsang, Y. M.; Lee, P. S.; Stossel, T. P.; Chen, J. W. Gelsolin decreases actin toxicity and inflammation in murine multiple sclerosis. Journal of Neuroimmunology 287, 36-42, doi:10.1016/j.jneuroim.2015.08.006 (2015).

153 Lin, T. H. et al. Diffusion MRI quantifies early axonal loss in the presence of nerve swelling. J Neuroinflammation 14, 78 (2017).

154 Lin, T. H. K., J. H.; Perez-Torres, C.; Chiang, C. W.; Trinkaus, K.; Cross, A. H.; Song, S. K. Axonal transport rate decreased at the onset of optic neuritis in EAE mice. Neuroimage 100, 244-253, doi:http://dx.doi.org/10.1016/j.neuroimage.2014.06.009 (2014).

155 Lin, T. H. S., W. M.; Chiang, C. W.; Trinkaus, K.; Cross, A. H.; Song, S. K. Diffusion fMRI detects white-matter dysfunction in mice with acute optic neuritis. Neurobiology of Disease 67, 1-8, doi:http://dx.doi.org/10.1016/j.nbd.2014.02.007 (2014).

156 Linker, R. A. K., A.; Horn, T.; Gold, R.; Maurer, M.; Bendszus, M. Iron particle-enhanced visualization of inflammatory central nervous system lesions by high resolution: preliminary data in an animal model. AJNR Am J Neuroradiol 27, 1225-1229 (2006).

157 Linker, R. A. R., M.; Bendszus, M.; Ladewig, G.; Briel, A.; Schirner, M.; Maurer, M.; Hauff, P. In vivo molecular imaging of adhesion molecules in experimental autoimmune encephalomyelitis (EAE). J Autoimmun 25, 199-205 (2005).

158 Liu, T., Chen, Y., Thomas, A. M. & Song, X. CEST MRI with distribution-based analysis for assessment of early stage disease activity in a mouse model of multiple sclerosis: An initial study. NMR in biomedicine 32, e4139 (2019).

159 Lodygensky, G. A. W., T.; Stump, M.; Holtzman, D. M.; Inder, T. E.; Neil, J. J. In vivo MRI analysis of an inflammatory injury in the developing brain. Brain Behav Immun 24, 759-767, doi:http://dx.doi.org/10.1016/j.bbi.2009.11.005 (2010).

160 Luchetti, A. M., D.; Ruffini, F.; Galli, R.; Falini, A.; Quattrini, A.; Scotti, G.; Comi, G.; Martino, G.; Furlan, R.; Politi, L. S. Monoclonal antibodies conjugated with superparamagnetic iron oxide particles allow magnetic resonance imaging detection of lymphocytes in the mouse brain. Mol Imaging 11, 114-125 (2012).

161 Luo, T. et al. Characterizing Structural Changes With Devolving Remyelination Following Experimental Demyelination Using High Angular Resolution Diffusion MRI and Texture Analysis. J Magn Reson Imaging 49, 1750-1759 (2019).

162 MacKenzie-Graham, A. R., G. A.; Avedisian, A.; Gold, S. M.; Frew, A. J.; Aguilar, C.; Lin, D. R.; Umeda, E.; Voskuhl, R. R.; Alger, J. R. Cortical atrophy in experimental autoimmune encephalomyelitis: in vivo imaging. Neuroimage 60, 95-104, doi:http://dx.doi.org/10.1016/j.neuroimage.2011.11.099 (2012).

163 MacKenzie-Graham, A. T., M. R.; Shah, K. P.; Aguilar, C.; Strickland, L. V.; Boline, J.; Martin, M.; Morales, L.; Shattuck, D. W.; Jacobs, R. E.; Voskuhl, R. R.; Toga, A. W. Cerebellar cortical atrophy in experimental autoimmune encephalomyelitis. Neuroimage 32, 1016-1023 (2006).

164 MacKenzie-Graham, A. T.-W., S. K.; Sharma, G.; Aguilar, C.; Vo, K. T.; Strickland, L. V.; Morales, L.; Fubara, B.; Martin, M.; Jacobs, R. E.; Johnson, G. A.; Toga, A. W.; Voskuhl, R. R. Purkinje cell loss in experimental autoimmune encephalomyelitis. Neuroimage 48, 637-651, doi:http://dx.doi.org/10.1016/j.neuroimage.2009.06.073 (2009).

165 Magalon, K. Z., C.; Cayre, M.; Khaldi, J.; Bourbon, C.; Robles, I.; Tardif, G.; Viola, A.; Pruss, R. M.; Bordet, T.; Durbec, P. Olesoxime accelerates myelination and promotes repair in models of demyelination. Annals of Neurology 71, 213-226, doi:http://dx.doi.org/10.1002/ana.22593 (2012).

166 Maggi, P. M., S. M. C.; Gaitán, M. I.; Leibovitch, E.; Wholer, J. E.; Knight, H. L.; Ellis, M.; Wu, T.; Silva, A. C.; Massacesi, L.; Jacobson, S.; Westmoreland, S.; Reich, D. S. The formation of inflammatory demyelinated lesions in cerebral white matter. Annals of Neurology (2014).

167 Manogaran, P. et al. Exploring experimental autoimmune optic neuritis using multimodal imaging. Neuroimage 175, 327-339 (2018).

168 Mardiguian, S. S., S.; Ladds, E.; Campbell, S. J.; Wilainam, P.; McFadyen, C.; McAteer, M.; Choudhury, R. P.; Smith, P.; Saunders, F.; Watt, G.; Sibson, N. R.; Anthony, D. C. Anti-IL-17A treatment reduces clinical score and VCAM-1 expression detected by in vivo magnetic resonance imaging in chronic relapsing EAE ABH mice. American Journal of Pathology 182, 2071-2081, doi:http://dx.doi.org/10.1016/j.ajpath.2013.02.029 (2013).

169 Marriott, M. P. E., B.; Cate, H. S.; Binder, M. D.; Kemper, D.; Wu, Q.; Kolbe, S.; Gordon, I. R.; Wang, H.; Egan, G.; Murray, S.; Butzkueven, H.; Kilpatrick, T. J. Leukemia inhibitory factor signaling modulates both central nervous system demyelination and myelin repair. GLIA 56, 686-698, doi:http://dx.doi.org/10.1002/glia.20646 (2008).

170 Masthoff, M. et al. Temporal window for detection of inflammatory disease using dynamic cell tracking with time-lapse MRI. Sci Rep 8, 9563 (2018).

171 McAteer, M. A. S., N. R.; von Zur Muhlen, C.; Schneider, J. E.; Lowe, A. S.; Warrick, N.; Channon, K. M.; Anthony, D. C.; Choudhury, R. P. In vivo magnetic resonance imaging of acute brain inflammation using microparticles of iron oxide. Nature Medicine 13, 1253-1258 (2007).

172 McAteer, M. A. v. Z. M., C.; Anthony, D. C.; Sibson, N. R.; Choudhury, R. P. in Moleuclar Imaging: Methods and Protocols Vol. 680 Methods in Molecular Biology (ed K. Shah) 103-115 (Humana Press Inc, 2011).

173 McCreary, C. R. B., T. A.; Skihar, V.; Mitchell, J. R.; Yong, V. W.; Dunn, J. F. Multiexponential T2 and magnetization transfer MRI of demyelination and remyelination in murine spinal cord. Neuroimage 45, 1173-1182, doi:http://dx.doi.org/10.1016/j.neuroimage.2008.12.071 (2009).

174 Merkler, D. B., S.; Stadelmann, C.; Ernsting, T.; Michaelis, T.; Frahm, J.; Bruck, W. Multicontrast MRI of remyelination in the central nervous system. NMR in Biomedicine 18, 395-403 (2005).

175 Mi, S. H., B.; Hahm, K.; Luo, Y.; Kam Hui, E. S.; Yuan, Q.; Wong, W. M.; Wang, L.; Su, H.; Chu, T. H.; Guo, J.; Zhang, W.; So, K. F.; Pepinsky, B.; Shao, Z.; Graff, C.; Garber, E.; Jung, V.; Wu, E. X.; Wu, W. LINGO-1 antagonist promotes spinal cord remyelination and axonal integrity in MOG-induced experimental autoimmune encephalomyelitis. Nature Medicine 13, 1228-1233 (2007).

176 Miao, J. et al. Pleiotrophin regulates functional heterogeneity of microglia cells in EAE animal models of multiple sclerosis by activating CCr-7/CD206 molecules and functional cytokines. Am J Transl Res 11, 2013-2027 (2019).

177 Mikita, J. D.-C., N.; Deloire, M. S.; Vekris, A.; Biran, M.; Raffard, G.; Brochet, B.; Canron, M. H.; Franconi, J. M.; Boiziau, C.; Petry, K. G. Altered M1/M2 activation patterns of monocytes in severe relapsing experimental rat model of multiple sclerosis. Amelioration of clinical status by M2 activated monocyte administration. Multiple Sclerosis 17, 2-15, doi:http://dx.doi.org/10.1177/1352458510379243 (2011).

178 Millward, J. M. et al. Application of Europium-Doped Very Small Iron Oxide Nanoparticles to Visualize Neuroinflammation with MRI and Fluorescence Microscopy. Neuroscience 403, 136-144 (2019).

179 Millward, J. M. S., J.; Taupitz, M.; Wagner, S.; Wuerfel, J. T.; Infante-Duarte, C. Iron oxide magnetic nanoparticles highlight early involvement of the choroid plexus in central nervous system inflammation. ASN Neuro 5, e00110 (2013).

180 Modica, C. M. et al. Effect of teriflunomide on cortex-basal ganglia-thalamus (CxBGTh) circuit glutamatergic dysregulation in the Theiler's Murine Encephalomyelitis Virus mouse model of multiple sclerosis. PLoS One 12, e0182729 (2017).

181 Moon, J. H. J., H. W.; Lee, H. C.; Jeon, J. H.; Kim, N. H.; Sur, J. H.; Ha, J.; Jung, D. I. A study of experimental autoimmune encephalomyelitis in dogs as a disease model for canine necrotizing encephalitis. J vet sci 16, 203-211 (2015).

182 Morrissey, S. P. D., R.; Syha, J.; Simonis, C.; Zettl, U.; Archelos, J. J.; Jung, S.; Stodal, H.; Lassmann, H.; Toyka, K. V.; Haase, A.; Hartung, H. P. Partial inhibition of AT-EAE by an antibody to ICAM-1: clinico-histological and MRI studies. Journal of Neuroimmunology 69, 85-93 (1996).

183 Morrissey, S. P. S., H.; Zettl, U.; Simonis, C.; Jung, S.; Kiefer, R.; Lassmann, H.; Hartung, H. P.; Haase, A.; Toyka, K. V. In vivo MRI and its histological correlates in acute adoptive transfer experimental allergic encephalomyelitis. Quantification of inflammation and oedema. Brain 119, 239-248 (1996).

184 Muja, N. C., M. E.; Zhang, J.; Kim, H.; Gilad, A. A.; Walczak, P.; Ben-Hur, T.; Bulte, J. W. Neural precursors exhibit distinctly different patterns of cell migration upon transplantation during either the acute or chronic phase of EAE: a serial MR imaging study. Magnetic Resonance in Medicine 65, 1738-1749, doi:http://dx.doi.org/10.1002/mrm.22757 (2011).

185 Namaer, I. J. S., J.; Poulet, P.; Armspach, J. P.; Mauss, Y.; Chambron, J. In vivo dynamic MR imaging of MBP‐induced acute experimental allergic encephalomyelitis in Lewis rat. Magnetic Resonance in Medicine 24, 325-334, doi:10.1002/mrm.1910240213 (1992).

186 Namer, I. J. S., J.; Poulet, P.; Armspach, J. P.; Mohr, M.; Mauss, Y.; Chambron, J. Blood-brain barrier breakdown in MBP-specific T cell induced experimental allergic encephalomyelitis. A quantitative in vivo MRI study. Brain 116, 147-159 (1993).

187 Namer, I. J. S., J.; Piddlesden, S. J.; Mohr, M.; Poulet, P.; Chambron, J. Magnetic resonance imaging of antibody-mediated demyelinating experimental allergic encephalomyelitis. Journal of Neuroimmunology 54, 41-50 (1994).

188 Namer, I. J. S., J.; Poulet, P.; Mauss, Y.; Armspach, J. P.; Eclancher, B.; Chambron, J. Hyperbaric oxygen treatment in acute experimental allergic encephalomyelitis. Contribution of magnetic resonance imaging study. Neuroimage 1, 308-312 (1994).

189 Namer, I. J. S., J.; Klinguer, C.; Trifilieff, E.; Mohr, M.; Poulet, P. Magnetic resonance imaging of PLP-induced experimental allergic encephalomyelitis in Lewis rats. Journal of Neuroimmunology 92, 22-28 (1998).

190 Nathoo, N. A., S.; Wu, Y.; Haylock-Jacobs, S.; Yong, V. W.; Foniok, T.; Barnes, S.; Obenaus, A.; Dunn, J. F. Susceptibility-weighted imaging in the experimental autoimmune encephalomyelitis model of multiple sclerosis indicates elevated deoxyhemoglobin, iron deposition and demyelination. Multiple Sclerosis 19, 721-731, doi:http://dx.doi.org/10.1177/1352458512460602 (2013).

191 Nathoo, N. R., J. A.; Yong, V. W.; Dunn, J. F. Detecting deoxyhemoglobin in spinal cord vasculature of the experimental autoimmune encephalomyelitis mouse model of multiple sclerosis using susceptibility MRI and hyperoxygenation. PLoS ONE 10, e0127033, doi:http://dx.doi.org/10.1371/journal.pone.0127033 (2015).

192 Nessler, S. B., S.; Stadelmann, C.; Bittner, A.; Merkler, D.; Hartung, H. P.; Michaelis, T.; Bruck, W.; Frahm, J.; Sommer, N.; Hemmer, B. Early MRI changes in a mouse model of multiple sclerosis are predictive of severe inflammatory tissue damage. Brain 130, 2186-2198 (2007).

193 Nie, T. T. Y., G.; Jia, Y. L.; Zhang, T.; Shen, Z. W.; Zhang, H. D.; Xu, H. Y.; Wu, R. H. Region-Specific Susceptibilities to Cuprizone-Induced Demyelination of C57BL/6 Mouse: In vivo T2WI and DTI Studies at 7.0T. Appl. Magn. Reson. 45, 759-769, doi:10.1007/s00723-014-0553-3 (2014).

194 Nishioka, C., Liang, H. F., Barsamian, B. & Sun, S. W. Sequential phases of RGC axonal and somatic injury in EAE mice examined using DTI and OCT. Mult Scler Relat Disord 27, 315-323 (2019).

195 Nishioka, C., Liang, H. F., Chung, C. F. & Sun, S. W. Disease stage-dependent relationship between diffusion tensor imaging and electrophysiology of the visual system in a murine model of multiple sclerosis. Neuroradiology 59, 1241-1250 (2017).

196 Noseworthy, J. H. G., J. J.; Vandervoort, M. K.; Karlik, S. J. Postnatal NMR changes in guinea pig central nervous system: potential relevance to experimental allergic encephalomyelitis. Magnetic Resonance in Medicine 6, 199-211 (1988).

197 Noth, U. M., S. P.; Deichmann, R.; Jung, S.; Adolf, H.; Haase, A.; Lutz, J. Perfluoro-15-crown-5-ether labelled macrophages in adoptive transfer experimental allergic encephalomyelitis. Artif Cells Blood Substit Immobil Biotechnol 25, 243-254 (1997).

198 Oakden, W., Bock, N. A., Al-Ebraheem, A., Farquharson, M. J. & Stanisz, G. J. Early regional cuprizone-induced demyelination in a rat model revealed with MRI. NMR in biomedicine 30 (2017).

199 Orije, J. K., F.; Guglielmetti, C.; Praet, J.; Van der Linden, A.; Ponsaerts, P.; Verhoye, M. Longitudinal monitoring of metabolic alterations in cuprizone mouse model of multiple sclerosis using 1H-magnetic resonance spectroscopy. Neuroimage 114, 128-135, doi:http://dx.doi.org/10.1016/j.neuroimage.2015.04.012 (2015).

200 Oude Engberink, R. D. B., E. L.; Dijkstra, C. D.; van der Pol, S. M.; van der Toorn, A.; de Vries, H. E. Dynamics and fate of USPIO in the central nervous system in experimental autoimmune encephalomyelitis. NMR in Biomedicine 23, 1087-1096, doi:http://dx.doi.org/10.1002/nbm.1536 (2010).

201 Oweida, A. J. D., E. A.; Foster, P. J. Cellular imaging at 1.5 T: detecting cells in neuroinflammation using active labeling with superparamagnetic iron oxide. Mol Imaging 3, 85-95 (2004).

202 Oweida, A. J. D., E. A.; Karlik, S. J.; Dekaban, G. A.; Foster, P. J. Iron-oxide labeling of hematogenous macrophages in a model of experimental autoimmune encephalomyelitis and the contribution to signal loss in fast imaging employing steady state acquisition (FIESTA) images. Journal of Magnetic Resonance Imaging 26, 144-151 (2007).

203 Palazuelos, J. D., N.; Julien, B.; Hatterer, E.; Aguado, T.; Mechoulam, R.; Benito, C.; Romero, J.; Silva, A.; Guzmán, M.; Nataf, S.; Galve-Roperh, I. The CB2 cannabinoid receptor controls myeloid progenitor trafficking: Involvement in the pathogenesis of an animal model of multiple sclerosis. Journal of Biological Chemistry 283, 13320-13324 (2008).

204 Paz Soldan, M. M. R., M. R.; Gamez, J. D.; Lohrey, A. K.; Chen, Y.; Pirko, I.; Johnson, A. J. Correlation of Brain Atrophy, Disability, and Spinal Cord Atrophy in a Murine Model of Multiple Sclerosis. Journal of Neuroimaging 25, 595-599, doi:http://dx.doi.org/10.1111/jon.12250 (2015).

205 Peersman, G. V. V. d. V., F. L.; Lohman, J. E.; Lubke, U.; Gheuens, J.; Bellon, E.; Connelly, A.; Martin, J. J. High resolution nuclear magnetic resonance imaging of the spinal cord in experimental demyelinating disease. Acta Neuropathol (Berl) 76, 628-632 (1988).

206 Petiet, A. A., M. S.; Stankoff, B. Gray and White Matter Demyelination and Remyelination Detected with Multimodal Quantitative MRI Analysis at 11.7T in a Chronic Mouse Model of Multiple Sclerosis. Front 10, 491 (2016).

207 Pettersson, Å. W., X. C.; Ciumas, C.; Lian, H.; Chirsky, V.; Huang, Y. M.; Bjelke, B.; Link, H.; Xiao, B. G. CD8α+ dendritic cells and immune protection from experimental allergic encephalomyelitis. Clinical and Experimental Immunology 137, 486-495 (2004).

208 Piraino, P. S. Y., T. A.; Freedman, S. B.; Messersmith, E. K.; Pleiss, M. A.; Karlik, S. J. Suppression of acute experimental allergic encephalomyelitis with a small molecule inhibitor of alpha4 integrin. Multiple Sclerosis 11, 683-690 (2005).

209 Pirko, I. C., B.; Johnson, A. J.; Gamez, J.; Rodriguez, M.; Macura, S. Magnetic resonance imaging of immune cells in inflammation of central nervous system. Croat Med J 44, 463-468 (2003).

210 Pirko, I. C., B.; Gamez, J.; Bieber, A. J.; Warrington, A. E.; Johnson, A. J.; Hanson, D. P.; Pease, L. R.; Macura, S. I.; Rodriguez, M. A human antibody that promotes remyelination enters the CNS and decreases lesion load as detected by T2-weighted spinal cord MRI in a virus-induced murine model of MS. FASEB J. 18, 1577-1579 (2004).

211 Pirko, I. C., R.; Chen, Y.; Lohrey, A. K.; Lindquist, D. M.; Dunn, R. S.; Zivadinov, R.; Johnson, A. J. CMV infection attenuates the disease course in a murine model of multiple sclerosis. PLoS ONE 7, e32767, doi:http://dx.doi.org/10.1371/journal.pone.0032767 (2012).

212 Pirko, I. C., Y.; Lohrey, A. K.; McDole, J.; Gamez, J. D.; Allen, K. S.; Pavelko, K. D.; Lindquist, D. M.; Dunn, R. S.; Macura, S. I.; Johnson, A. J. Contrasting roles for CD4 vs. CD8 T-cells in a murine model of virally induced "T1 black hole" formation. PLoS ONE 7, e31459, doi:http://dx.doi.org/10.1371/journal.pone.0031459 (2012).

213 Pirko, I. G., J.; Johnson, A. J.; Macura, S. I.; Rodriguez, M. Dynamics of MRI lesion development in an animal model of viral-induced acute progressive CNS demyelination. Neuroimage 21, 576-582 (2004).

214 Pirko, I. J., A.; Ciric, B.; Gamez, J.; Macura, S. I.; Pease, L. R.; Rodriguez, M. In vivo magnetic resonance imaging of immune cells in the central nervous system with superparamagnetic antibodies. FASEB J. 18, 179-182 (2004).

215 Pirko, I. J., A.; Gamez, J.; Macura, S. I.; Rodriguez, M. Disappearing "T1 black holes" in an animal model of multiple sclerosis. Front Biosci 9, 1222-1227 (2004).

216 Pirko, I. J., A. J.; Lohrey, A. K.; Chen, Y.; Ying, J. Deep gray matter T2 hypointensity correlates with disability in a murine model of MS. Journal of the Neurological Sciences 282, 34-38, doi:http://dx.doi.org/10.1016/j.jns.2008.12.013 (2009).

217 Pirko, I. J., A. J.; Chen, Y.; Lindquist, D. M.; Lohrey, A. K.; Ying, J.; Dunn, R. S. Brain atrophy correlates with functional outcome in a murine model of multiple sclerosis. Neuroimage 54, 802-806, doi:http://dx.doi.org/10.1016/j.neuroimage.2010.08.055 (2011).

218 Pol, S. et al. Characterization of leptomeningeal inflammation in rodent experimental autoimmune encephalomyelitis (EAE) model of multiple sclerosis. Exp Neurol 314, 82-90 (2019).

219 Pol, S. et al. Teriflunomide's Effect on Glia in Experimental Demyelinating Disease: A Neuroimaging and Histologic Study. J Neuroimaging 29, 52-61 (2019).

220 Politi, L. S. B., M.; Brambilla, E.; Cadioli, M.; Falini, A.; Comi, G.; Scotti, G.; Martino, G.; Pluchino, S. Magnetic-resonance-based tracking and quantification of intravenously injected neural stem cell accumulation in the brains of mice with experimental multiple sclerosis. Stem Cells 25, 2583-2592 (2007).

221 Praet, J. O., J.; Kara, F.; Guglielmetti, C.; Santermans, E.; Daans, J.; Hens, N.; Verhoye, M.; Berneman, Z.; Ponsaerts, P.; Van der Linden, A. Cuprizone-induced demyelination and demyelination-associated inflammation result in different proton magnetic resonance metabolite spectra. NMR in Biomedicine 28, 505-513, doi:http://dx.doi.org/10.1002/nbm.3277 (2015).

222 Qi, X. L., A. S.; Sun, L.; Hauswirth, W. W.; Guy, J. Suppression of mitochondrial oxidative stress provides long-term neuroprotection in experimental optic neuritis. Invest Ophthalmol Vis Sci 48, 681-691 (2007).

223 Qi, X. S., L.; Lewin, A. S.; Hauswirth, W. W.; Guy, J. Long-term suppression of neurodegeneration in chronic experimental optic neuritis: antioxidant gene therapy. Invest Ophthalmol Vis Sci 48, 5360-5370 (2007).

224 Rausch, M. H., P.; Baumann, D.; Cannet, C.; Rudin, M. MRI-based monitoring of inflammation and tissue damage in acute and chronic relapsing EAE. Magnetic Resonance in Medicine 50, 309-314 (2003).

225 Rausch, M. H., P.; Foster, C. A.; Baumann, D. R.; Cannet, C.; Rudin, M. Predictability of FTY720 efficacy in experimental autoimmune encephalomyelitis by in vivo macrophage tracking: clinical implications for ultrasmall superparamagnetic iron oxide-enhanced magnetic resonance imaging. Journal of Magnetic Resonance Imaging 20, 16-24 (2004).

226 Rausch, M. T., P.; Lervik, P.; Walmsley, A.; Mir, A.; Schubart, A.; Seabrook, T. Characterization of white matter damage in animal models of multiple sclerosis by magnetization transfer ratio and quantitative mapping of the apparent bound proton fraction f. Multiple Sclerosis 15, 16-27, doi:http://dx.doi.org/10.1177/1352458508096006 (2009).

227 Richards, T. L. A., E. C., Jr.; Peterson, J.; Cosgrove, S.; Petersen, R.; Petersen, K.; Heide, A. C.; Cluff, J.; Rose, L. M. Experimental allergic encephalomyelitis in non-human primates: MRI and MRS may predict the type of brain damage. NMR in Biomedicine 8, 49-58 (1995).

228 Robinson, K. M. N., J. M.; Phillips, D. A.; Proctor, T. M.; Rooney, W. D.; Jones, R. E. MR imaging of inflammation during myelin-specific T cell-mediated autoimmune attack in the EAE mouse spinal cord. Mol Imaging Biol 12, 240-249, doi:http://dx.doi.org/10.1007/s11307-009-0272-6 (2010).

229 Rose, L. M. R., T. L.; Alvord, E. C., Jr. Magnetic resonance imaging and peripheral blood abnormalities in experimental allergic encephalomyelitis. Biomed Pharmacother 43, 347-353 (1989).

230 Rose, L. M. R., T. L.; Peterson, J.; Petersen, R.; Alvord, E. C., Jr. Resolution of CNS lesions following treatment of experimental allergic encephalomyelitis in macaques with monoclonal antibody to the CD18 leukocyte integrin. Multiple Sclerosis 2, 259-266 (1997).

231 Saskia Hübner, N. M., A. E.; Lee, H. L.; Reisert, M.; Bienert, T.; Hennig, J.; von Elverfeldt, D.; Harsan, L. A. The connectomics of brain demyelination: Functional and structural patterns in the cuprizone mouse model. NeuroImage 146, 1-18 (2017).

232 Schellenberg, A. E. B., R.; Yong, V. W.; Del Bigio, M. R.; Peeling, J. Magnetic resonance imaging of blood-spinal cord barrier disruption in mice with experimental autoimmune encephalomyelitis. Magnetic Resonance in Medicine 58, 298-305 (2007).

233 Schneider, C. S., G.; Zollner, T. M. Acute neuroinflammation in Lewis rats - a model for acute multiple sclerosis relapses. Journal of Neuroimmunology 213, 84-90, doi:http://dx.doi.org/10.1016/j.jneuroim.2009.05.015 (2009).

234 Seeldrayers, P. A. S., J.; Morrissey, S. P.; Stodal, H.; Vass, K.; Jung, S.; Gneiting, T.; Lassmann, H.; Haase, A.; Hartung, H. P.; et al.,. Magnetic resonance imaging investigation of blood-brain barrier damage in adoptive transfer experimental autoimmune encephalomyelitis. Journal of Neuroimmunology 46, 199-206 (1993).

235 Serguera, C. et al. Anti-MOG autoantibodies pathogenicity in children and macaques demyelinating diseases. J Neuroinflammation 16, 244 (2019).

236 Serres, S. A., D. C.; Jiang, Y.; Broom, K. A.; Campbell, S. J.; Tyler, D. J.; van Kasteren, S. I.; Davis, B. G.; Sibson, N. R. Systemic inflammatory response reactivates immune-mediated lesions in rat brain. Journal of Neuroscience 29, 4820-4828, doi:http://dx.doi.org/10.1523/JNEUROSCI.0406-09.2009 (2009).

237 Serres, S. B., C.; de Pablos, R. M.; Merkler, D.; Soto, M. S.; Sibson, N. R.; Anthony, D. C. Magnetic resonance imaging reveals therapeutic effects of interferon-beta on cytokine-induced reactivation of rat model of multiple sclerosis. J Cereb Blood Flow Metab 33, 744-753, doi:http://dx.doi.org/10.1038/jcbfm.2013.12 (2013).

238 Serres, S. M., S.; Campbell, S. J.; McAteer, M. A.; Akhtar, A.; Krapitchev, A.; Choudhury, R. P.; Anthony, D. C.; Sibson, N. R. VCAM-1-targeted magnetic resonance imaging reveals subclinical disease in a mouse model of multiple sclerosis. FASEB J. 25, 4415-4422, doi:http://dx.doi.org/10.1096/fj.11-183772 (2011).

239 Sibson, N. R. B., A. M.; Bernades-Silva, M.; Laurent, S.; Boutry, S.; Muller, R. N.; Styles, P.; Anthony, D. C. MRI detection of early endothelial activation in brain inflammation. Magnetic Resonance in Medicine 51, 248-252 (2004).

240 Silva, B. A. et al. A new focal model resembling features of cortical pathology of the progressive forms of multiple sclerosis: Influence of innate immunity. Brain Behav Immun 69, 515-531 (2018).

241 Singer, B. A. T., N. J.; Frank, J. A.; McFarland, H. F.; Biddison, W. E. Induction of experimental allergic encephalomyelitis in the NIH minipig. Journal of Neuroimmunology 105, 7-19 (2000).

242 Sipkins, D. A. G., K.; Tropper, F. D.; Bednarski, M.; Li, K. C. P.; Steinman, L. ICAM-1 expression in autoimmune encephalitis visualized using magnetic resonance imaging. Journal of Neuroimmunology 104, 1-9 (2000).

243 Smith, P. A. et al. Fingolimod inhibits brain atrophy and promotes brain-derived neurotrophic factor in an animal model of multiple sclerosis. Journal of neuroimmunology 318, 103-113 (2018).

244 Song, S. K. Y., J.; Le, T. Q.; Lin, S. J.; Sun, S. W.; Cross, A. H.; Armstrong, R. C. Demyelination increases radial diffusivity in corpus callosum of mouse brain. Neuroimage 26, 132-140 (2005).

245 Soustelle, L. et al. Correlations of quantitative MRI metrics with myelin basic protein (MBP) staining in a murine model of demyelination. NMR in biomedicine 32, e4116 (2019).

246 Stassart, R. M. H., G.; Garea-Rodríguez, E.; Nessler, S.; Hayardeny, L.; Wegner, C.; Schlumbohm, C.; Fuchs, E.; Brück, W. A New Targeted Model of Experimental Autoimmune Encephalomyelitis in the Common Marmoset. Brain Pathology 26, 452-464 (2016).

247 Steinbrecher, A. W., T.; Neuberger, T.; Mueller, A. M.; Pedre, X.; Giegerich, G.; Bogdahn, U.; Jakob, P.; Haase, A.; Faber, C. Experimental autoimmune encephalomyelitis in the rat spinal cord: lesion detection with high-resolution MR microscopy at 17.6 T. AJNR Am J Neuroradiol 26, 19-25 (2005).

248 Stewart, W. A. A., E. C.; Hruby, S.; Hall, L. D.; Paty, D. W. Early detection of experimental allergic encephalomyelitis by magnetic resonance imaging. Lancet 2, 898 (1985).

249 Stewart, W. A. A., E. C., Jr.; Hruby, S.; Hall, L. D.; Paty, D. W. Magnetic resonance imaging of experimental allergic encephalomyelitis in primates. Brain 114, 1069-1096 (1991).

250 Stoll, G. W., C.; Gold, R.; Solymosi, L.; Toyka, K. V.; Bendszus, M. In vivo monitoring of macrophage infiltration in experimental autoimmune neuritis by magnetic resonance imaging. Journal of Neuroimmunology 149, 142-146 (2004).

251 Sun, S. W. L., H. F.; Trinkaus, K.; Cross, A. H.; Armstrong, R. C.; Song, S. K. Noninvasive detection of cuprizone induced axonal damage and demyelination in the mouse corpus callosum. Magnetic Resonance in Medicine 55, 302-308 (2006).

252 Sun, S. W. L., H. F.; Schmidt, R. E.; Cross, A. H.; Song, S. K. Selective vulnerability of cerebral white matter in a murine model of multiple sclerosis detected using diffusion tensor imaging. Neurobiology of Disease 28, 30-38 (2007).

253 T Hart, B. A. B., J.; Muller, H. J.; Melchers, B.; Nicolay, K.; Brok, H.; Bontrop, R. E.; Lassmann, H.; Massacesi, L. Histopathological characterization of magnetic resonance imaging- detectable brain white matter lesions in a primate model of multiple sclerosis: A correlative study in the experimental autoimmune encephalomyelitis model in common marmosets (Callithrix jacchus). American Journal of Pathology 153, 649-663 (1998).

254 t Hart, B. A. B., E. L.; Brok, H. P.; Boon, L.; de Boer, M.; Bauer, J.; Laman, J. D. Treatment with chimeric anti-human CD40 antibody suppresses MRI-detectable inflammation and enlargement of pre-existing brain lesions in common marmosets affected by MOG-induced EAE. Journal of Neuroimmunology 163, 31-39 (2005).

255 t Hart, B. A. B., H. P.; Remarque, E.; Benson, J.; Treacy, G.; Amor, S.; Hintzen, R. Q.; Laman, J. D.; Bauer, J.; Blezer, E. L. Suppression of ongoing disease in a nonhuman primate model of multiple sclerosis by a human-anti-human IL-12p40 antibody. Journal of Immunology 175, 4761-4768 (2005).

256 Tagge, I. O. C., A.; Chaudhary, P.; Pollaro, J.; Berlow, Y.; Chalupsky, M.; Bourdette, D.; Woltjer, R.; Johnson, M.; Rooney, W. Spatio-Temporal Patterns of Demyelination and Remyelination in the Cuprizone Mouse Model. PLoS ONE 11, e0152480, doi:http://dx.doi.org/10.1371/journal.pone.0152480 (2016).

257 Talbott, J. F. N.-L., Y. S.; Wendland, M. F.; Mukherjee, P.; Huie, J. R.; Hess, C. P.; Mabray, M. C.; Bresnahan, J. C.; Beattie, M. S. Diffusion-Weighted Magnetic Resonance Imaging Characterization of White Matter Injury Produced by Axon-Sparing Demyelination and Severe Contusion Spinal Cord Injury in Rats. Journal of Neurotrauma 33, 929-942, doi:http://dx.doi.org/10.1089/neu.2015.4102 (2016).

258 Talla, V. Y., C.; Shaw, G.; Porciatti, V.; Koilkonda, R. D.; Guy, J. Noninvasive assessments of optic nerve neurodegeneration in transgenic mice with isolated optic neuritis. Invest Ophthalmol Vis Sci 54, 4440-4450, doi:http://dx.doi.org/10.1167/iovs.13-11899 (2013).

259 Tambalo, S. P.-J., L.; Rigolio, R.; Fiorini, S.; Bontempi, P.; Mallucci, G.; Balzarotti, B.; Marmiroli, P.; Sbarbati, A.; Cavaletti, G.; Pluchino, S.; Marzola, P. Functional Magnetic Resonance Imaging of Rats with Experimental Autoimmune Encephalomyelitis Reveals Brain Cortex Remodeling. Journal of Neuroscience 35, 10088-10100, doi:http://dx.doi.org/10.1523/JNEUROSCI.0540-15.2015 (2015).

260 Thiessen, J. D. Z., Y.; Zhang, H.; Wang, L.; Buist, R.; Del Bigio, M. R.; Kong, J.; Li, X. M.; Martin, M. Quantitative MRI and ultrastructural examination of the cuprizone mouse model of demyelination. NMR in Biomedicine 26, 1562-1581, doi:http://dx.doi.org/10.1002/nbm.2992 (2013).

261 Thomas, A. M. et al. Evaluation of cell transplant-mediated attenuation of diffuse injury in experimental autoimmune encephalomyelitis using onVDMP CEST MRI. Exp Neurol 329, 113316 (2020).

262 Tobin, J. E. X., M.; Le, T. Q.; Song, S. K.; Armstrong, R. C. Reduced axonopathy and enhanced remyelination after chronic demyelination in fibroblast growth factor 2 (Fgf2)-null mice: differential detection with diffusion tensor imaging. J Neuropathol Exp Neurol 70, 157-165, doi:http://dx.doi.org/10.1097/NEN.0b013e31820937e4 (2011).

263 Torkildsen, O. B., L. A.; Thorsen, F.; Mork, S. J.; Stangel, M.; Myhr, K. M.; Bo, L. Effects of dietary intervention on MRI activity, de- and remyelination in the cuprizone model for demyelination. Experimental Neurology 215, 160-166, doi:http://dx.doi.org/10.1016/j.expneurol.2008.09.026 (2009).

264 Tourdias, T. H., B.; Raffard, G.; Biran, M.; Nishiguchi, T.; Aussudre, J.; Franconi, J. M.; Brochet, B.; Petry, K. G.; Dousset, V. Adapted focal experimental autoimmune encephalomyelitis to allow MRI exploration of multiple sclerosis features. Experimental Neurology 230, 248-257, doi:http://dx.doi.org/10.1016/j.expneurol.2011.04.023 (2011).

265 Tourdias, T. M., N.; Dragonu, I.; Cassagno, N.; Boiziau, C.; Aussudre, J.; Brochet, B.; Moonen, C.; Petry, K. G.; Dousset, V. Differential aquaporin 4 expression during edema build-up and resolution phases of brain inflammation. Journal of Neuroinflammation 8, 143, doi:http://dx.doi.org/10.1186/1742-2094-8-143 (2011).

266 Turati, L. M., M.; Mastropietro, A.; Dowell, N. G.; Zucca, I.; Erbetta, A.; Cordiglieri, C.; Brenna, G.; Bianchi, B.; Mantegazza, R.; Cercignani, M.; Baggi, F.; Minati, L. In vivo quantitative magnetization transfer imaging correlates with histology during de- and remyelination in cuprizone-treated mice. NMR in Biomedicine 28, 327-337, doi:http://dx.doi.org/10.1002/nbm.3253 (2015).

267 Tysiak, E. A., P.; Aktas, O.; Waiczies, H.; Smyth, M.; Schnorr, J.; Taupitz, M.; Wuerfel, J. Beyond blood brain barrier breakdown - in vivo detection of occult neuroinflammatory foci by magnetic nanoparticles in high field MRI. Journal of Neuroinflammation 6, 20, doi:http://dx.doi.org/10.1186/1742-2094-6-20 (2009).

268 Van Lambalgen, R. J., M. Experimental allergic encephalomyelitis in rhesus monkeys: II. Treatment of EAE with anti-T lymphocyte subset monoclonal antibodies. Clin Exp Immunol 68, 305-312 (1987).

269 Varga, E. et al. Cuprizone Administration Alters the Iron Metabolism in the Mouse Model of Multiple Sclerosis. Cell Mol Neurobiol 38, 1081-1097 (2018).

270 Verhoye, M. R. G., E. J.; Raman, E. R.; Van Reempts, J.; Van der Linden, A. In vivo noninvasive determination of abnormal water diffusion in the rat brain studied in an animal model for multiple sclerosis by diffusion-weighted NMR imaging. Magn Reson Imaging 14, 521-532 (1996).

271 Vowinckel, E. R., D.; Becher, B.; Verge, G.; Evans, A.; Owens, T.; Antel, J. P. PK11195 binding to the peripheral benzodiazepine receptor as a marker of microglia activation in multiple sclerosis and experimental autoimmune encephalomyelitis. Journal of Neuroscience Research 50, 345-353 (1997).

272 Waiczies, H. L., S.; Drechsler, S.; Qadri, F.; Purfurst, B.; Sydow, K.; Dathe, M.; Kuhne, A.; Lindel, T.; Hoffmann, W.; Pohlmann, A.; Niendorf, T.; Waiczies, S. Visualizing brain inflammation with a shingled-leg radio-frequency head probe for 19F/1H MRI. Sci 3, 1280, doi:http://dx.doi.org/10.1038/srep01280 (2013).

273 Waiczies, H. M., J. M.; Lepore, S.; Infante-Duarte, C.; Pohlmann, A.; Niendorf, T.; Waiczies, S. Identification of cellular infiltrates during early stages of brain inflammation with magnetic resonance microscopy. PLoS ONE 7, e32796, doi:http://dx.doi.org/10.1371/journal.pone.0032796 (2012).

274 Waiczies, S. et al. Enhanced Fluorine-19 MRI Sensitivity using a Cryogenic Radiofrequency Probe: Technical Developments and Ex Vivo Demonstration in a Mouse Model of Neuroinflammation. Sci Rep 7, 9808 (2017).

275 Waiczies, S. et al. Fluorine-19 MRI at 21.1 T: enhanced spin-lattice relaxation of perfluoro-15-crown-5-ether and sensitivity as demonstrated in ex vivo murine neuroinflammation. Magma 32, 37-49 (2019).

276 Wang, N. et al. Neurite orientation dispersion and density imaging of mouse brain microstructure. Brain Struct Funct 224, 1797-1813 (2019).

277 Wang, N. et al. Probing demyelination and remyelination of the cuprizone mouse model using multimodality MRI. J Magn Reson Imaging 50, 1852-1865 (2019).

278 Wang, X. B., J. K.; Kim, J. H.; Chen, Y. J.; O'Neal, J.; O'Neil, S. P.; Tu, T. W.; Trinkaus, K.; Song, S. K. Diffusion tensor imaging detects treatment effects of FTY720 in experimental autoimmune encephalomyelitis mice. NMR in Biomedicine 26, 1742-1750 (2013).

279 Wang, X. C., M. F.; Wang, Y.; Sun, P.; Libbey, J. E.; Trinkaus, K.; Fujinami, R. S.; Song, S. K. Diffusion basis spectrum imaging detects and distinguishes coexisting subclinical inflammation, demyelination and axonal injury in experimental autoimmune encephalomyelitis mice. NMR in Biomedicine 27, 843-852 (2014).

280 Wang, Y. W., Q.; Haldar, J. P.; Yeh, F. C.; Xie, M.; Sun, P.; Tu, T. W.; Trinkaus, K.; Klein, R. S.; Cross, A. H.; Song, S. K. Quantification of increased cellularity during inflammatory demyelination. Brain 134, 3590-3601, doi:http://dx.doi.org/10.1093/brain/awr307 (2011).

281 Williams, R. R., A. M.; Wang, W. T.; Choi, I. Y.; Lee, P.; Berman, N. E.; Lynch, S. G.; LeVine, S. M. Iron deposition is independent of cellular inflammation in a cerebral model of multiple sclerosis. BMC Neurosci 12, 59, doi:http://dx.doi.org/10.1186/1471-2202-12-59 (2011).

282 Wood, T. C. S., C.; Hurley, S. A.; Vernon, A. C.; Torres, J.; Dell'Acqua, F.; Williams, S. C. R.; Cash, D. Whole-brain ex-vivo quantitative MRI of the cuprizone mouse model. PeerJ 2016 (2016).

283 Wu, Q. Z. Y., Q.; Cate, H. S.; Kemper, D.; Binder, M.; Wang, H. X.; Fang, K.; Quick, M. J.; Marriott, M.; Kilpatrick, T. J.; Egan, G. F. MRI identification of the rostral-caudal pattern of pathology within the corpus callosum in the cuprizone mouse model. Journal of Magnetic Resonance Imaging 27, 446-453 (2008).

284 Wuerfel, E. I.-D., C.; Glumm, R.; Wuerfel, J. T. Gadofluorine M-enhanced MRI shows involvement of circumventricular organs in neuroinflammation. Journal of Neuroinflammation 7, 70, doi:http://dx.doi.org/10.1186/1742-2094-7-70 (2010).

285 Wuerfel, J. T., E.; Prozorovski, T.; Smyth, M.; Mueller, S.; Schnorr, J.; Taupitz, M.; Zipp, F. Mouse model mimics multiple sclerosis in the clinico-radiological paradox. Eur J Neurosci 26, 190-198 (2007).

286 Xiao, B. G. W., X. C.; Yang, J. S.; Xu, L. Y.; Liu, X.; Huang, Y. M.; Bjelke, B.; Link, H. Therapeutic potential of IFN-gamma-modified dendritic cells in acute and chronic experimental allergic encephalomyelitis. Int Immunol 16, 13-22 (2004).

287 Xu, S. J., E. K.; Brocke, S.; Bulte, J. W.; Quigley, L.; Tresser, N.; Ostuni, J. L.; Yang, Y.; McFarland, H. F.; Frank, J. A. Study of relapsing remitting experimental allergic encephalomyelitis SJL mouse model using MION-46L enhanced in vivo MRI: early histopathological correlation. Journal of Neuroscience Research 52, 549-558 (1998).

288 Xu, S. J., E. K.; Li, W.; Yang, Y.; Chesnick, S. A.; Webster, H. D.; Brocke, S.; Quigley, L.; McFarland, H. F.; Frank, J. A. In vivo three-dimensional MR microscopy of mice with chronic relapsing experimental autoimmune encephalomyelitis after treatment with insulin-like growth factor-I. AJNR Am J Neuroradiol 19, 653-658 (1998).

289 Yano, R. et al. Quantitative temporal changes in DTI values coupled with histological properties in cuprizone-induced demyelination and remyelination. Neurochem Int 119, 151-158 (2018).

290 Yu, O. S., J.; Mauss, Y.; Guignard, B.; Eclancher, B.; Chambron, J.; Grucker, D. Remyelination assessment by MRI texture analysis in a cuprizone mouse model. Magn Reson Imaging 22, 1139-1144 (2004).

291 Zaaraoui, W. D., M.; Merle, M.; Girard, C.; Raffard, G.; Biran, M.; Inglese, M.; Petry, K. G.; Gonen, O.; Brochet, B.; Franconi, J. M.; Dousset, V. Monitoring demyelination and remyelination by magnetization transfer imaging in the mouse brain at 9.4 T. Magma 21, 357-362, doi:http://dx.doi.org/10.1007/s10334-008-0141-3 (2008).

292 Zhang, J. J., M. V.; McMahon, M. T.; Mori, S.; Calabresi, P. A. In vivo and ex vivo diffusion tensor imaging of cuprizone-induced demyelination in the mouse corpus callosum. Magnetic Resonance in Medicine 67, 750-759 (2012).

293 Zhang, X. et al. IL-11 antagonist suppresses Th17 cell-mediated neuroinflammation and demyelination in a mouse model of relapsing-remitting multiple sclerosis. Clin Immunol 197, 45-53 (2018).

294 Zhang, Y. et al. Multimodal Molecular Imaging Demonstrates Myeloperoxidase Regulation of Matrix Metalloproteinase Activity in Neuroinflammation. Mol Neurobiol 56, 954-962 (2019).

295 Zhang, Y. W., J.; Buist, R.; Peeling, J.; Yong, V. W.; Mitchell, J. R. A novel MRI texture analysis of demyelination and inflammation in relapsing-remitting experimental allergic encephalomyelitis. Med Image Comput Comput Assist Interv Int Conf Med Image Comput Comput Assist Interv 9, 760-767 (2006).

296 Zhang, Y. W., J.; Buist, R.; Peeling, J.; Yong, V. W.; Mitchell, J. R. Active inflammation increases the heterogeneity of MRI texture in mice with relapsing experimental allergic encephalomyelitis. Magn Reson Imaging 32, 168-174, doi:http://dx.doi.org/10.1016/j.mri.2013.10.006 (2014).

297 Zhao, P. Y. et al. Bu Shen Yi Sui capsule promotes remyelination correlating with Sema3A/NRP-1, LIF/LIFR and Nkx6.2 in mice with experimental autoimmune encephalomyelitis. J Ethnopharmacol 217, 36-48 (2018).

298 Zhong, J. N., K.; Morel, P. A.; Xu, H.; Ahrens, E. T. In Vivo Quantification of Inflammation in Experimental Autoimmune Encephalomyelitis Rats Using Fluorine-19 Magnetic Resonance Imaging Reveals Immune Cell Recruitment outside the Nervous System. PLoS ONE 10, e0140238, doi:http://dx.doi.org/10.1371/journal.pone.0140238 (2015).

299 Zinnhardt, B. et al. Molecular Imaging of Immune Cell Dynamics During De- and Remyelination in the Cuprizone Model of Multiple Sclerosis by [(18)F]DPA-714 PET and MRI. Theranostics 9, 1523-1537 (2019).

300 Ziser, L. et al. Utility of gradient recalled echo magnetic resonance imaging for the study of myelination in cuprizone mice treated with fingolimod. NMR in biomedicine 31 (2018).
