# Supplementary material for: Magnetic resonance imaging in multiple sclerosis animal models: A systematic review, meta-analysis, and white paper
Source: Neuroimage Clin. 2020 Aug 2;28:102371. doi: 10.1016/j.nicl.2020.102371 (PMC7451445; doi:10.1016/j.nicl.2020.102371)
Supplement: Supplementary data 3 [file mmc3.docx]

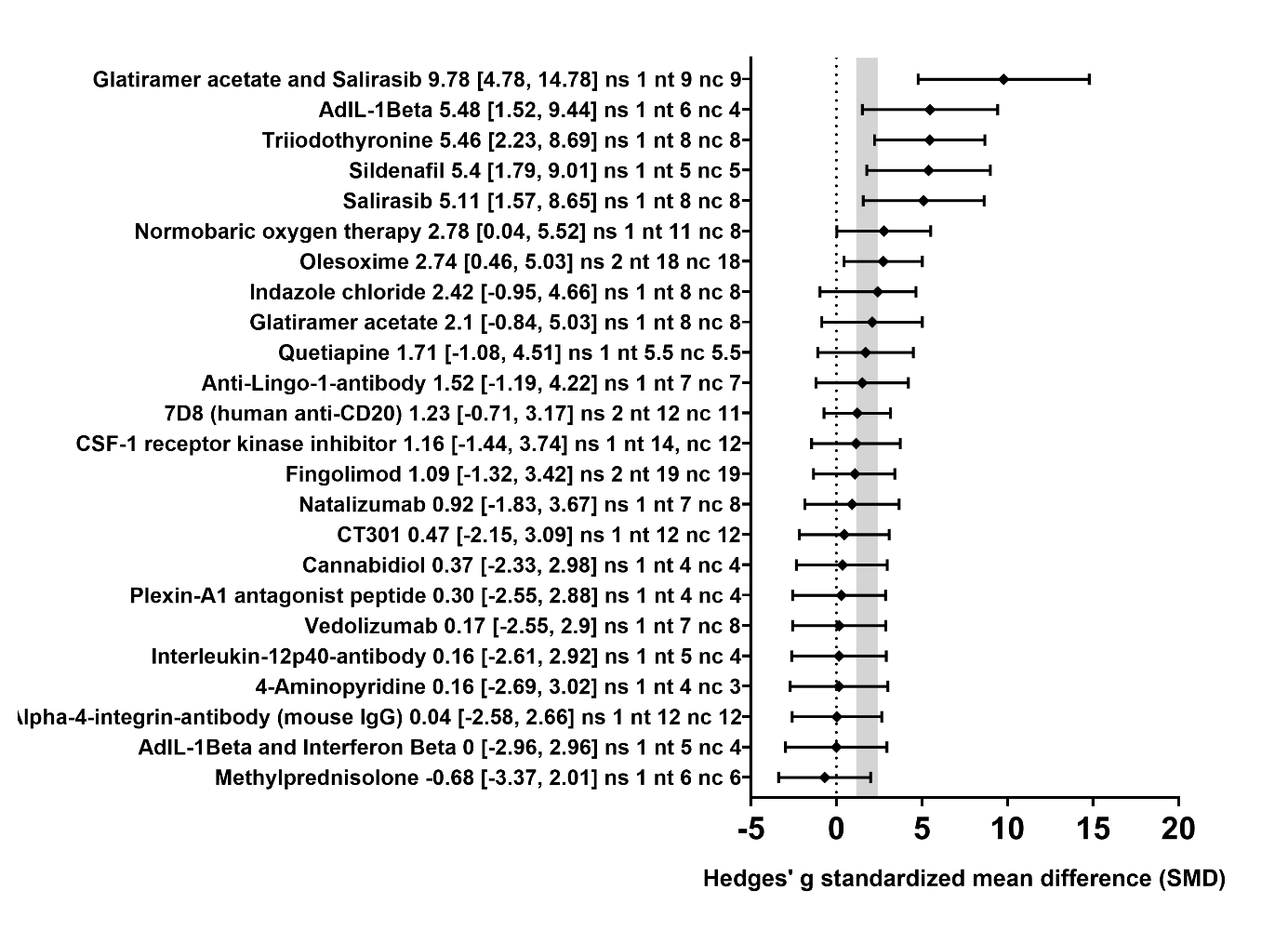


**Supplementary figure 1**: forest plot of the included studies for (re-)myelination outcomes. The diamond indicates the global estimate and the whiskers its 95% confidence interval (CI). The numbers listed after each therapy are: the exact effect size with its 95% CI, the number of included studies for a certain intervention (ns), the total number of treated animals (nt) and control animals (nc). The gray bar indicates the 95% CI of the overall effect size. References are given in the Supplementary information.


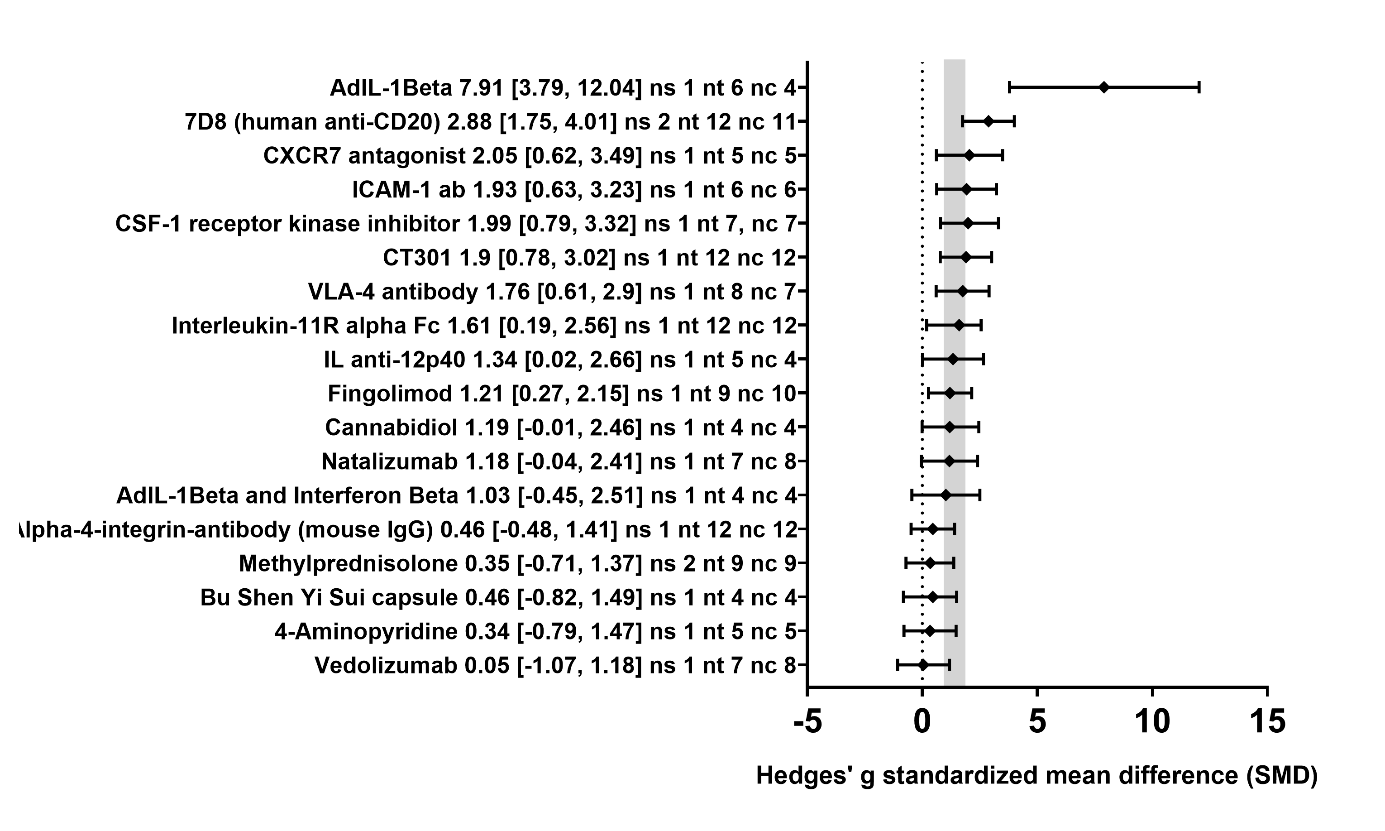


**Supplementary figure 2**: forest plot of the included studies for neuroinflammation outcomes. The diamond indicates the global estimate and the whiskers its 95% confidence interval (CI). The numbers listed after each therapy are: the exact effect size with its 95% CI, the number of included studies for a certain intervention (ns), the total number of treated animals (nt) and control animals (nc). The gray bar indicates the 95% CI of the overall effect size. References are given in the Supplementary information.


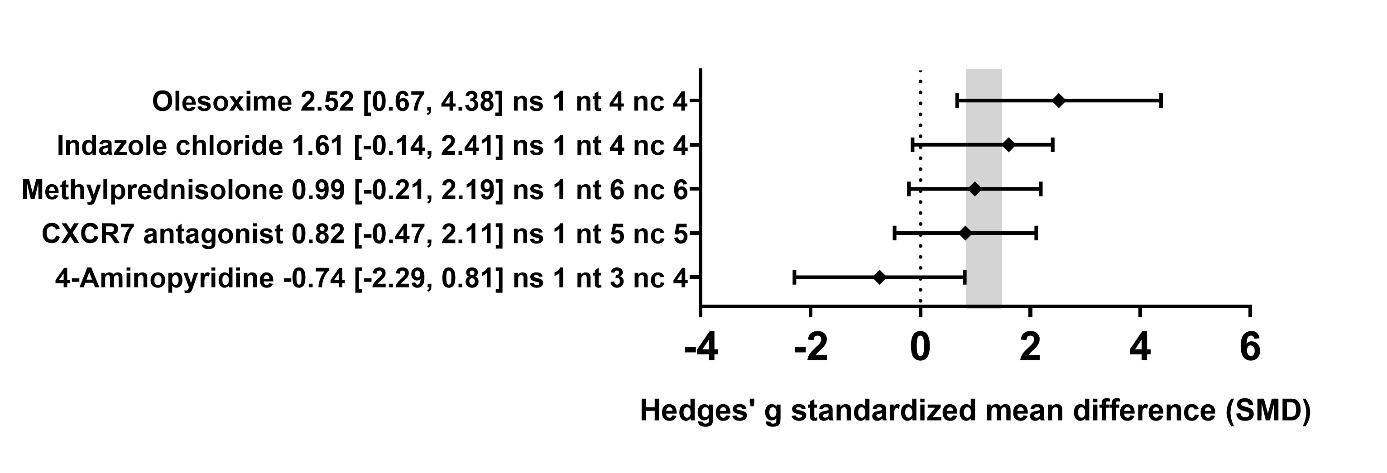


**Supplementary figure 3**: forest plot of the included studies for neurodegeneration outcomes. The diamond indicates the global estimate and the whiskers its 95% confidence interval (CI). The numbers listed after each therapy are: the exact effect size with its 95% CI, the number of included studies for a certain intervention (ns), the total number of treated animals (nt) and control animals (nc). The gray bar indicates the 95% CI of the overall effect size. References are given in the Supplementary information.
