# Supplementary material for: Magnetic resonance imaging in multiple sclerosis animal models: A systematic review, meta-analysis, and white paper
Source: Neuroimage Clin. 2020 Aug 2;28:102371. doi: 10.1016/j.nicl.2020.102371 (PMC7451445; doi:10.1016/j.nicl.2020.102371)
Supplement: Supplementary data 4 [file mmc4.docx]

| **First author** | **Year** | **Species** | **MS model** | **Therapy** | **MRI Supplier** | **B** | **MTR** | **Diffusion** | **Contrast medium** | **Gd-Dosage** | **Ex vivo/in vivo** | **region** |
| --- | --- | --- | --- | --- | --- | --- | --- | --- | --- | --- | --- | --- |
| Abakumova | 2015 | mice | Cuprizone | No | N/A | N/A |  |  |  |  | in vivo | brain |
| Abramowski | 2016 | mice | EAE | Mesenchymal stem cells | Bruker | 7 |  |  |  |  | in vivo | brain and spinal cord |
| Absinta | 2019 | marmoset | EAE | No | Bruker | 7 |  |  |  |  | ex vivo | brain |
| Acs | 2009 | mice | Cuprizone | 17Beta-Estradiol and Progesterone | Varian | 9.4 |  |  |  |  | in vivo | brain |
| Adler_1 | 2000 | rats | Chronic hyponatremia | No | Bruker | 4.7 |  |  | Gadoteridol | N/A | in vivo | brain |
| Adler_2 | 2000 | rats | Chronic hyponatremia | No | Bruker | 4.7 |  |  | Gadoteridol | N/A | in vivo | brain |
| Adler | 1995 | rats | Chronic hyponatremia | No | Bruker | 4.7 |  |  | Gd-DTPA | N/A | in vivo | brain |
| Aggarwal | 2012 | mice | Cuprizone | No | Bruker | 11.7 |  |  |  |  | ex vivo | brain |
| Aharoni | 2013 | mice | EAE | Glatiramer acetate* | Bruker | 9.4 | x |  |  |  | in vivo | brain |
| Ahrens | 1998 | mice | EAE | No | Bruker | 11.7 |  |  |  |  | ex vivo | spinal cord |
| Aizman | 2010 | mice | EAE | Glatiramer acetate and farnesylthiosalycylic acid* | Bruker | 7 |  |  | Gd-DTPA | 0.5 mmol/kg | in vivo | spinal cord |
| All | 2009 | rats | Targeted EAE | No | Bruker | 9.4 |  |  |  |  | in vivo | spinal cord |
| Anderson | 2004 | mice | EAE | No | Bruker | 7 |  |  | SPIO-PLL (T-cells labeled) | N/A | both | spinal cord |
| Anthony | 2014 | rats | Delayed Type Hypersensitivity | Fingolimod* | Varian | 7 |  |  | Gd-DTPA | N/A | in vivo | brain |
| Anthony | 2011 | rats | Targeted EAE | No | Varian | 7 |  |  |  |  | both | brain |
| Atkinson | 2018 | mice | Cuprizone | Indazole chloride (estrogen receptor β ligand)* | Bruker | 7 |  | 1 |  |  | both | brain |
| Axthelm | 2011 | macaques | Japanese macaque encephalomyelitis | No | Siemens | 3 |  |  | Gadoteridol | N/A | in vivo | brain and spinal cord |
| Badawi | 2012 | mice | EAE | Bifunctional peptide inhibitor* | Varian | 9.4 |  |  | Gd-DTPA | 0.6 mmol/kg | in vivo | brain |
| Baeten | 2010 | rats | EAE | No | Varian | 9.4 |  |  | SPIO-PLL-labelled T cells | N/A | ex vivo | brain and spinal cord |
| Baeten | 2008 | rats | EAE | No | Varian | 9.4 |  |  | USPIO | N/A | both | brain |
| Beckmann | 2018 | mice | Cuprizone and EAE | BLZ945 (CSF-1 receptor kinase inhibitor)* | Bruker | 7 | 1 |  |  |  | in vivo | brain |
| Bell | 2013 | mice | NMO | No | Bruker | 7 |  |  | Gd-DTPA | 0.2 mmol/kg | in vivo | brain and spinal cord |
| Belloli | 2018 | mice | EAE | No | Bruker | 7 |  |  | Gd-DTPA | 0.3 mmol/kg | in vivo | brain |
| Bendszus | 2008 | rats | EAE | No | Siemens | 1.5 |  |  | Gd-DTPA, Gadofluorine M | 0.1 mmol/kg, 0.2mmol/kg | in vivo | brain and spinal cord |
| Ben-Hur | 2007 | mice | EAE | No | Bruker | 4.7 |  |  | Ferridex and Poly-L-Lysine labelled Neurospheres |  | both | brain |
| Beraud | 2006 | rats | EAE | Kaliotoxin und Shk* | Bruker | N/A |  |  | Gd-DTPA | N/A | in vivo | brain |
| Berger | 2006 | rats | EAE | No | Bruker | 7 | x |  | Gd-DOTA, Sinerem | N/A | in vivo | brain |
| Biton | 2005 | swine | EAE | No | Bruker | 8.4 |  | x |  |  | ex vivo | spinal cord |
| Biname | 2019 | mice | Cuprizone and EAE | Plexin-A1 antagonist peptide* | Bruker | 7 |  |  |  |  | in vivo | brain |
| Bittner | 2009 | mice | EAE | Anandamide* | Siemens | 1.5 |  |  |  |  | in vivo | brain |
| Blair | 2016 | macaques | Japanese macaque encephalomyelitis | No | Siemens | 3 |  |  | Gadoteridol | N/A | in vivo | brain |
| Blezer | 2007 | marmoset | EAE | No | Bruker | 4.7 | x |  | Gd-DTPA | N/A | both | brain |
| Blezer | 2015 | mice | EAE | No | Varian | 9.4 |  |  | Gadobutrol, MPIO | N/A | in vivo | brain |
| Boretius | 2012 | mice | Cuprizone, EAE | No | Bruker | 2.35 | x |  | Gd-DTPA, MnCl2 | N/A | in vivo | brain |
| Bölcskei | 2018 | mice | Cuprizone | No | Bruker | 4.7 |  |  |  |  | in vivo | brain |
| Boretius | 2008 | rats | EAE | No | Bruker | 2.35 | x |  | Gd-DTPA, MnCl2 | 0.5 mmol/kg | in vivo | optic nerves |
| Boretius | 2006 | marmoset | EAE | No | Bruker | 2.35 |  |  | Gd-DTPA | 0.3 mmol/kg | in vivo | brain |
| Brochet | 2006 | macaques | EAE | No | N/A | 1.5 |  |  | Gd-DOTA | N/A | in vivo | brain and spinal cord |
| Brochet | 1999 | rats | EAE | No | Philipps | 1.5 |  |  | USPIO | N/A | in vivo | brain |
| Brok | 2002 | marmoset | EAE | Anti-IL-12p40 Monoclonal Antibody | Varian | 4.7 |  |  | Gd-DTPA | 0.3 mmol/kg | both | brain |
| Broom | 2005 | rats | Delayed Type Hypersensitivity | No | Varian | 7 |  | x | Gadodiamide | N/A | in vivo | brain |
| Budde | 2008 | mice | EAE | No | Varian | 4.7 |  | x |  |  | in vivo | spinal cord |
| Budde | 2009 | mice | EAE | No | Varian | 4.7 |  | x |  |  | in vivo | spinal cord |
| Bulte | 2003 | rats | EAE | No | Bruker | 4.7 |  |  | Cells labelled with MION-46L-OX-26 or MD-100 | N/A | ex vivo | brain |
| Cahill | 2019 | mice | EAE | No | Varian | 7 |  |  |  |  | ex vivo | brain |
| Cao | 2018 | mice | Cuprizone | No | Bruker | 7 | 1 |  |  |  | in vivo | brain |
| Carambia | 2015 | mice | EAE | No | N/A | N/A |  |  |  |  | in vivo | brain |
| Cate | 2010 | mice | Cuprizone | Methylprednisolone* | Bruker | 4.7 |  | x |  |  | in vivo | brain |
| Chandran | 2012 | mice | Cuprizone | Quetiapine* | Bruker | 7 |  | x |  |  | in vivo | brain |
| Chen_2 | 2008 | rats | EAE | No | Bruker | 4.7 |  | x | Gd-DTPA, Mg | N/A | in vivo | brain |
| Chen_1 | 2008 | mice | EAE | No | Bruker | 4.7 or 7 |  |  | Gd-DTPA | 0.3 mmol/kg | in vivo | brain |
| Chen | 2009 | mice | EAE | Minocycline and Methylprednisolone | GE | 1.5 |  |  | Gd-DTPA | 0.5 mmol/kg | in vivo | brain |
| Chen | 2010 | mice | EAE | Osthole | GE | 1.5 |  |  | Gd-DTPA | 0.5 mmol/kg | in vivo | brain |
| Chen | 2015 | macaques | Cuprizone | No | Siemens | 3 |  |  |  |  | in vivo | brain |
| Chin | 2009 | rats | EAE | No | Bruker | 7 |  |  | USPIO | N/A | in vivo | brain |
| Chu | 2019 | mice | Lysolecithin | No | Agilent | 9.4 |  | 1 |  |  | in vivo | spinal cord |
| Chuhutin | 2020 | mice | EAE | No | Bruker | 16.4 |  | 1 |  |  | ex vivo | brain and spinal cord |
| Cisneros | 2020 | rats | Ethidiu bromide | No | Bruker | 7 |  | 1 |  |  | in vivo | brain |
| Collongues | 2012 | rats | EAE | No | SMIS | 4.7 |  |  |  |  | in vivo | brain and spinal cord |
| Cook | 2005 | guinea pigs | EAE | No | GE | 1.5 |  |  |  |  | in vivo | spinal cord |
| Cook | 2004 | guinea pigs | EAE | No | Varian/Siemens | 4 | x |  |  |  | in vivo | spinal cord |
| Crombe | 2018 | mice | EAE | No | Bruker | 4.7 |  | 1 |  |  | in vivo and ex vivo | brain |
| Cruz-Orengo | 2011 | mice | EAE | CXCR7 antagonist* | Varian | 4.7 |  | x |  |  | in vivo | spinal cord |
| deSantanaNunes | 2016 | mice | Cuprizone | Sildenafil* | GE | 3 |  |  |  |  | in vivo | brain |
| DeBoy | 2007 | rats | Targeted EAE, Lysolecithin | No | Bruker | 11.7 |  | x |  |  | ex vivo | spinal cord |
| Degaonkar_1 | 2002 | rats | Lysolecithin | No | Bruker | 4.7 |  |  |  |  | in vivo | brain |
| Degaonkar | 2005 | rats | Lysolecithin | No | Bruker | 4.7 |  |  |  |  | in vivo | brain |
| Deloire | 2004 | rats | EAE | monoclonal TA2 VLA-4 antibody* | Philips | 1.5 |  |  | USPIO (AMI-227) | N/A | in vivo | brain and spinal cord |
| Deloire-Grassin | 2000 | rats | Lysolecithin | No | Bruker | 4.7 | x |  |  |  | in vivo | brain |
| Derdelinckx | 2019 | mice | EAE | No | Bruker | 7 |  |  |  |  | ex vivo | spinal cord |
| Desai | 2016 | rats | Lipopolysaccharide | Normobaric oxygen therapy* | Agilent | 9.4 |  |  |  |  | ex vivo | spinal cord |
| Diem | 2008 | marmoset | EAE | No | Bruker | 2.35 |  |  |  |  | in vivo | brain and optic nerves |
| Dommisse | 1991 | rats | EAE | No | Bruker or Sisco | 4.7, 1.9 |  |  |  |  | both | brain and spinal cord |
| Dousset | 1995 | macaques | Lysolecithin | No | N/A | 1.5 | x |  |  |  | in vivo | brain |
| Dousset | 1999 | rats | EAE | No | Bruker | 4.7 |  |  | USPIO (AMI-227) | N/A | in vivo | brain and spinal cord |
| Duckers | 1997 | rats | EAE | ACTH analogue* | SISCO | 4.7 |  |  |  |  | in vivo | brain |
| Elo | 2018 | rats | EAE | No | Philips | 3 |  |  | Gd-DTPA | 279.3 mg/ml | in vivo | brain |
| Elo | 2019 | rats | EAE | No | Philips | 3 |  |  | Gd-DTPA | 279.3 mg/ml | in vivo | brain |
| Engberink | 2010 | rats | EAE | No | Varian | 4.7 |  |  | USPIO-labeled  Monocytes  (Sinerem, Endorem,  Supravist) | N/A | both | brain and spinal cord |
| Esposito | 2013 | mice | EAE | No | Bruker | 7 |  |  |  |  | in vivo | brain and spinal cord |
| Falangola | 2014 | mice | Cuprizone | No | Agilent | 7 |  | x |  |  | in vivo | brain |
| Fang | 2013 | rats | EAE | αυβ3 integrin-binding peptide* | GE | 3 |  |  |  |  | ex vivo | brain |
| Fjaer | 2013 | mice | Cuprizone | No | Bruker | 7 | x |  |  |  | in vivo | brain |
| Fjaer | 2015 | mice | EAE | No | Bruker | 7 | x |  |  |  | in vivo | brain |
| Floris | 2004 | rats | EAE | No | Varian | 4.7 |  |  | Gd-DTPA, USPIO 7228 | N/A | in vivo | brain |
| Ford | 1990 | rats | Lysolecithin | No | GE | 2 |  |  | Gd-DOTA | 200-400 umol/kg | in vivo | brain |
| Fournier | 2017 | mice | EAE | No |  | N/A |  |  | MPIO | N/A | in vivo | brain |
| Fournier | 2019 | mice | EAE | No | Bruker | 7 |  |  |  |  | in vivo | brain |
| Gadjanski | 2009 | rats | EAE | W-conotoxin* | Bruker | 2.35 |  |  | MnCl2 | N/A | in vivo | optic nerves |
| Gaitan | 2014 | marmoset | EAE | No | Bruker | 7 |  |  |  |  | both | brain |
| Gareau | 2000 | guinea pigs | EAE | No | Varian/Siemens | 4 |  |  |  |  | in vivo | brain |
| Gareau | 2002 | guinea pigs | EAE | No | N/A | 9.4 |  |  |  |  | ex vivo | brain |
| Gilli | 2016 | mice | TMEV | No | N/A | N/A |  | x |  |  | in vivo | spinal cord |
| Gobel | 2013 | mice | EAE | 4-Aminopyridine* | Philipps | 3 |  |  |  |  | in vivo | brain |
| Gonzalez-Garcia | 2017 | mice | EAE* | Cannabidiol | Bruker | 7 |  |  |  |  | in vivo | brain |
| Grossman | 1987 | guinea pigs | EAE | No | N/A | 1.4 |  |  |  |  | in vivo | brain |
| Guglielmetti_1 | 2016 | mice | Cuprizone | No | Bruker | 9.4 | x | x |  |  | in vivo | brain |
| Guglielmetti_2 | 2016 | mice | Cuprizone | No | Bruker | 9.4 |  | x |  |  | in vivo | brain |
| Guglielmetti | 2017 | mice | Cuprizone | No | Agilent | 14.1 |  |  | 13C-pyruvate | N/A | in vivo | brain |
| Guglielmetti | 2020 | mice | Cuprizone | No | GE | 7 |  |  |  |  | in vivo | brain |
| Guy | 1994 | guinea pigs | EAE | No | N/A | 2 |  |  | Gd-DTPA | 0.2 mmol/kg | in vivo | optic nerves |
| Guy | 2016 | marmoset | EAE | No | Bruker | 7 |  |  |  |  | both | brain |
| Haanstra | 2013 | macaques | EAE | Natalizumab and Vedolizumab* | Varian | 9.4 | x |  |  |  | ex vivo | brain |
| Hamilton | 2019 | mice | EAE | No | Bruker | 9.4 |  |  |  |  | in vivo | brain |
| Hao | 2011 | mice | EAE | Nicotine Bitartrate* | Bruker | 7 |  |  |  |  | in vivo | brain and spinal cord |
| Harsan | 2008 | mice | Cuprizone | Triiodothyronine* | Rapid Biomedical | 4.7 |  | x |  |  | in vivo | brain |
| Hart | 1998 | marmoset | EAE | No | Varian | N/A |  |  | Gd-DTPA | N/A | both | brain |
| Hawkins | 1990 | guinea pigs | EAE | No | N/A | N/A |  |  | Gd-DTPA | 0.25 mmol/kg | in vivo | spinal cord |
| Hawkins | 1992 | guinea pigs | EAE | No | Picker | N/A |  |  | Gd-DTPA | 0.25-0.5 mmol/kg | in vivo | spinal cord |
| Heckl | 2004 | rats | EAE | No | Siemens | 3 |  |  | Gd-DTPA | 0.5 mmol/kg | in vivo | brain and spinal cord |
| Heide | 1993 | macaques | EAE | No | GE | 2 |  | x |  |  | in vivo | brain |
| Helms | 2013 | marmoset | Targeted EAE | No | Siemens | 3 |  |  | Gadobutrol | 0.3 mmol/kg | in vivo | brain |
| Herrera | 2014 | mice | EAE | No | Bruker | 7 |  | x |  |  | in vivo | brain and optic nerves |
| Hoffmann | 2013 | rats | EAE | No | Bruker | 9.4 |  |  | MnCl2 | N/A | in vivo | optic nerves |
| Hubner | 2017 | mice | Cuprizone | No | Bruker | 7 |  | 1 |  |  | in vivo | brain |
| Hunger | 2014 | rats | EAE | No | Bruker | 4.7 | x |  |  |  | in vivo | brain |
| Jagessar | 2012 | marmoset | EAE | Anti-CD20-antibody (7D8)* | Varian | 9.4 |  |  |  |  | ex vivo | brain |
| Jagessar | 2010 | marmoset | EAE | No | Varian | 4.7 |  |  |  |  | both | brain |
| Jagessar | 2008 | marmoset | EAE | No | Varian | 4.7 |  |  |  |  | ex vivo | brain |
| Jaini | 2013 | mice | EAE | No | Bruker | 7 |  |  |  |  | in vivo | brain |
| Janve | 2013 | rats | Lipopolysaccharide | No | Agilent/Varian | 9.4 |  | x |  |  | ex vivo | brain |
| Jelescu | 2016 | mice | Cuprizone | No | Bruker | 7 | x | x |  |  | in vivo | brain |
| Jiang | 2013 | mice | EAE | Donepezil | GE | 1.5 |  |  | Gd-DTPA | 0.5 mmol/kg | in vivo | brain |
| Johnson | 2014 | mice | EAE | No | Bruker | 7 |  |  | Gadolinium | 100 mg/kg | in vivo | brain |
| Jordan | 1999 | marmoset | EAE | No | GE | 1.5 | x |  | Gd-DTPA | N/A | in vivo | brain |
| Kap | 2011 | marmoset | EAE | Anti-CD20-antibody (7D8)* | Varian | 4.7 | x |  | Gadobutrol, Gd-BT-DO3A | N/A | both | brain |
| Kap | 2010 | marmoset | EAE | Anti-CD20-antibody (7D8)* | Varian | 9.4 | x |  |  |  | ex vivo | brain |
| Karlik | 1993 | guinea pigs | EAE | No | Bruker | 1.89 |  |  | Gd-DTPA | N/A | in vivo | brain |
| Karlik | 1990 | guinea pigs | EAE | No | N/A | N/A |  |  |  |  | ex vivo | spinal cord |
| Karlik | 1999 | rats | EAE | PAL68–86 | GE | 1.5 |  |  | Gd-DTPA | 1 mM/kg | in vivo | brain |
| Karlik | 1986 | guinea pigs | EAE | No | N/A | N/A |  |  |  |  | ex vivo | brain and spinal cord |
| Kent | 1995 | guinea pigs | EAE | Anti-a4 subunit of a4pl integrin* | GE | 1.5 |  |  | Gd-DTPA | 1 mM/kg | in vivo | brain |
| Khodanovich | 2016 | mice | Cuprizone | Ropren* | Bruker | 11.7 |  |  |  |  | in vivo | brain |
| Khodanovich | 2017 | mice | Cuprizone | No | Bruker | 11.4 | 1 |  |  |  | in vivo | brain |
| Kim_1 | 2012 | mice | EAE | Human glial precursor cell | Bruker | 9.4 |  |  |  |  | both | brain |
| Kim_2 | 2012 | mice | EAE | Human glial precursor cell | Bruker | 9.4 |  |  |  |  | both | brain |
| Kim | 2006 | mice | EAE | No | Varian | 4.7 |  | x |  |  | in vivo | spinal cord |
| Kirschbaum | 2016 | mice | EAE | No | Bruker | 9.4 |  |  | Gadodiamide, ferumoxytol, CLIO | N/A | in vivo | brain |
| Krauspe | 2015 | mice | Cuprizone | No | Bruker | 7 |  |  |  |  | in vivo | brain |
| Kriszta | 2019 | mice | Cuprizone | No | Bruker | 4.7 |  |  |  |  | in vivo | brain |
| Kuharik | 1988 | dogs | EAE | No | Picker | 1.5 |  |  | Gd-DTPA | 0.2 mmol/kg | in vivo | brain |
| Ladewig | 2009 | rats | EAE | No | Siemens | 1.5 |  |  |  |  | in vivo | brain |
| Laman | 2002 | marmoset | EAE | Anti-CD40 antibody* | Varian | N/A |  |  | Gd-DTPA | 0.3 mmol/kg | in vivo | brain |
| Le Blon | 2016 | mice | Cuprizone | No | Bruker | 9.4 |  |  |  |  | in vivo | brain |
| Lee | 2012 | mice | Cuprizone | No | Bruker | 7 |  |  |  |  | both | brain |
| Lee | 2018 | marmoset | EAE | No | Bruker | 7 |  |  | Gadobutrol | 0.3 mg/kg | in vivo | brain |
| Lefeuvre | 2020 | marmoset | EAE | No | Bruker | 7 |  |  |  |  | ex vivo | spinal cord |
| Levy Barazany | 2014 | mice | EAE | Nasal MOG* | Bruker | 7 |  | x | Gd-DTPA | 0.5 mmol/kg | in vivo | brain |
| Levy | 2010 | mice | EAE | No | Bruker | 7 |  | x | Gd-DTPA | 0.5 mmol/kg | in vivo | brain |
| Li | 2013 | rats | Cuprizone | TRO19622* | GE | 7 |  |  |  |  | in vivo | brain |
| Li | 2019 | mice | EAE | No | Bruker | 4.7 |  |  | Myeliperoxidase-Gd | 0.3 mmol/kg | in vivo | brain |
| Li-ChunHsieh | 2015 | mice | EAE | Gelsolin* | Bruker | 4.7 |  |  | MPO-Gad | 0.3 mmol/kg | in vivo | brain |
| Lin_1 | 2014 | mice | EAE | No | Agilent | 4.7 |  |  | MnCl2 | N/A | in vivo | brain and optic nerves |
| Lin_2 | 2014 | mice | EAE | No | Agilent | 4.7 |  | x |  |  | in vivo | brain |
| Lin | 2017 | mice | EAE | No | Agilent | 4.7 |  | 1 |  |  | in vivo | brain |
| Linker | 2006 | rats | EAE | No | Siemens | 1.5 |  |  | SPIO | N/A | in vivo | brain |
| Linker | 2005 | rats | EAE | No | Siemens | 1.5 |  |  | Gd-DTPA | 0.2 mmol/kg | in vivo | brain |
| Liu | 2019 | mice | EAE | No | Bruker | 11.7 |  | 1 | Gd-DTPA | N/A | in vivo | brain |
| Luo | 2019 | mice | Lysolecithin | No | Bruker | 9.4 |  | 1 |  |  | in vivo | brain |
| Lodygensky | 2010 | rats | Lipopolysaccharide | No | Varian | 11.7 |  |  |  |  | in vivo | brain |
| Luchetti | 2012 | mice | EAE | No | Siemens | 3 |  |  | Gd-DTPA | N/A | in vivo | brain |
| MacKenzieGraham_1 | 2006 | mice | EAE | No | Bruker | 7 |  |  |  |  | both | brain |
| MacKenzieGraham_2 | 2006 | mice | EAE | No | Bruker | 11.7 |  |  |  |  | ex vivo | brain |
| MacKenzieGraham | 2009 | mice | EAE | No | Bruker | 11.7 |  |  |  |  | ex vivo | brain |
| Magalon | 2012 | mice | Cuprizone, Lysolecithin | Olesoxime* | Bruker | 11.8 |  |  |  |  | in vivo | brain |
| Maggi | 2014 | marmoset | EAE | No | Bruker | N/A |  |  | Gd-DTPA | N/A | both | brain |
| Manogaran | 2018 | mice | EAE | No | Bruker | 7 |  | 1 |  |  | in vivo | brain |
| Mardiguian | 2013 | mice | EAE | Anti IL-17A antibody* | Varian | 7 |  |  | Gd-DTPA, MPIO | N/A | in vivo | brain |
| Marriott | 2008 | mice | Cuprizone | Leukemia inhibiting factor* | Bruker | 4.7 |  |  |  |  | in vivo | brain |
| Masthoff | 2018 | mice | EAE | No | Bruker | 9.4 |  |  | Ferucarbotran/Resovist | 1.3 ml/kg | in vivo | brain |
| McAteer | 2007 | mice | IL-1Beta | No | Varian | 7 |  |  | MPIO |  | in vivo | brain |
| McAteer | 2011 | mice | IL-1Beta | No | Varian | 7 |  |  |  |  | in vivo | brain |
| McCreary | 2009 | mice | Lysolecithin | No | Bruker | 9.4 | x |  |  |  | in vivo | spinal cord |
| Merkler | 2005 | mice | Cuprizone | No | Bruker | 2.35 | x |  |  |  | in vivo | brain |
| Mi | 2007 | rats | EAE | Anti-Lingo-1 antibody* | Bruker | 7 |  | x |  |  | ex vivo | spinal cord |
| Miao | 2019 | mice | EAE | No | Bruker |  |  | 1 |  |  | in vivo | brain |
| Mikita | 2011 | rats | EAE | No | Bruker | 4.7 |  |  | USPIO labeled macrophages | N/A | in vivo | brain |
| Millward | 2013 | mice | EAE | No | Bruker | 7 |  |  | Gd-DTPA, VSPO | 0.2 mmol/kg | in vivo | brain |
| Millward | 2019 | mice | EAE | No | Bruker | 7 |  |  | Gd-DTPA | 0.2 mmol/kg | in vivo | brain |
| Modica | 2017 | mice | TMEV | No | Bruker | 9.4 |  |  |  |  | in vivo | brain |
| Moon | 2015 | dogs | EAE | No | Hitachi | 0.4 |  |  | Gadodiamide | 0.3 ml/kg | in vivo | brain |
| Morrissey_1 | 1996 | rats | EAE | Anti-ICAM-1 antibody* | Bruker | 7 |  |  | Gd-DTPA | 0.8 mmol/kg | in vivo | brain |
| Morrissey_2 | 1996 | rats | EAE | No | Bruker | 7 |  |  | Gd-DTPA | 0.8 mmol/kg | in vivo | brain |
| Muja | 2011 | mice | EAE | No | Bruker | 9.4 |  |  | SPIO-labeled NPCs |  | both | brain |
| Namaer | 1992 | rats | EAE | No | Bruker | 4.7 |  |  | Gd-DOTA | 0.2-0.5 mmol/kg | in vivo | brain |
| Namer | 1998 | rats | EAE | No | Bruker | 4.7 |  |  | Gd-DOTA | N/A | in vivo | brain |
| Namer_1 | 1994 | rats | EAE | No | Bruker | 4.7 |  |  | Gd-DOTA | N/A | in vivo | brain |
| Namer | 1993 | rats | EAE | No | Bruker | 4.7 |  |  | Gd-DOTA | N/A | in vivo | brain |
| Namer_2 | 1994 | rats | EAE | Hyperbaric oxygen treatment* | Bruker | 4.7 |  |  | Gd-DOTA | N/A | in vivo | brain |
| Nathoo | 2013 | mice | EAE | No | Bruker | 9.4 |  |  |  |  | both | brain and spinal cord |
| Nathoo | 2015 | mice | EAE | No | Bruker | 9.4 |  |  |  |  | both | spinal cord |
| Nessler | 2007 | mice | EAE | No | Bruker | 2.35 |  |  | Gd-DTPA | N/A | in vivo | brain |
| Nie | 2014 | mice | Cuprizone | No | Agilent | 7 |  | x |  |  | in vivo | brain |
| Nishioka | 2019 | mice | EAE | No | Bruker | 11.4 |  | 1 |  |  | in vivo | brain |
| Nishioka | 2017 | mice | EAE | No | Bruker | 11.4 |  |  |  |  | in vivo | brain |
| Noseworthy | 1988 | guinea pigs | EAE | No | GE | 1.5 |  |  |  |  | in vivo | brain |
| Noth | 1997 | rats | EAE | No | Bruker | 7.05 |  |  | Gd-DTPA, labeled PFC | N/A | in vivo | brain |
| Oakden | 2017 | rats | Cuprizone | No | Bruker | 7 | 1 |  |  |  | in vivo | brain |
| Orije | 2015 | mice | Cuprizone | No | Bruker | 9.4 |  |  |  |  | in vivo | brain |
| OudeEngberink | 2010 | rats | EAE | No | Varian | 4.7 |  |  | Gd-DTPA, USPIO | N/A | in vivo | brain |
| Oweida | 2004 | rats | EAE | No | GE | 1.5 |  |  | Gd-DTPA, Feridex | N/A | both | brain |
| Oweida | 2007 | mice | EAE | No | GE | 1.5 |  |  | Feridex | N/A | in vivo | brain |
| Palazuelos | 2008 | mice | EAE | CB2 agonist | Bruker | 4.7 |  |  |  |  | in vivo | brain and spinal cord |
| PazSoldan | 2015 | mice | TMEV | No | Bruker | 7 |  |  |  |  | in vivo | brain |
| Peersman | 1988 | guinea pigs | Chronic EAE | No | Bruker | 4.7 |  |  |  |  | ex vivo | spinal cord |
| Petiet | 2016 | mice | Cuprizone | No | Bruker | 11.7 |  | x |  |  | in vivo | brain |
| Pettersson | 2004 | rats | EAE | No | Bruker | 4.7 |  |  |  |  | in vivo | brain and spinal cord |
| Piraino | 2005 | guinea pigs | EAE | Anti-a4integrin antibody* | GE | 1.5 |  |  | Gd-DTPA | N/A | in vivo | brain |
| Pirko_1 | 2004 | mice | TMEV | rHIgM22 | Bruker | 7 |  |  |  |  | in vivo | brain and spinal cord |
| Pirko | 2003 | mice | EAE | No | Bruker | 7 |  |  | supraparamagnetically labeled antibodies against CD4 | N/A | in vivo | brain and spinal cord |
| Pirko_1 | 2012 | mice | TMEV | No | Bruker | 7 |  |  |  |  | in vivo | brain |
| Pirko_2 | 2012 | mice | TMEV | No | Bruker | 7 |  |  |  |  | in vivo | brain |
| Pirko_2 | 2004 | mice | TMEV | No | Bruker | 7 |  |  | Gd-DTPA | N/A | in vivo | brain |
| Pirko | 2011 | mice | TMEV | No | Bruker | 7 |  |  |  |  | in vivo | brain |
| Pirko | 2009 | mice | TMEV | No | Bruker | 7 |  |  |  |  | in vivo | brain |
| Pirko_3 | 2004 | mice | TMEV, EAE | No | Bruker | 7 |  |  | supraparamagnetic anti-CD4, CD8, and Mac1-antibodies | N/A | in vivo | brain and spinal cord |
| Pirko_4 | 2004 | mice | TMEV | No | Bruker | 7 |  |  |  |  | in vivo | brain and spinal cord |
| Pol_1 | 2019 | mice | EAE | No | Bruker | 9.4 |  |  |  |  | in vivo | brain |
| Pol_2 | 2019 | mice | TMEV | Teriflunomide | Bruker | 9.4 |  | 1 |  |  | in vivo | brain |
| Politi | 2007 | mice | EAE | No | Philips | 3 |  |  | Gd-DTPA, resovist-labeled cells | 0.6 ml/kg | in vivo | brain |
| Praet | 2015 | mice | Cuprizone | No | Bruker | 9.4 |  |  |  |  | in vivo | brain |
| Qi_1 | 2007 | mice | EAE | No | Oxford instruments limited | 4.7 |  |  | Gd-DTPA | 0.2 mmol/kg | in vivo | brain and spinal cord |
| Qi_2 | 2007 | mice | EAE | No | Oxford instruments limited | 4.7 |  |  | Gd-DTPA | 0.2 mmol/kg | in vivo | brain and spinal cord |
| Rausch | 2003 | rats | EAE | No | N/A |  | x |  | Gd-DOTA, USPIO (AMI-227) | N/A | in vivo | brain |
| Rausch | 2004 | rats | EAE | Fingolimod* | Bruker | 7 |  |  | Gd-DOTA, USPIO (AMI-227) | N/A | in vivo | brain |
| Rausch | 2009 | rats | N/A | No | Bruker | 7 | x |  |  |  | in vivo | brain |
| Richards | 1995 | macaques | EAE | No | Bruker/GE | 4.7 |  |  |  |  | in vivo | brain |
| Robinson | 2010 | mice | EAE | No | Bruker | 11.8 |  |  | Feridex | N/A | ex vivo | spinal cord |
| Rose | 1989 | macaques | EAE | No | GE | 2 |  |  |  |  | in vivo | brain |
| Rose | 1997 | macaques | EAE | Anti-CD-18 and Dexamethason* | GE/Bruker | 2, 4.7 |  |  |  |  | in vivo | brain |
| SaskiaHubner | 2017 | mice | Cuprizone | No | Bruker | 7 |  | x |  |  | in vivo | brain |
| Serguera | 2019 | macaques | EAE | No | Agilent | 7 |  |  |  |  | in vivo | brain |
| Schellenberg | 2012 | mice | EAE | No | Bruker | 7 |  |  | Gd-DTPA | N/A | in vivo | spinal cord |
| Schneider | 2009 | rats | EAE | No | Siemens | 1.5 |  |  | SPIO | N/A | both | spinal cord |
| Seeldrayers | 1993 | rats | EAE | No | Bruker | 4.7 |  |  | SPIO | 0.8 mmol/kg | in vivo | brain |
| Serres | 2009 | rats | Targeted EAE | No | Varian | 7 | x | x | Gadodiamide, USPIO | N/A | both | brain |
| Serres | 2013 | rats | Targeted EAE | Interferon Beta and adenovirus expressing IL-1β* | Varian | 7 | x | x | Gd-DOTA | N/A | in vivo | brain |
| Serres | 2011 | mice | EAE | No | Varian | 7 |  |  | Gd-DTPA, VCAM-ab coupled MPIO | N/A | in vivo | brain |
| Sibson | 2004 | rats | Cytokine injection to brain | No | Varian | 7 |  |  | Gd-DTPA | N/A | in vivo | brain |
| Silva | 2018 | rats | Interleukin overexpression | No | Philips | 3 |  |  | Dotarem |  | in vivo | brain |
| Singer | 2000 | minipigs | EAE | No | GE | 1.5 |  |  | Gd-DTPA | 0.3 mmol/kg | in vivo | brain |
| Sipkins | 2000 | mice | EAE | No | GE | 9.4 |  |  | Antibody-conjugated paramagnetic liposome-Gd | 1.2 mg Gd/kg bw | ex vivo | brain |
| Smith | 2018 | mice | EAE | Fingolimod, Teriflunomide or Anti-IL-17A* | Bruker | 7 | 1 |  |  |  | in vivo | brain |
| Song | 2005 | mice | Cuprizone | No | Oxford instruments limited | 4.7 |  | x |  |  | ex vivo | brain |
| Soustelle | 2019 | mice | Cuprizone | No | Bruker | 7 | 1 | 1 |  |  | ex vivo | brain |
| Stassart | 2016 | marmoset | Targeted EAE | No | Siemens | 3 |  |  | Gd-DTPA | 0.3 mmol/kg | in vivo | brain |
| Steinbrecher | 2005 | rats | EAE | No | Bruker | 17.6 |  |  |  |  | ex vivo | spinal cord |
| Stewart | 1991 | macaques | EAE | No | N/A | 0.15 |  |  |  |  | in vivo | brain |
| Stewart | 1985 | macaques | EAE | No | Picker International | 0.15 |  |  |  |  | in vivo | brain |
| Stoll | 2004 | rats | EAE | No | Siemens | 1.5 |  |  | SPIO | N/A | in vivo | spinal cord |
| Sun | 2007 | mice | Cuprizone | No | Oxford Instruments/INOVA | 4.7 |  | x |  |  | in vivo | brain and optic nerves |
| Sun | 2006 | mice | EAE | No | Oxford Instruments/INOVA | 4.7 |  | x |  |  | in vivo | brain |
| t Hart_1 | 2005 | marmoset | EAE | Anti-ch5D12 antibody* | Varian | 4.7 |  |  | Gd-DTPA | N/A | in vivo | brain |
| t Hart_2 | 2005 | marmoset | EAE | IL anti-12p40* | Varian | 4.7 |  |  | Gd-DTPA | N/A | in vivo | brain |
| t Hart | 1998 | marmoset | EAE | No | Varian | 4.7 |  |  | Gd-DTPA | N/A | both | brain |
| Tanikawa | 2020 | mice | EAE | No | Bruker | 11.7 |  |  |  |  | in vivo | brain |
| Tagge | 2016 | mice | Cuprizone | No | Bruker | 11.8 | x |  |  |  | in vivo | brain |
| Talbott | 2016 | rats | Ethidium bromide | No | Varian | 7 |  | x |  |  | in vivo | spinal cord |
| Talla | 2013 | mice | ON | No | Oxford instruments limited | 4.7 |  |  |  |  | in vivo | optic nerves |
| Tambalo | 2015 | rats | EAE | No | Bruker | 4.7 |  |  | Superparamagnetic iron-oxide blood pool contrast agent (Endorem) | N/A | in vivo | brain |
| Thiessen | 2013 | mice | Cuprizone | No | Bruker | 7 | x | x |  |  | both | brain |
| Tobin | 2011 | mice | Cuprizone | No | Oxford instruments limited | 4.7 |  | x |  |  | in vivo | brain |
| Torkildsen | 2009 | mice | Cuprizone | PUFA diet* | Bruker | 7 |  |  |  |  | in vivo | brain |
| Tourdias_1 | 2011 | rats | Targeted EAE | No | Bruker | 4.7 |  | x | Gadodiamide | 0.2 mmol/kg | in vivo | brain |
| Tourdias_2 | 2011 | rats | Lysolecithin | No | Philipps | 1.5 |  | x |  |  | in vivo | brain |
| Turati | 2015 | mice | Cuprizone | No | Bruker | 7 | x |  |  |  | in vivo | brain |
| Tysiak | 2009 | mice | EAE | No | Bruker | N/A |  |  | Gd-DTPA, VSOP | 0.2 mmol/kg | in vivo | brain |
| VanLambalgen | 1987 | rhesus monkeys | EAE | Anti-OKT4/8 antibody* | Technicare | 0.6 |  |  |  |  | in vivo | brain |
| Varga | 2018 | mice | Cuprizone | No | Siemens | 3 |  |  |  |  | in vivo | brain |
| Verhoye | 1996 | rats | EAE | No | SMIS | 7 |  | x |  |  | in vivo | brain |
| Vowinckel | 1997 | mice | EAE | No | Philips | 1.5 |  |  |  |  | in vivo | brain |
| Waiczies | 2013 | mice | EAE | No | Bruker | 9.4 |  |  |  |  | in vivo | brain |
| Waiczies | 2012 | mice | EAE | No | Bruker | 9.4 |  |  | Gd-DTPA | 0.2 mmol/kg | in vivo | brain |
| Waiczies | 2017 | mice | EAE | No | Bruker | 9.4 |  |  |  |  | ex vivo | brain |
| Waiczies | 2019 | mice | EAE | No | Bruker | 9.4 |  |  |  |  | ex vivo | brain |
| Wang | 2013 | mice | EAE | Fingolimod* | Oxford Instruments/Agilent/Varian | 4.7 |  | x |  |  | in vivo | spinal cord |
| Wang | 2014 | mice | EAE | Lenaldekar* | Agilent | 4.7 |  | x |  |  | ex vivo | spinal cord |
| Wang | 2011 | mice | Cuprizone | No | Varian | 4.7 |  | x |  |  | in vivo | brain |
| Wang_1 | 2019 | mice | Cuprizone | No | Agilent | 9.4 |  | 1 |  |  | ex vivo | brain |
| Wang_2 | 2019 | mice | Cuprizone | No | Bruker | 7 |  | 1 |  |  | in vivo and ex vivo | brain |
| Williams | 2011 | mice | EAE, targeted EAE | No | Varian | 9.4 |  |  |  |  | in vivo | brain |
| Wood | 2016 | mice | Cuprizone | No | Agilent | 7 |  | x |  |  | ex vivo | brain |
| Wu | 2008 | mice | Cuprizone | No | Bruker | 4.7 |  | x |  |  | in vivo | brain |
| Wuerfel | 2010 | mice | EAE | No | Bruker | 7 |  |  | Gd-DTPA, gadofluorine | 0.2 mmol/kg, 0.1 mmol/kg | in vivo | brain |
| Wuerfel | 2007 | mice | EAE | No | Bruker | 7 |  |  | Gd-DTPA, gadofluorine, VSOP | 0.2 mmol/kg, 0.1 mmol/kg | in vivo | brain |
| Xiao | 2004 | rats | EAE | IFN-gamma-primed dendritic cells | Bruker | N/A |  |  |  |  | in vivo | brain and spinal cord |
| Xu_1 | 1998 | mice | EAE | No | GE | 4.7 |  |  | MION-46L | N/A | in vivo | brain |
| Xu_2 | 1998 | mice | EAE | Insulin-like growth factor 1* | GE | 4.7 |  |  |  |  | in vivo | brain |
| Yano | 2018 | mice | Cuprizone | No | Bruker | 7 |  | 1 |  |  | ex vivo | brain |
| Yu | 2004 | mice | Cuprizone | No | SMIS | 4.7 |  |  |  |  | in vivo | brain |
| Zaaraoui | 2008 | mice | Cuprizone | No | Bruker | 9.4 | x |  |  |  | in vivo | brain |
| Zhang | 2012 | mice | Cuprizone | No | Bruker | 9.4 | x | x |  |  | both | brain |
| Zhang | 2006 | mice | EAE | No | Bruker | 7 |  |  | Ferumoxytol-USPIO | N/A | in vivo | spinal cord |
| Zhang | 2014 | mice | EAE | No | Bruker | 7 |  |  | Ferumoxytol-USPIO | N/A | in vivo | spinal cord |
| Zhang | 2018 | mice | EAE | IL-11Rα Fc* | Bruker | 9.4 |  | 1 |  |  | in vivo | brain |
| Zhang | 2019 | mice | EAE | No | Bruker | 4.7 |  |  | Myeloperoxidase-Gd | N/A | in vivo | brain |
| Zhao | 2018 | mice | EAE | Bu Shen Yi Sui capsule or prednisone* | Bruker | 7 |  | 1 |  |  | in vivo | brain |
| Zhong | 2015 | rats | EAE | Cyclophosphamid* | Bruker | 7 |  |  | PFC nanoemulsion | N/A | both | spinal cord |
| Zinnhardt | 2019 | mice | Cuprizone | No | Bruker | 9.4 |  |  |  |  | in vivo | brain |
| Ziser | 2018 | mice | Cuprizone | Fingolimod* | Bruker | 9.4 |  |  |  |  | on vivo and ex vivo | brain |

**Supplementary table 1: included publications with additional information on experimental setup.**

Asterisks in the therapy column indicate therapeutic approaches included to the meta-analysis. Abbreviations: B, magnetic field strength of magnetic resonance scanner (Tesla); EAE, experimental autoimmune encephalomyelitis; Gd, gadolinium; IL, interleukin; MPIO, micrometer-sized paramagnetic iron oxide; NMO, neuromyelitis optica; PFC, perfluorocarbon; SPIO, superparamagnetic iron oxide; USPIO, ultrasmall superparamagnetic iron oxides; VSPO, very small iron oxide nanoparticles.
