# Supplementary material for: Magnetic resonance imaging in multiple sclerosis animal models: A systematic review, meta-analysis, and white paper
Source: Neuroimage Clin. 2020 Aug 2;28:102371. doi: 10.1016/j.nicl.2020.102371 (PMC7451445; doi:10.1016/j.nicl.2020.102371)
Supplement: Supplementary data 5 [file mmc5.docx]

**Supplementary table 2:** Summary of meta-analysis results for therapeutic approaches on the outcomes MRI and the histological outcomes on (re-)myelination, inflammation as well as neurodegeneration. The numbers in brackets in the second to fourth columns indicate the number of different publications for each outcome.

| **Therapeutic approach** | **MRI outcome** | **(Re-)myelination outcome** | **Inflammation outcome** | **Neurodegeneration outcome** |
| --- | --- | --- | --- | --- |
| 4-Aminopyridine | Not significant (1) | Not significant (1) | Not significant (1) | Not significant (1) |
| 7D8 (human anti-CD20) | Positive (3) | Not significant (2) | Positive (2) | Not tested |
| ACTH analogue | Positive (1) | Not tested | Not tested | Not tested |
| AdIL-1Beta | Positive (1) | Positive (1) | Positive (1) | Not tested |
| AdIL-1Beta and Interferon Beta | Not significant (1) | Not significant (1) | Not significant (1) | Not tested |
| Anandamide | Positive (1) | Not tested | Not tested | Not tested |
| Alpha-4 subunit of a4pl integrin-antibody | Positive (1) | Not tested | Not tested | Not tested |
| Bu Shen Yi Sui capsule | Positive (1) | Not tested | Not significant (1) | Not tested |
| Cannabidiol | Not significant (1) | Not significant (1) | Not significant (1) | Not tested |
| CD-18-antibody and dexamethasone | Positive (1) | Not tested | Not tested | Not tested |
| CSF-1 receptor kinase inhibitor | Not significant (1) | Nost significant (1) | Positive (1) | Not tested |
| Anti-Lingo-1-antibody | Not significant (1) | Not significant (1) | Not tested | Not tested |
| Bifunctional peptide inhibitor | Positive (1) | Not tested | Not tested | Not tested |
| CT301 (small molecule which binds to a4integrin) | Positive (1) | Not significant (1) | Positive (1) | Not tested |
| CXCR7 antagonist | Not significant (1) | Not tested | Positive (1) | Not significant (1) |
| Fingolimod | Positive (5) | Not significant (2) | Positive (1) | Not tested |
| Gelsolin | Positive (1) | Not tested | Not tested | Not tested |
| Glatiramer acetate | Positive (2) | Not significant (1) | Not tested | Not tested |
| Glatiramer acetate and Salirasib | Positive (1) | Positive (1) | Not tested | Not tested |
| Hyperbaric oxygen treatment | Not significant (1) | Not tested | Not tested | Not tested |
| ICAM-1 antibody | Not significant (1) | Not tested | Positive (1) | Not tested |
| Indazole chloride (estrogen receptor β ligand) | Not significant (1) | Not significant (1) | Not tested | Not tested |
| Interleukin-11Rα Fc | Positive (1) | Not tested | Not tested | Not tested |
| Interleukin-12p40-antibody | Not significant (1) | Not significant (1) | Positive (1) | Not tested |
| Interleukin-17A-antibody | Positive (2) | Not tested | Not tested | Not tested |
| Insulin-like growth factor 1 | Positive (1) | Not tested | Not tested | Not tested |
| Kaliotoxin and ShK | Not significant (1) | Not tested | Not tested | Not tested |
| Methylprednisolone | Positive (2) | Not significant (1) | Not significant (2) | Not significant (1) |
| Alpha-4-integrin-antibody (mouse IgG) | Not significant (1) | Not significant (1) | Not significant (1) | Not tested |
| Nasal myelin oligodendrocyte glycoprotein | Positive (1) | Not tested | Not tested | Not tested |
| Natalizumab | Not significant (1) | Not significant (1) | Not significant (1) | Not tested |
| Nicotine Bitartrate | Positive (1) | Not tested | Not tested | Not tested |
| Normobaric oxygen therapy | Positive (1) | Positive (1) | Not tested | Not tested |
| Olesoxime | Positive (2) | Positive (2) | Not tested | Positive (1) |
| Palmitoyl chloride PAL68–86 | Positive (1) | Not tested | Not tested | Not tested |
| Plexin-A1 antagonist peptide | Not significant (1) | Not significant (1) | Not tested | Not tested |
| Polyunsaturated fatty acid diet | Positive (1) | Not tested | Not tested | Not tested |
| Quetiapine | Positive (1) | Not significant (1) | Not tested | Not tested |
| rHIgM22 | Positive (1) | Not tested | Not tested | Not tested |
| Ropren | Positive (1) | Not tested | Not tested | Not tested |
| Salirasib | Not significant (1) | Positive (1) | Not tested | Not tested |
| Sildenafil | Positive (1) | Positive (1) | Not tested | Not tested |
| Teriflunomide | Not significant (1) | Not tested | Not tested | Not tested |
| Triiodothyronine | Positive (1) | Not tested | Not tested | Not tested |
| Vedolizumab | Not significant (1) | Not significant (1) | Not significant (1) | Not tested |
| VLA-4 antibody (anti-rat) | Not significant (1) | Not tested | Positive (1) | Not tested |
| W-conotoxin GVIA | Positive (1) | Not tested | Not tested | Not tested |
